# ThDP-dependent enzyme catalyzed oxidation of aldehydes

**DOI:** 10.1039/d5sc03250d

**Published:** 2025-08-06

**Authors:** Xiaoyang Chen, Meiting Zhou, Xinyu Duan, Yuting Zhang, Xiaohe Chu, Jian Xu

**Affiliations:** a Collaborative Innovation Center of Yangtze River Delta Region Green Pharmaceuticals, Zhejiang University of Technology Hangzhou 310014 China chenxychem@zjut.edu.cn chuxhe@zjut.edu.cn; b College of Biotechnology and Bioengineering, Zhejiang University of Technology Hangzhou 310014 China jianxu@zjut.edu.cn; c College of Biological, Chemical Science and Engineering, Jiaxing University Jiaxing 314001 China

## Abstract

Natural thiamine diphosphate (ThDP)-dependent enzymes are frequently utilized to catalyze the decarboxylation of β-keto acids and the benzoin condensation of aldehydes. Herein, we present a ThDP-dependent enzymatic oxidation of aldehydes mediated by sequential single electron transfer (SET) processes, utilizing hexachloroethane (C_2_Cl_6_) as the oxidant. The reaction exhibits high efficiency (yield up to 99% and turnover number up to 2000) and achieves effective stereoselective control for dynamic kinetic resolutions (e.e. up to 99%). This study uncovers a previously undiscovered capability of ThDP-dependent enzymes, thus broadening the functional repertoire of this enzyme class.

## Abstract 2

A ThDP-dependent enzymatic oxidation of aldehydes has been developed, proceeding *via* sequential single electron transfer processes. This transformation achieves up to 99% yield, a turnover number of 2000, and up to 99% e.e.

## Graphical abstract

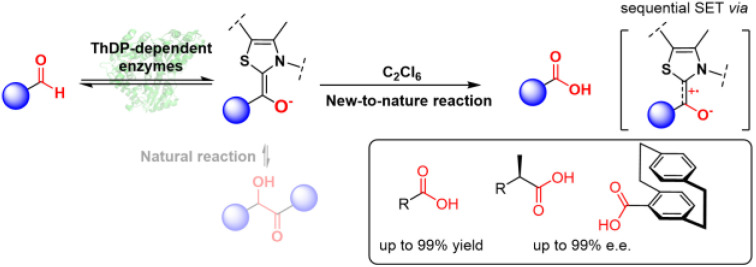

## Introduction

**Fig. 1:**
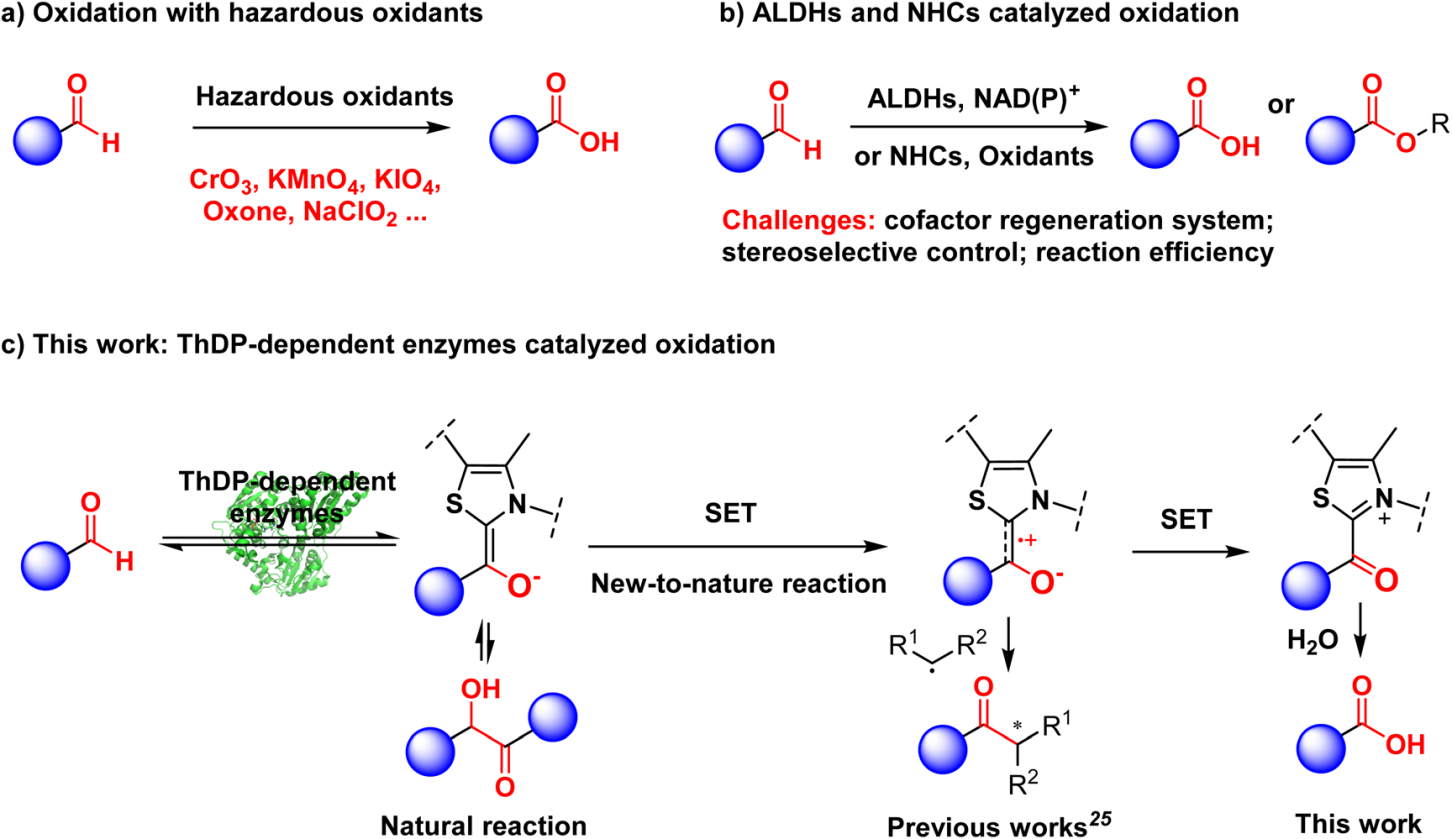
Oxidation of aldehydes to carboxylic acids. (a) Oxidation with hazardous oxidants. (b) ALDHs and NHCs catalyzed oxidation. (c) This work: ThDP-dependent enzymes catalyzed oxidation.

Oxidation is a fundamental chemical reaction, with the conversion of aldehydes to carboxylic acids being one of the most established and widely employed methodologies.^1^ Although synthesizing carboxylic acids by oxidizing their corresponding aldehydes is straightforward, achieving efficient and environmentally friendly processes remains a significant challenge.^2^ Currently, many synthetic methods rely on stoichiometric amounts of hazardous oxidants, including dichromate,^3^ permanganate,^4^ periodate reagents,^5^ oxone^6^ and sodium chlorite (Pinnick oxidation)^7^ (Fig. 1a).

Nature employs a diverse array of strategies for carboxylic acid formation,^8^ with the enzymatic oxidation of aldehydes—exemplified by the remarkable activity of aldehyde dehydrogenases (ALDHs)—standing out as particularly efficient.^9^ These enzymes exhibit catalytic versatility toward a wide spectrum of both aliphatic and aromatic aldehydes, thereby establishing their potential as eco-compatible catalysts in organic synthetic transformations.^10^ Nevertheless, the inherent reliance on NAD(P)^+^ cofactor regeneration systems and the challenges in stereochemical control significantly limit their broader implementation in synthetic applications^11^ (Fig. 1b). On the other hand, the field of biocatalysis is experiencing a transformative revolution, driven by groundbreaking discoveries of enzymatic mechanisms. Recent advances have illuminated several remarkable biocatalytic pathways of oxidations and dehydrogenations, such as the monooxygenation capability of haem peroxygenase,^12^ the desymmetric dehydrogenation function of ene reductase,^13^ and the superoxide mechanism of haem catalase.^14^ Hence, the design and implementation of a more practical biocatalytic approach for aldehyde oxidation,^15^ particularly with precise stereoselective control, is a highly anticipated breakthrough in the advancement of biocatalysis.

Natural enzymes that employ thiamine diphosphate (ThDP) as a cofactor are capable of catalyzing decarboxylation reactions of β-keto acids as well as benzoin condensation reactions.^16^ ThDP is structurally categorized as an *N*-heterocyclic carbene (NHC) derivative.^17^ NHCs exhibit remarkable catalytic versatility, driving a wide range of chemical transformations,^18^ including the oxidation of aldehydes.^19^ The mechanism involves utilizing NHCs to convert the carbon of aldehydes from an electrophile to a nucleophile through the formation of a Breslow intermediate.^20^ Subsequently, various oxidizing agents can extract electrons from this electron-rich Breslow intermediate, resulting in the formation of oxidized products.^21^ However, NHC-catalyzed oxidation reactions face persistent challenges in enhancing catalytic efficiency (typically, catalyst loadings of 5–20 mol % are required. Fig. 1b).^19*d*^ In recent years, the integration of significant intermediates from organic synthesis into enzymes to develop enzyme-catalyzed new-to-nature reactions has emerged as a prominent area of research.^22^ Inspired by the nucleophilicity of Breslow intermediates, our group has developed ThDP-dependent enzyme-catalyzed carbon–carbon bond formation^23^ and hydrogen–deuterium exchange.^24^ Huang pioneered the development of an innovative ThDP-dependent enzymatic system that facilitates radical coupling reactions *via* a key radical cation intermediate.^25*a*,*b*^ Shortly afterward, Yang,^25*c*^ Hayashi^25*d*^ and Fasan^25*e*^ further expanded this system. Very recently, Huang's group reported an electroenzymatic oxidation of aldehydes to carboxylic acids.^26^ In this study, we propose utilizing appropriate oxidants to convert Breslow intermediates *via* sequential single electron transfers (SETs) within the active site of ThDP-dependent enzymes. This transformation will enable nucleophilic attack by water, thereby facilitating aldehyde oxidation reactions catalyzed by ThDP-dependent enzymes (Fig. 1c). Furthermore, we can utilize the specific substrate pockets of enzymes to gain stereoselective control over this reaction.

## Results and discussion

To validate our proposal, we selected 4-methoxybenzaldehyde as the model substrate and conducted a series of screenings to identify ThDP-dependent enzymes and oxidants. The reactions were carried out with cell lysates. As shown in Table 1, enzymes with small binding pockets, such as pyruvate decarboxylase from *Acetobacter pasteurianus* (*ap*PDC) and benzoylformate decarboxylase (BFD), demonstrated low to good reactivity with C_2_Cl_6_ as an oxidizing agent. Benzaldehyde lyase from *Pseudomonas fluorescens* biovar I (*pf*BAL), characterized by its large binding pocket, exhibited remarkable compatibility with a range of oxidizing agents. Different co-solvents, buffers and temperatures influenced the reaction slightly, and the highest yield was obtained with C_2_Cl_6_ as the oxidizing agent using 5% toluene as co-solvent in pH 8.0 MOPS buffer (Tables S1–S3). The enzyme had an excellent turnover number (TON) of 2000, which was higher than that of NHC organocatalysts (typically <100). Control experiments showed that the absence of the enzyme or oxidants prevented the reaction from occurring.

**Table 1:** Optimization of the reaction conditions^a^

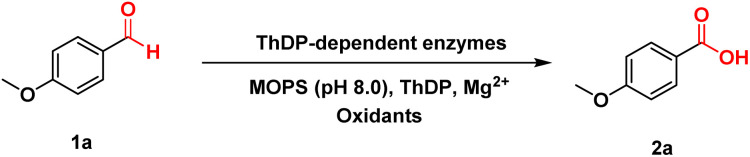			
Entry	Enzymes	Oxidants	Yield/%
1	*Ap*PDC	C_2_Cl_6_	43
2	BFD	C_2_Cl_6_	83
3	*pf*BAL	C_2_Cl_6_	88/99^b^
4	*pf*BAL	DQ	93^b^
5	*pf*BAL	NaClO	81^b^
6	*pf*BAL	H_2_O_2_	80^b^
7	*pf*BAL	CCl_3_Br	90^b^
8	—	C_2_Cl_6_	Trace
9	*pf*BAL	—	Trace

a Reaction conditions: 5 mM 1a, 10 mM oxidants, 2.5 mM MgSO_4_, 0.15 mM ThDP in 50 μL DMSO and 950 μL crude cell extract (pH 8.0 MOPS buffer), 12 h, and 30 °C. DQ = 3,3′,5,5′-tetra-*tert*-butyldiphenylquinone.

b 50 μL toluene instead of DMSO.

With the optimized reaction conditions in hand, we assessed the scope of the ThDP-dependent enzyme-catalyzed aldehyde oxidation reaction. As shown in Table 2, most benzaldehyde derivatives containing substituents at different positions on the aryl group demonstrated favorable reaction outcomes (2a–2j). Furthermore, the electronic effects had a negligible impact, enabling good compatibility with both electron-withdrawing and electron-donating substituted substrates. The low conversion efficiency of 4-hydroxybenzaldehyde (1g) may be attributed to its structural incompatibility with the hydrophobic binding pocket of the enzyme.^27^ Notably, substrates containing alkynes (2h) could be effectively converted into their corresponding products while maintaining the integrity of the alkyne structure. Substrates substituted with ferrocene also exhibited good reactivity (2k). Encouraged by these results, we turned our attention to cinnamaldehyde derivatives (2l–2s). This reaction exhibited significant compatibility with this type of substrate, leading to a diverse array of products that possess substituents with differing positions and electronic properties on the benzene ring. In addition, we conducted a study on aliphatic aldehydes without conjugative effects, as their oxidation was generally considered more challenging. However, the reaction efficiency of hexanal was relatively low, resulting in only 9% yield (2t). The branched aliphatic aldehyde 1u reacted efficiently to achieve the corresponding product with 99% yield. Similarly, 3-phenylpropanal (2v) was also accepted by *pf*BAL, yielding 97% of the desired product. Furthermore, this reaction is easily scalable, enabling the efficient conversion of 100 mg of 1a into the corresponding acid in 91% isolated yield (102 mg 2a).

**Table 2:** Substrate scope of *pf*BAL catalyzed oxidation^a^

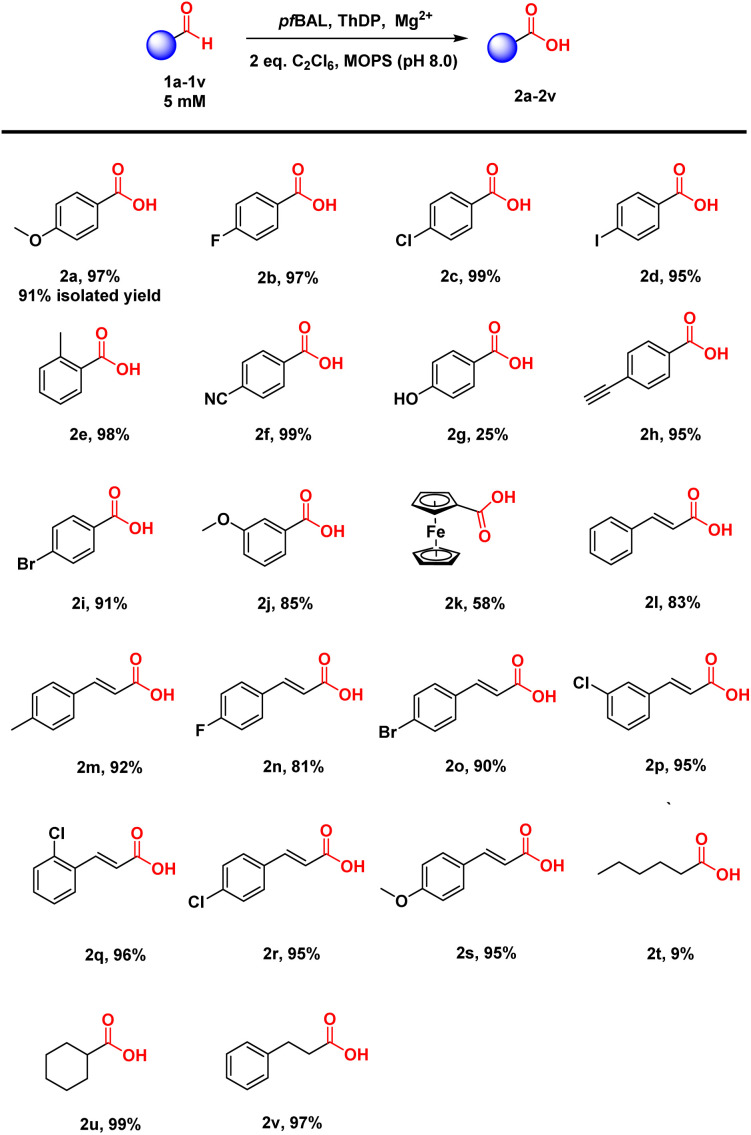

a Reaction conditions: 5 mM 1, 10 mM C_2_Cl_6_, 2.5 mM MgSO_4_, 0.15 mM ThDP in 50 μL toluene and 950 μL crude cell extract (pH 8.0 MOPS buffer, containing about 0.5 mol% *pf*BAL), 4–12 h, and 30 °C. The yields were determined by GC or HPLC.

**Fig. 2:**
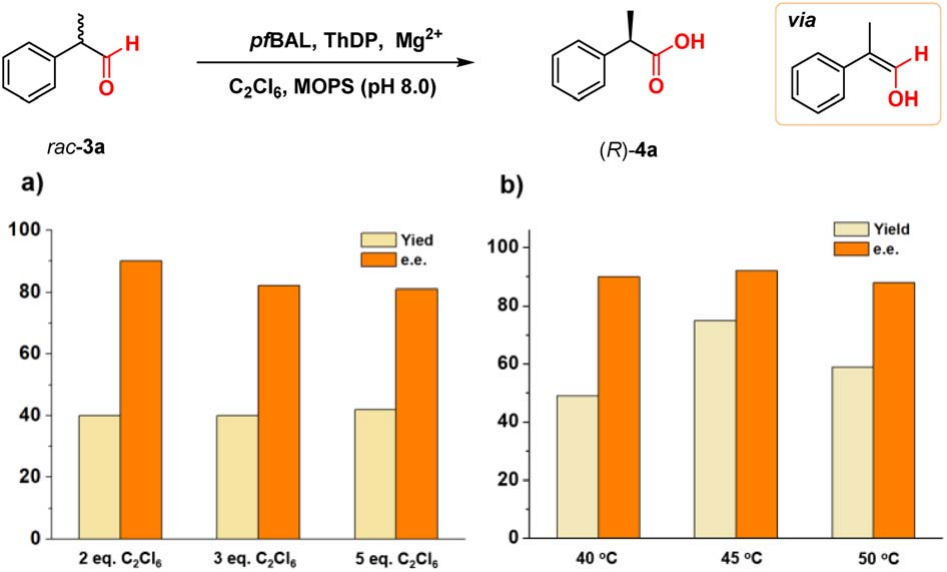
*pf*BAL catalyzed oxidation of 3a. (a) Optimization of the amount of C_2_Cl_6_. (b) Optimization of the reaction temperature. The yields and e.e. values were determined by GC.

Considering the structural specificity of the enzymatic binding pocket, we endeavored to achieve stereoselective transformations in these reactions. 2-Phenylpropanal was chosen as the model substrate due to the rapid racemization of its chiral center under reaction conditions. Our objective was to utilize *pf*BAL-catalyzed aldehyde oxidation to achieve the dynamic kinetic resolution of this compound. To our delight, the use of 2 equivalents of C_2_Cl_6_ as the oxidant resulted in 40% yield and 90% e.e. of the corresponding product (*R*)-4a under the catalysis of a purified enzyme (Fig. 2a). Further increasing the oxidant concentration did not enhance the yield; instead, it led to a significant decrease in stereoselectivity. This decline was likely attributed to an increase in background oxidation reactions. We then investigated the influence of elevated reaction temperatures on the reaction. As shown in Fig. 2b, increasing the temperature significantly improved the reaction yield. The optimal temperature was determined to be 45 °C, where the yield reached 75% and the e.e. was 92%. The yield and selectivity decreased when the temperature exceeded 50 °C, likely due to the destabilization of the enzyme's structure at elevated temperatures (Fig. 2b).

To validate our concept, we evaluated a range of substrate types, including those with isobutyl substitutions, which showed promising yields and stereoselectivity ((*R*)-4b, 53% yield and 99% e.e.). Notably, the planar chiral product [2.2]paracyclophane-4-carboxylic acid (4d) exhibited 28% yield and 82% e.e. *via* a kinetic resolution process. These results emphasized the ability of ThDP-dependent enzymes to control stereoselectivity in this reaction (Table 3).

**Table 3:** Substrate scope of *pf*BAL catalyzed stereoselective oxidation^a^

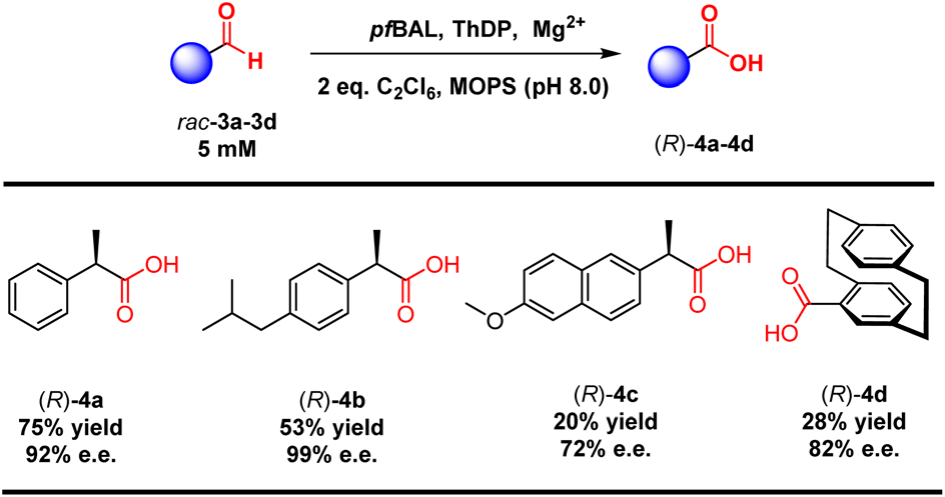

a Reaction conditions: 5 mM 3, 10 mM C_2_Cl_6_, 2.5 mM MgSO_4_, 0.15 mM ThDP, 0.3 mol% purified *pf*BAL in 100 μL toluene and 900 μL pH 8.0 MOPS buffer, 12 h, and 45 °C. The yields and e.e. values were determined by GC or HPLC.

**Fig. 3:**
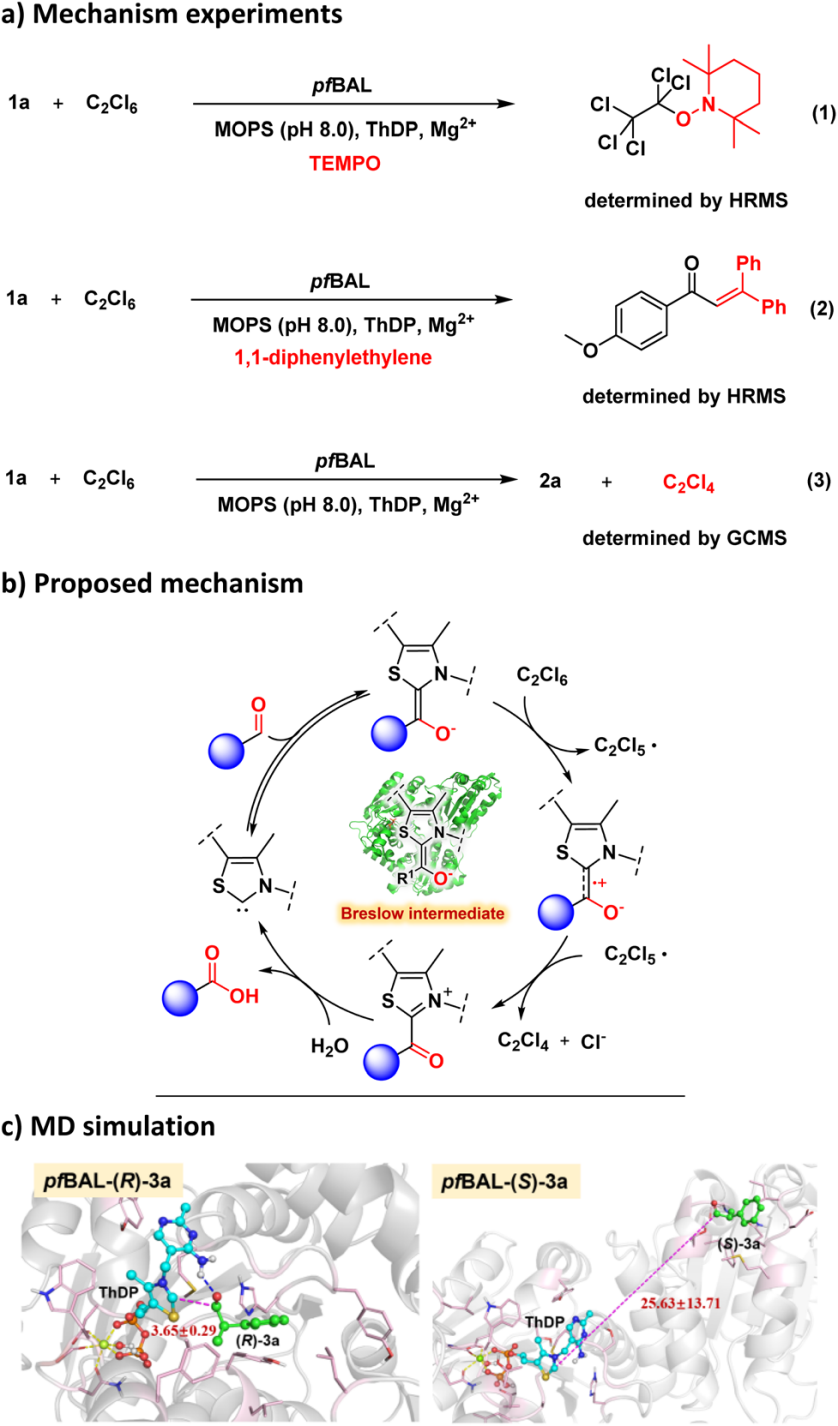
(a) Mechanism experiments, (b) proposed mechanism, and (c) MD simulation of *pf*BAL with 3a; PDB: 3D7K.^27^

To elucidate the mechanism of this enzymatic oxidation reaction, we incorporated 2,2,6,6-tetramethylpiperidine-1-oxyl (TEMPO) and 1,1-diphenylethylene into the model reactions for the radical capture experiments and observed the adducts formed between radical intermediates and radical traps (Fig. 3a, S1 and S2). This result demonstrated that the reaction proceeded *via* a SET mechanism. Additionally, the reduction product C_2_Cl_4_ could be determined by GCMS in the model reaction (Fig. S3). We proposed the mechanism illustrated in Fig. 3b. The oxidation process initiated with the formation of a Breslow intermediate, which underwent two sequential SET processes in the presence of the oxidant C_2_Cl_6_. These processes resulted in the generation of an acyl azolium intermediate, which was subsequently hydrolyzed under aqueous conditions to yield the corresponding carboxylic acids, ultimately leading to the release of ThDP.

Finally, we conducted molecular docking calculations and molecular dynamics (MD) simulations to gain insight into the origin of stereoselectivity in the *pf*BAL-catalyzed aldehyde oxidation reaction (Fig. S4–S7). As illustrated in Fig. 3c, the (*R*)-enantiomer of 3a exhibited stable binding within the enzyme's active site, maintaining a catalytic distance of 3–4 Å (from ThDP to the carbonyl group of the substrate). In contrast, the (*S*)-enantiomer failed to achieve stable binding and was observed to dissociate from the enzyme's binding pocket during the MD simulations. From the substrate–protein interaction perspective (Fig. S5 and S7), *pf*BAL-Rgs forms stronger contacts than *pf*BAL-Sgs, ensuring tighter binding. In *pf*BAL-Rgs, a hydrogen bond between the ligand's carbonyl and the cofactor anchors the substrate in an optimal pre-reaction conformation. In contrast, the lack of this bond in *pf*BAL-Sgs allows the *S*-type substrate to quickly dissociate. In addition, binding free energy calculations revealed that (*R*)-3a exhibited enhanced stabilization when interacting with the enzyme (*pf*BAL-(*R*)-3a, −35.39 kcal mol^−1^*vs. pf*BAL-(*S*)-3a, −30.84 kcal mol^−1^). These findings aligned with the experimental results.

## Conclusions

In conclusion, we have developed a ThDP-dependent enzyme-catalyzed aldehyde oxidation reaction. This study utilizes the ThDP cofactor in *pf*BAL to bind with the substrate and produce an electron-rich Breslow intermediate, with C_2_Cl_6_ acting as the oxidizing agent to facilitate the oxidation of aldehyde. The reaction exhibits high efficiency and offers stereoselective control for chiral substrates. Mechanistic experiments are consistent with a single electron oxidation mechanism, and MD simulations have been employed to elucidate the origin of stereoselectivity. This study reveals a previously unreported function of ThDP-dependent enzymes, thus broadening the functional repertoire of this enzyme class.

## Author contributions

J. X. and X. C. directed the project. X. C. and M. Z. developed the reactions and performed the majority of synthetic experiments. X. D. and Y. Z. assisted with synthetic experiments. J. X. and X. C. wrote the paper with input from all authors.

## Conflicts of interest

There are no conflicts to declare.

## Supplementary Material

SC-OLF-D5SC03250D-s001

## Data Availability

The data supporting this article have been included as part of the SI. Experimental methods, optimization data, protein expression, computational details, chemical characterization and NMR spectra. See DOI: https://doi.org/10.1039/d5sc03250d.
