# Supplementary material for: ThDP-dependent enzyme catalyzed oxidation of aldehydes
Source: Chem Sci. 2025 Aug 6;16(35):15977–81. doi: 10.1039/d5sc03250d (PMC12352684; doi:10.1039/d5sc03250d)
Supplement: SC-016-D5SC03250D-s001 [file SC-016-D5SC03250D-s001.pdf]

Supplementary Information (SI) for Chemical Science.
This journal is © The Royal Society of Chemistry 2025

ThDP-dependent Enzymes Catalyzed Oxidation of Aldehydes

Xiaoyang Chen*, Meiting Zhou, Xinyu Duan, Yuting Zhang, Xiaohe Chu* and Jian Xu*

Table of Contents

Contents	Pages
Experimental procedures	S2
General information	S2
Protein expression	S2
Protein purification	S2
General procedure for the enzymatic reactions for **2a-v**	S3
General procedure for the enzymatic reactions for **4a-d**	S3
Determination of turn over number (TON)	S4
Scaling-up experiment	S4
General procedure for the synthesis of aldehydes	S4
General procedure for the synthesis of acids via chemical routes	S5
Gene sequence of *pf*BAL	S5
Protein sequence of *pf*BAL	S6
Computational details	S6
**Table S1** The screening of co-solvents	S9
**Table S2** The screening of buffers	S9
**Table S3** The screening of temperatures	S9
**Table S4** Summary of all available crystal structures for *pf*BAL	S10
**Figure S1** The HRMS spectrum of TEMPO adduct	S11
**Figure S2** The HRMS spectrum of 1,1-diphenylethylene adduct	S11
**Figure S3** The MS spectrum of C_2Cl_4	S11
**Figure S4** Comparative analysis of substrate binding poses of *pf*BAL-Rgs and *pf*BAL-Sgs via molecular docking.	S12
**Figure S5** MD analysis of the stereoselective preference mechanism of *pf*BAL	S12
**Figure S6** The coordination states of metal Mg^{2+} ions in MD simulation process of *pf*BAL-Ris, *pf*BAL-Rgs, *pf*BAL-Sgs	S12
**Figure S7** Monitoring the key distances change between the ThDP cofactor and the substrate in the molecular MD trajectories of *pf*BAL-Rgs, *pf*BAL-Sgs	S13
Characterization data and calibration curves	S14
NMR spectra	S28
Chromatographic data	S57
References	S62

Experimental procedures

General information

All chemical reagents were obtained from commercial sources unless otherwise noted. Aldehydes **1a**-**1v** and **3a** were purchased from Shanghai Titan Scientific Co., Ltd.. The ^{1}H and ^{13}C NMR spectra were recorded with a Bruker AMX400 MHz spectrometer using TMS as an internal standard. The high resolution mass spectrum (HRMS) was obtained on Waters G2-XS Qtof. Enantiomeric purities of the reaction products were analyzed by GC with CP-Chirasil-Dex CB column (25 m × 0.25 mm × 0.25 μm) or HPLC with Daicel Chiralcel AD-H using *n*-hexanes/isopropanol as the mobile phase. Absolute configurations were confirmed by comparison with literature values.

Protein expression

An E. coli BL21 (DE3) single colony harboring pET-22b (+)-*pf*BAL was first inoculated in 5 ml LB medium (containing 100 μg/mL ampicillin), and was shaken at 200 rpm overnight as preculture. The preculture was used to inoculated large culture (250 mL TB + 100 μg/mL ampicillin in 1 L shake flasks) at 37 ^{o}C for about 4 hours until OD (600) at 0.6～0.8. After cooling at 4 ^{o}C for 0.5 h, 1 mM isopropyl β-thiogalactopyranoside (IPTG) was added to induce *pf*BAL expression. The culture was allowed to express in 20 ^{o}C for 24 h at 200 rpm. Then, cells were harvested by centrifugation at 9000 rpm and 4 ^{o}C for 10 min and the supernatants were discarded. The cells were resuspended in MOPS-buffer (1 g wet cell in 5 mL 50 mM pH 8.0) and stored in -80 ^{o}C. The cells were repeated freezing and thawing for 3 times, and then released the target proteins by sonication. The cell debris were removed by centrifugation at 12,000 rpm for 15 min at 4 ^{o}C, the enzyme solutions were stored at -80 ^{o}C for further reaction. The concentration of *pf*BAL was calculated according to SDS-PAGE of cell lysate as 0.025 mM (1.5 mg/mL).

Protein purification

The crude enzyme solution was filtered and loaded on a GE Healthcare HisTrap FF Crude column (5 mL) preequilibrated with 50 mM MOPS-buffer (pH 8.0) containing 0.5 M NaCl and 10 mM imidazole. The enzyme was eluted by 50 mM phosphate buffer with 0.5 M NaCl and 200 mM imidazole (5 column volumes). The pure enzyme solution was obtained after ultrafiltration for three times by 50 mM MOPS-buffer (pH 8.0). After purification, the concentration of enzymes was determined by the absorption at 595 nm according to the calibration curve of BSA. Then, the purified enzyme was used immediately for the synthesis of chiral products (0.015 mM, 0.3 mol% in 5 mM scale reactions).

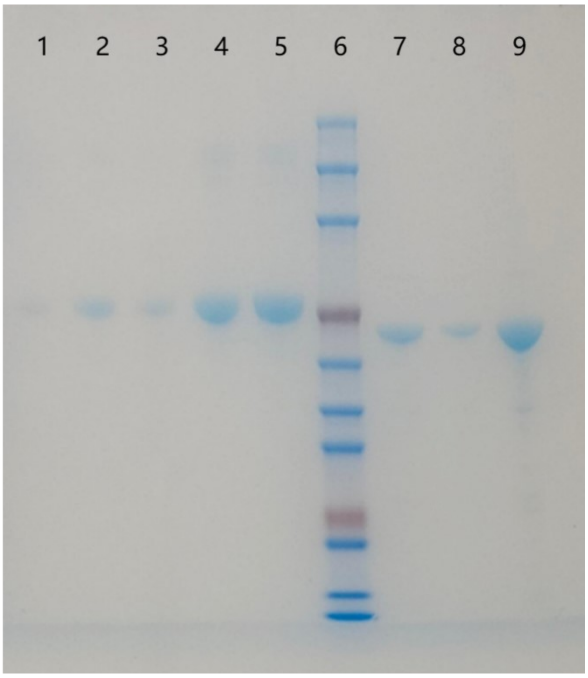

SDS-PAGE of *pf*BAL. Lane 1-5: BSA, lane 6: protein markers, lane 7-9: purified *pf*BAL

General procedure for the enzymatic reactions for 2a-2v

0.005 mmol substrate **1** and 0.01 mmol C_2Cl_6 was dissolved in 50 µL toluene, then was added into 950 µL crude cell extract (MOPS buffer 50 mM, pH 8.0, containing 2.5 mM $MgSO_4$, 0.15 mM ThDP and 0.025 mM *pf*BAL). The mixture was shaken at 800 rpm for 4-12 h (4 h for **1a-1f**, **1h**, **1p-1s**, **1u**, **1v**, and 12 h for others) at 30 °C.

For **2a-2j** and **2i-2s**, the reactions were quenched with 1 mL CH_3CN. After vigorous mixing, reaction mixtures were centrifuged at 12,000 rpm for 5 min. The yields were determined by HPLC with the aid of calibration curves. The HPLC analysis method is as follows: HITACHI LaChrom C18 column (5 µm, 4.6 × 250 mm), flow rate = 1.0 mL/min, $CH_3CN/H_2O/HCOOH$ (50/50/0.5) as the mobile phase.

For **2k**, the reaction was quenched with 200 µL 3 M HCl solution and extracted with 1 mL ethyl acetate for three times. Then, the organic solution was concentrated in vacuo, and the yield was determined by HPLC with the aid of calibration curve. The HPLC analysis method is as follows: Daicel Chiralcel AD-H column (250 × 4.6 mm), flow rate = 1.0 mL/min, *n*-hexanes/isopropanol/trifluoroacetic acid (90/10/0.1) as the mobile phase.

For **2t-2v**, the reactions were quenched with 200 µL 3 M HCl solution and extracted with 1 mL ethyl acetate for three times (0.005 mmol Phenylacetic acid was added as the internal standard). Then, the yields were determined by GC with the aid of calibration curves. The GC analysis method is as follows: CP-7502 column, T_0: 100 °C, dT/dt: 5 °C/min, T_1: 180 °C.

General procedure for the enzymatic reactions for 4a-4d

0.005 mmol substrate **3** and 0.01 mmol C_2Cl_6 was dissolved in 100 µL toluene, then was added into 900 µL purified *pf*BAL solution (MOPS buffer 50 mM, pH 8.0, containing 0.015 mM protein, 2.5 mM $MgSO_4$ and 0.15 mM ThDP). The mixture was shaken at 800 rpm for 12 h at 45 °C.

For **4a** and **4b**, the reactions were quenched with 200 μL 3 M HCl solution and extracted with 1 mL ethyl acetate for three times (0.005 mmol Phenylacetic acid was added as the internal standard). The yields and e.e. values were determined by GC with the aid of calibration curves. The GC analysis method is as follows: CP-7502 column, T_0: 120 ^oC, dT/dt: 1 ^oC/min, T_1: 180 ^oC.

For **4c**, the reaction was quenched with 200 μL 3 M HCl solution and extracted with 1 mL ethyl acetate for three times. Then, the organic solution was concentrated in vacuo, and the yield and e.e. value were determined by HPLC with the aid of calibration curve. The HPLC analysis method is as follows: Daicel Chiralcel AD-H column (250 × 4.6 mm), flow rate = 1.0 mL/min, *n*-hexanes/isopropanol/trifluoroacetic acid (90/10/0.1) as the mobile phase.

For **4d**, the reaction was quenched with 200 μL 3 M HCl solution and extracted with 1 mL ethyl acetate for three times. Then, the organic solution was concentrated in vacuo, and the yield and e.e. value were determined by HPLC with the aid of calibration curve. The HPLC analysis method is as follows: Daicel Chiralcel AD-H column (250 × 4.6 mm), flow rate = 1.0 mL/min, *n*-hexanes/isopropanol (90/10) as the mobile phase.

Determination of turn over number (TON)

TON for *pf*BAL in model reaction was determined in the condition of general procedure, but purified enzyme was used as catalyst. The protein concentration was calculated according to the standard curve of BSA. The TON was calculated by the formula: $Conc._{(product)}/Conc._{(Catalyst)}$.

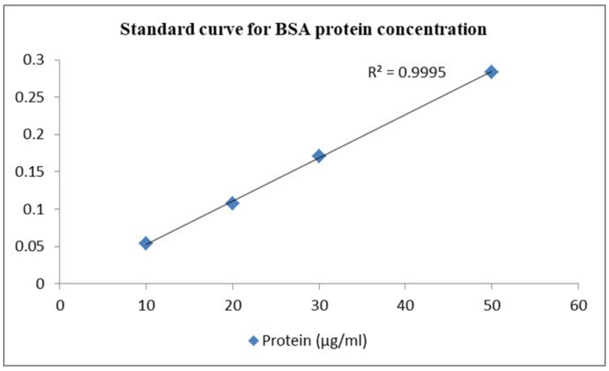

Scaling-up experiment

The scale-up reaction of **1a** was performed as follows: to a 250 mL flask containing 80 mL crude cell extract (16g wet cell resuspended in 80 mL pH 8.0 MOPS-buffer with 0.2 mmol $MgSO_4$ and 0.012 mmol ThDP) was added 50 mL toluene solution with 0.7 mmol **1a** and 1.4 mmol C_2Cl_6. The mixture was shaken at 200 rpm and 30 ^oC for 12 h, then quenched with 16 mL 3 M HCl solution and extracted with 80 mL ethyl acetate for three times. The organic layer was dried over $MgSO_4$ and concentrated in vacuo, then purified by column chromatography (silica gel, petroleum ether/EtOA/HCOOH = 70/30/0.1), providing **2a** in 91% yield.

General procedure for the synthesis of aldehydes:

Method A, synthesis of **3b**, **3c**.

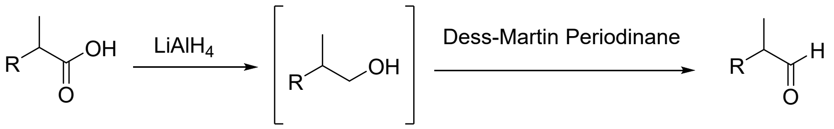

3b, **3c**

A flame-dried Schlenk tube was charged with $LiAlH_4$ in THF (8 mL, 2 eq.). α-branched carboxylic acids (4 mmol, 1 equiv) dissolved in THF (10 mL) was added dropwise into the suspension at 0 °C. After stirring at room temperature for 1 h, the reaction was quenched by the slow dropwise addition of H_2O (2.5 mL), aqueous 1.5 M NaOH (2.5 mL) and H_2O (7.5 mL) at 0 °C. The resultant mixture was treated with Na_2SO_4 and filtered. The filtrate was concentrated to afford alcohols without further purification. To a solution of alcohol (4 mmol, 1 eq.) in dry DCM (10 mL) was added Dess-Martin Periodinane (4.8 mmol, 1.2 equiv) at 0 °C. After stirring at 0 °C for 1 h, the oxidant was quenched by adding a 1:1 mixture of $Na_2S_2O_3$ and $NaHCO_3$ saturated aqueous (20 mL). The aqueous phase was extracted with DCM. The combined organic layers were dried over anhydrous Na_2SO_4, and concentrated in vacuo. The crude product was purified by flash chromatography (silica gel, petroleum ether/EtOAc) to afford aldehydes (26% yield for **3b**, 39% yield for **3c**).

Method B, synthesis of **3d**.

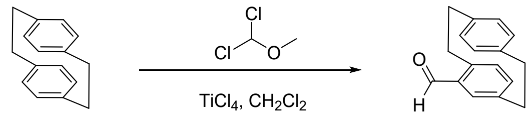

paracyclophane **3d**

To a solution of [2.2]-paracyclophane (1 eq.) in CH_2Cl_2 (0.1 M), equipped with stirred, was sequentially added $TiCl_4$ (2 equiv) and dichloromethoxymethane (1.05 equiv) at 0 °C. The resulting solution was stirred at room temperature for 24 h, followed by the addition of ice water (150 mL) at 0 °C. The reaction mixture was stirred for 1 h and gradually warm up to room temperature. The organic layer was separated and the aqueous layer was washed with CH_2Cl_2, and the combined organic phases were a saturated solution of NaCl, dried over $MgSO_4$, and evaporated in vacuo. The residue was purified by flash chromatography on silica gel with petroleum ether/ethyl acetate as eluent to give the products **3d** (65% yield).

General procedure for the synthesis of acids via chemical routes

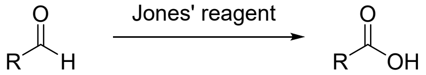

1 or **3** **2** or **4**

To a stirred solution of 5 mmol aldehydes **1** or **3** in 10 mL acetone was slowly added 3.5 mL 1.8 M Jones reagent at 0 °C. After 2 h, solvent was removed, and the pH was adjusted to 13 with 2 M NaOH. The crude mixture was immediately extracted by DCM (3 x15 mL). The aqueous phase was acidized to pH 1 with 2 M HCl. The aqueous phase was extracted by DCM (3 x 15 mL). The combined organic layers were washed with cold brine and dried over $MgSO_4$. Evaporation of the solvent under reduced pressure gave the desired acids **2** or **4** without further purification. The yields typically fell within the range of 85% to 95%.

Gene sequence of *pf*BAL

CATATGGCGATGATTACAGGCGGCGAACTGGTTGTTCGCACCCTAATAAAGGCTGGGGTCGAACATCTGT
TCGGCCTGCACGGCGCGCATATCGATACGATTTTTCAAGCCTGTCTCGATCATGATGTGCCGATCATCGACA
CCCGCCATGAGGCCGCCGCAGGGCATGCGGCCGAGGGCTATGCCCGCGCTGGCGCCAAGCTGGGCGTGGC

GCTGGTCACGGCGGGCGGGGGATTTACCAATGCGGTCACGCCCATTGCCAACGCTTGGCTGGATCGCACGC

CGGTGCTCTTCCTCACCGGATCGGGCGCGCTGCGTGATGATGAAACCAACACGTTGCAGGCGGGGATTGAT

CAGGTCGCCATGGCGGCGCCCATTACCAAATGGGCGCATCGGGTGATGGCAACCGAGCATATCCCACGGCT

GGTGATGCAGGCGATCCGCGCCGCGTTGAGCGCGCCACGCGGGCCGGTGTTGCTGGATCTGCCGTGGGATA

TTCTGATGAACCAGATTGATGAGGATAGCGTCATTATCCCCGATCTGGTCTTGTCCGCGCATGGGGCCAGA

CCCGACCCTGCCGATCTGGATCAGGCTCTCGCGCTTTTGCGCAAGGCGGAGCGGCCGGTCATCGTGCTCGG

CTCAGAAGCCTCGCGGACAGCGCGCAAGACGGCGCTTAGCGCCTTCGTGGCGGCGACTGGCGTGCCGGTGT

TTGCCGATTATGAAGGGCTAAGCATGCTCTCGGGGCTGCCCGATGCTATGCGGGGCGGGCTGGTGCAAAAC

CTCTATTCTTTTGCCAAAGCCGATGCCGCGCCAGATCTCGTGCTGATGCTGGGGGCGCGCTTTGGCCTTAAC

ACCGGGCATGGATCTGGGCAGTTGATCCCCCATAGCGCGCAGGTCATTCAGGTCGACCCTGATGCCTGCGA

GCTGGGACGCCTGCAGGGCATCGCTCTGGGCATTGTGGCCGATGTGGGTGGGACCATCGAGGCTTTGGCGC

AGGCCACCGCGCAAGATGCGGCTTGGCCGGATCGCGGCGACTGGTGCGCCAAAGTGACGGATCTGGCGCA

AGAGCGCTATGCCAGCATCGCTGCGAAATCGAGCAGCGAGCATGCGCTCCACCCCTTTCACGCCTCGCAGG

TCATTGCCAAACACGTCGATGCAGGGGTGACGGTGGTAGCGGATGGTGCGCTGACCTATCTCTGGCTGTCC

GAAGTGATGAGCCGCGTGAAACCCGGCGGTTTTCTCTGCCACGGCTATCTAGGCTCGATGGGCGTGGGCTT

CGGCACGGCGCTGGGCGCGCAAGTGGCCGATCTTGAAGCAGGCCGCCGCACGATCCTTGTGACCGGCGAT

GGCTCGGTGGGCTATAGCATCGGTGAATTTGATACGCTGGTGCGCAAACAATTGCCGCTGATCGTCATCAT

CATGAACAACCAAAGCTGGGGGGCGACATTGCATTTCCAGCAATTGGCCGTCGGCCCCAATCGCGTGACGG

GCACCCGTTTGGAAAATGGCTCCTATCACGGGGTGGCCGCCGCCTTTGGCGCGGATGGCTATCATGTCGAC

AGTGTGGAGAGCTTTTCTGCGGCTCTGGCCCAAGCGCTCGCCCATAATCGCCCCGCCTGCATCAATGTCGCG

GTCGCGCTCGATCCGATCCCGCCCGAAGAACTCATTCTGATCGGCATGGACCCCTTCGCACTCGAG

Protein sequence of *pf*BAL

1 MAMITGGELV VRTLIKAGVE HLFGLHGAHI DTIFQACLDH DVPIIDTRHE AAAGHAAEGY

61 ARAGAKLGVA LVTAGGGFTN AVTPIANAWL DRTPVLFLTG SGALRDDETN TLQAGIDQVA

121 MAAPITKWAH RVMATEHIPR LVMQAIRAAL SAPRGPVLLD LPWDILMNQI DEDSVIIPDL

181 VLSAHGARPD PADLDQALAL LRKAERPVIV LGSEASRTAR KTALSAFVAA TGVPVFADYE

241 GLSMLSGLPD AMRGGLVQNL YSFAKADAAP DLVLMLGARF GLNTGHGSGQ LIPHSAQVIQ

301 VDPDACELGR LQGIALGIVA DVGGTIEALA QATAQDAAWP DRGDWCAKVT DLAQERYASI

361 AAKSSSEHAL HPFHASQVIA KHVDAGVTVV ADGALTYLWL SEVMSRVKPG GFLCHGYLGS

421 MGVGFGTALG AQVADLEAGR RTILVTGDGS VGYSIGEFDT LVRKQLPLIV IIMNNQSWGA

481 TLHFQQLAVG PNRVTGTRLE NGSYHGVAAA FGADGYHVDS VESFSAALAQ ALAHNRPACI

541 NVAVALDPIP PEELILIGMD PFALA

Computational details

Model Preparation and Classical MD Simulations

For *pf*BAL, numerous crystal structures have been resolved to date, as summarized in Table S4. we reconstructed the reliable computational models of *pf*BAL complexed with cofactor ThDP based on the PDB entries 3D7K and 2UZ1. Given that the ligand was missing in the crystal structures, we employed the Glide module in Schrödinger software[1] to perform molecular docking calculations, resulting in complex structures that incorporate cofactor ThDP along with various chiral substrates. Subsequently, we conducted long-term MD simulations to acquire stable representative structures for further analysis. All MD simulations were conducted using AMBER22 software[2], employing the Amber ff14SB force field[3] for the protein and the TIP3P model[4] for the solvent water molecules[5]. The force field parameters for the cofactor ThDP and substrates were derived from the General Amber Force Field[6]. Additionally, the partial atomic charges for the cofactor ThDP and substrates were computed using the restrained electrostatic potential method at the HF/6-31G* level with the Gaussian 16 package[7]. Each model was solvated in cubic boxes with an extension of 10 Å from the protein boundary, and Na+ and Cl- ions were included to neutralize the systems. The initial coordinates and topology files for these models were generated using the leap module in AMBER.

Three initial minimization steps were performed to relax the solvent waters and the protein's side chains using steepest descent and conjugate gradient methods. Subsequently, each system was gradually heated from 0 K to 300 K over a period of 25 ps and maintained at 300 K for an additional 25 ps under an NVT ensemble. Subsequently, a density equilibration simulation lasting 100 ps was conducted at 300 K and a pressure of 1.0 atm under an NPT ensemble. Finally, all models underwent at least 200 ns of simulation under the NVT ensemble with a time step of 2 fs[8]. During the MD simulations, Langevin dynamics were employed to regulate temperature with a collision frequency of 1.0 ps[9]. Long-range electrostatic interactions were calculated using the particle mesh Ewald method[10]. The SHAKE algorithm was utilized to constrain hydrogen bonds, while a cutoff distance of 12 Å was applied for van der Waals and electrostatic interactions[11,12]. The MD trajectories were analyzed using CPPTRAJ, which included assessments of root-mean-square deviations (RMSDs) and cluster analysis[13].

Shedding light on the mechanism of pfBAL enzyme stereoselectivity preferences

To thoroughly investigate the stereoselective preference mechanism of *pf*BAL enzymes, we constructed the ground-state structures for *pf*BAL when interacting with the *R*-substrate, referred to as *pf*BAL-Rgs, and the *S*-substrate, denoted as *pf*BAL-Sgs. Additionally, we constructed the intermediate-state structure corresponding to the *R*-substrate, termed *pf*BAL-Ris. To further our understanding of these interactions, we conducted a comprehensive molecular dynamics simulation analysis, allowing us to observe the dynamic behavior and underlying mechanisms of these enzyme-substrate complexes over time. As illustrated in Figure S3, after conducting 200 ns MD simulations, the RMSD plots for above three models were obtained and showed that the simulations have reached stability. A critical component in this context is the divalent metal ion Mg^{2+}, which plays a significant role in the structural integrity of protein receptors. During the simulations, Mg^{2+} established a coordination complex with the pyrophosphate group of the cofactor, reinforcing the binding interaction of the cofactor. Furthermore, throughout the simulation, Mg^{2+} maintained a stable six-coordinate complex comprising the ThDP, amino acid residue D448/N475/S477, and a water molecule, as depicted in Figure S4.

To deepen our understanding of the mechanisms underlying the enantioselectivity preference exhibited by *pf*BAL, the stable MD trajectories were selected for cluster analysis. This analysis allowed us to extract representative structures that further elucidate the factors contributing to this selective behavior. From the perspective of the interaction characteristics between the substrate and the protein receptor (Figure S3), it was observed that *pf*BAL-Rgs exhibited more interaction forces compared to *pf*BAL-Sgs, resulting in stronger substrate binding. The hydrogen bonding interaction between the carbonyl oxygen atom of the ligand and the cofactor in the *pf*BAL-Rgs model, which is also evident in the *pf*BAL-Ris model (Figure S3), is crucial for stabilizing the ligand within the protein's active pocket, ensuring the substrate remains in

an optimal pre-reaction conformation. In contrast, the *pf*BAL-Sgs structure lacks this hydrogen bond, explaining the swift release of the S-type substrate into the external solvent, even after it initially enters the protein pocket.

Tracking the key striking distances between the ThDP cofactor and the substrate (Figure S5) demonstrated that the ligand in *pf*BAL-Rgs consistently occupies the active catalytic region, effectively positioned for the forthcoming nucleophilic attack. Conversely, the substrate in pfBAL-Sgs only lingered in the pocket for around 50 ns before detaching from the catalytic area, which hindering its ability to engage in subsequent catalytic reactions. Furthermore, binding free energy calculations conducted through MMGBSA analysis highlight this difference. The ΔGbinding for *pf*BAL-Rgs was -35.39 kcal/mol, while *pf*BAL-Sgs it was -30.84 kcal/mol. This significant variation in binding energy highlights the greater affinity between the substrate and the enzyme in *pf*BAL-Rgs, reinforcing its effectiveness in catalytic processes.

Supplemental figures and tables in Supporting Information

Table S1. The screening of co-solvents

Entry	Solvent	Solvent/(v/v%)	Yield/%
1	MTBE	5	75
2	IPA	5	77
3	MeCN	5	87
4	n-Hexane	5	70
5	DMSO	5	88
6	Toluene	5	99
7	Toluene	10	92
8	Toluene	15	80
9	Toluene	20	85

Reaction conditions: 5 mM **1a**, 10 mM C_2Cl_6, 2.5 mM $MgSO_4$, 0.15 mM ThDP in co-solvent and crude cell extract (pH 8.0 MOPS buffer), 12 h, 30 ^{o}C.

Table S2. The screening of buffers

Entry	Buffer	pH	Yield/%
1	MOPS	6.5	88
2	MOPS	7	97
3	MOPS	7.5	93
4	MOPS	8	99
5	MOPS	8.5	98
6	Tris-HCl	8	90
7	KPi	8	87
8	PBS	8	87

Reaction conditions: 5 mM **1a**, 10 mM C_2Cl_6, 2.5 mM $MgSO_4$, 0.15 mM ThDP in 50 μL toluene and 950 μL crude cell extrac, 12 h, 30 ^{o}C.

Table S3. The screening of temperatures

Entry	Temperature/^{o}C	Yield/%
1	25	99
2	30	99
3	35	99
4	40	98

Reaction conditions: 5 mM **1a**, 10 mM C_2Cl_6, 2.5 mM $MgSO_4$, 0.15 mM ThDP in 50 μL toluene and 950 μL crude cell extract (pH 8.0 MOPS buffer), 12 h.

Table S4 Summary of all available crystal structures for *pf*BAL

PDB Entry	Ligand	Resolution(Å)	mutation	Sequence Length
2AG0	MG、TPP	2.58	0	563
2AG1	MG、TPP	2.58	13	563
2UZ1	MPD、TPP	1.65	0	563
3D7K	CA、D7K	2.49	0	570
3IAE	CA、D7K	2.30	1	570
3IAF	MG、TPP	2.80	1	570
4QPZ	MG、TPP	3.00	0	582
4QQ8	MG、TPP	2.88	0	583

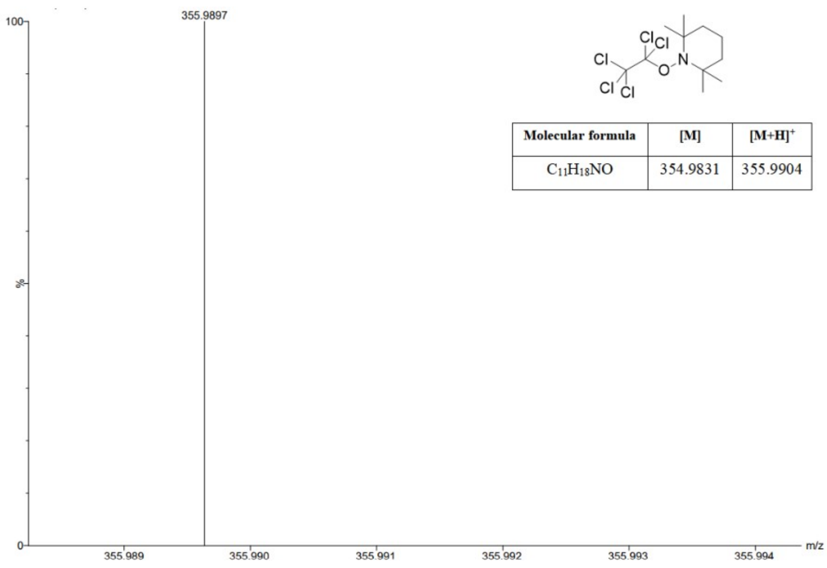

Molecular formula	[M]	[M+H]+
$C_{11}H_{18}NO$	354.9831	355.9904

Figure S1 The HRMS spectrum of TEMPO adduct

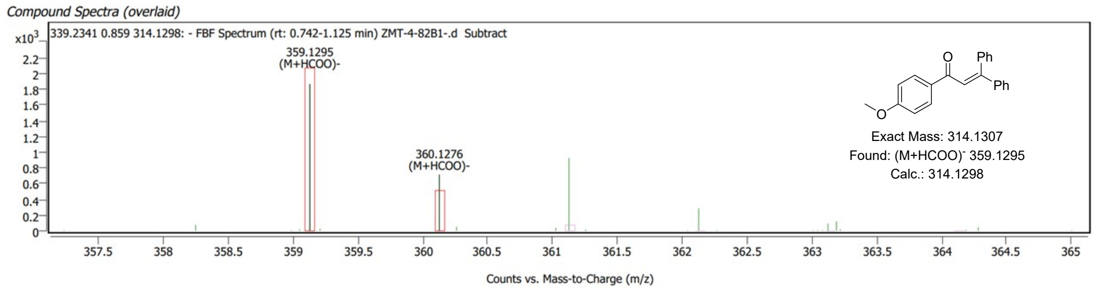

Figure S2 The HRMS spectrum of 1,1-diphenylethylene adduct

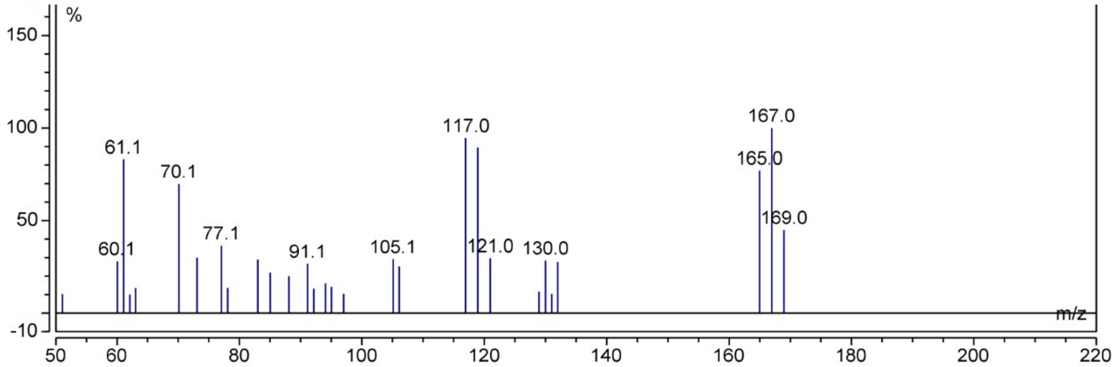

Figure S3 The MS spectrum of C_2Cl_4

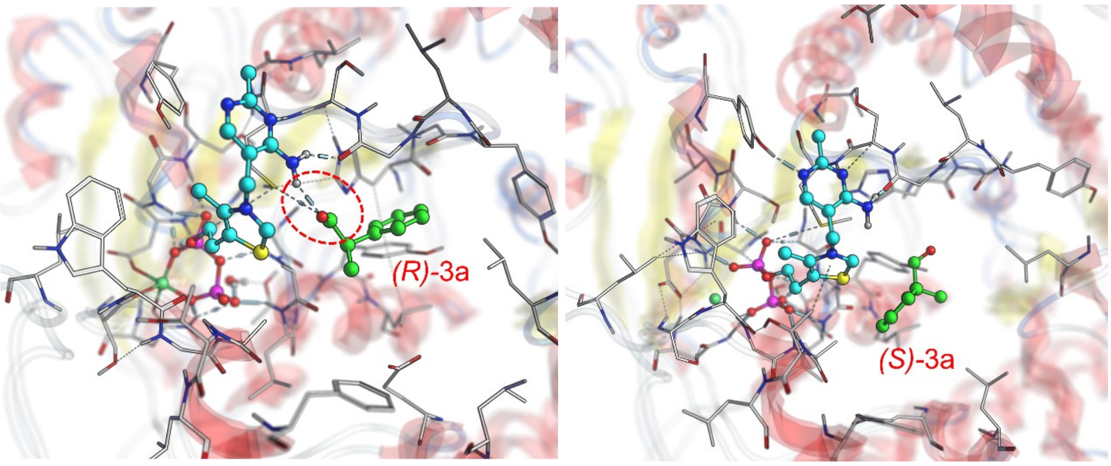

Figure S4 Comparative analysis of substrate binding poses of *pf*BAL-Rgs and *pf*BAL-Sgs via molecular docking.

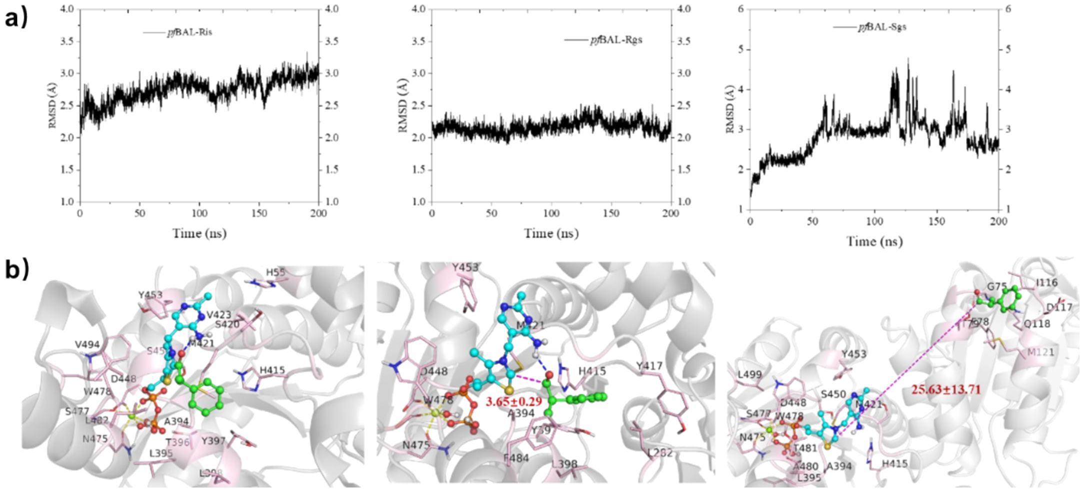

Figure S5 MD analysis of the stereoselective preference mechanism of *pf*BAL. a) RMSD results of *pf*BAL-Ris, *pf*BAL-Rgs, *pf*BAL-Sgs; b) The ligand binding poses of *pf*BAL-Ris, *pf*BAL-Rgs, *pf*BAL-Sgs. The key striking distance and the hydrogen bond interaction was shown as the dashed rosy line and blue line.

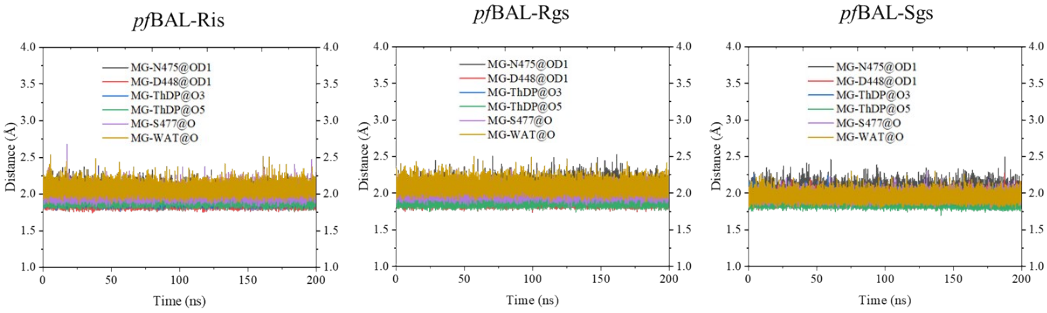

Figure S6 The coordination states of metal Mg^{2+} ions in MD simulation process of *pf*BAL-Ris, *pf*BAL-Rgs, *pf*BAL-Sgs.

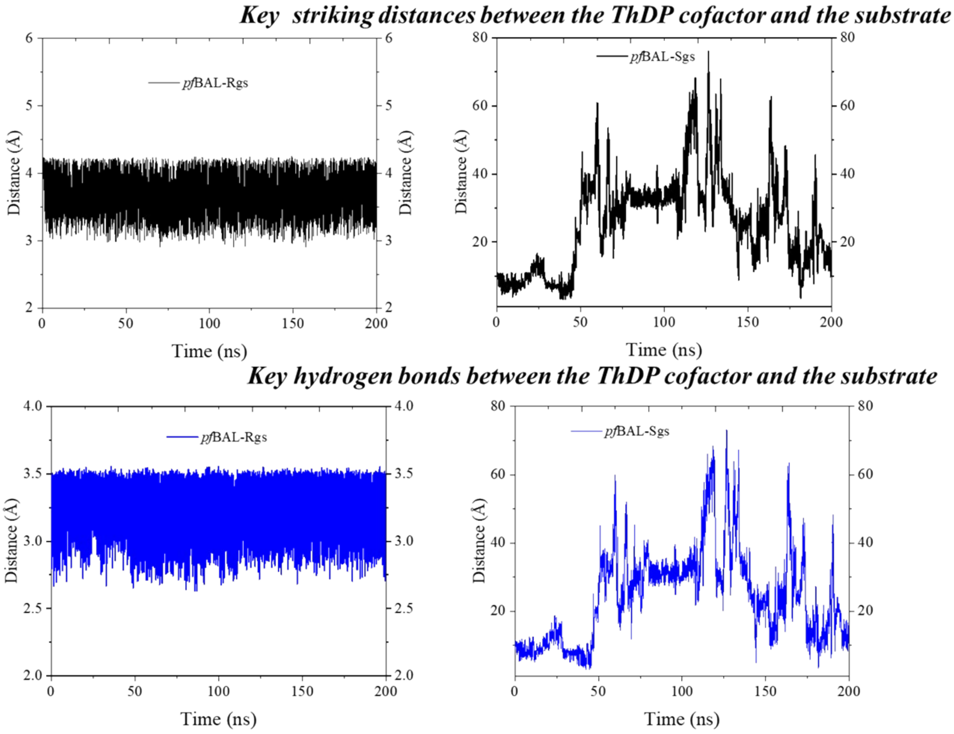

Figure S7 Monitoring the key distances change between the ThDP cofactor and the substrate in the MD simulation trajectories of *pf*BAL-Rgs, *pf*BAL-Sgs

Characterization data and calibration curves

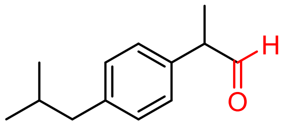

3b

^{1}H NMR (DMSO-d$_6$, 400 MHz) δ 9.61 (d, *J* = 1.3 Hz, 1H), 7.15 (s, 4H), 3.73 (qd, *J* = 7.0, 1.3 Hz, 1H), 2.42 (d, *J* = 7.2 Hz, 2H), 1.85-1.78 (m, 1H), 1.31 (d, *J* = 7.1 Hz, 3H), 0.85 (d, *J* = 6.6 Hz, 6H).

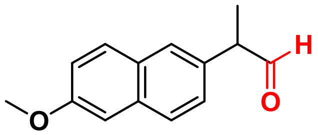

3c

^{1}H NMR (CDCl$_3$, 400 MHz) δ 9.74 (d, *J* = 1.4 Hz, 1H), 7.77-7.68 (m, 2H), 7.59 (d, *J* = 0.9 Hz, 1H), 7.30-7.25 (m, 1H), 7.19-7.14 (m, 1H), 7.13 (d, *J* = 2.4 Hz, 1H), 3.92 (s, 3H), 3.80-3.72 (m, 1H), 1.52 (d, *J* = 7.0 Hz, 3H).

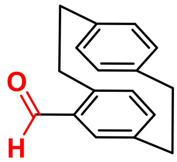

3d

^{1}H NMR (DMSO-d$_6$, 400 MHz) δ 9.94 (s, 1H), 7.03 (d, *J* = 1.7 Hz, 1H), 6.80 (dd, *J* = 7.7, 1.7 Hz, 1H), 6.65 (d, *J* = 7.7 Hz, 1H), 6.58 (dd, *J* = 7.8, 1.5 Hz, 1H), 6.51 (dd, *J* = 7.8, 1.6 Hz, 1H), 6.46 (s, 1H), 6.41 (dd, *J* = 7.8, 1.5 Hz, 1H), 6.26 (dd, *J* = 7.8, 1.7 Hz, 1H), 4.00 (dd, *J* = 18.5, 8.4 Hz, 1H), 3.23-2.87 (m, 9H).

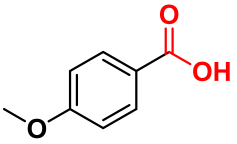

2a

^{1}H NMR (DMSO-d_6, 400 MHz) δ 12.63 (s, 1H), 7.93-7.85 (m, 2H), 7.04-6.98 (m, 2H), 3.82 (s, 3H). ^{13}C NMR (DMSO-d_6, 101 MHz) δ 167.05, 162.87, 131.39, 122.99, 113.84, 55.47.

Calibration curve of 2a:

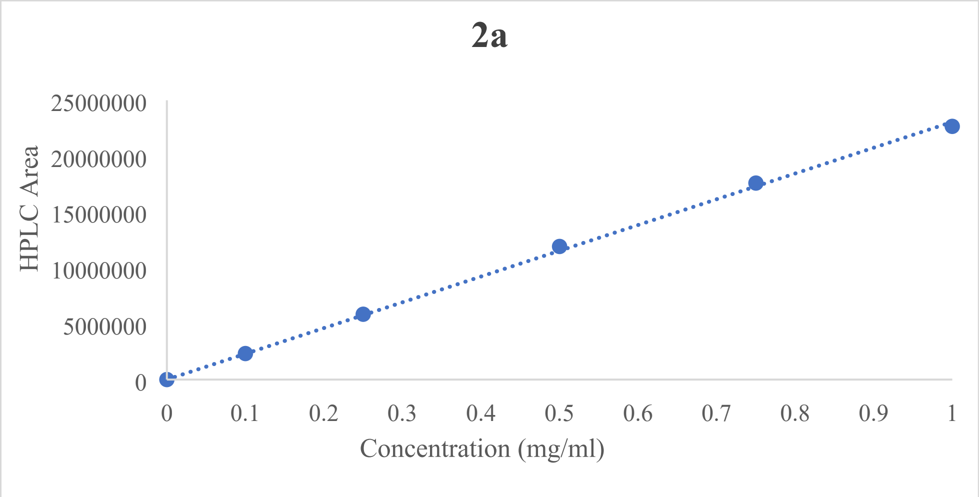

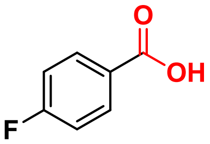

2b

^{1}H NMR (DMSO-d_6, 400 MHz) δ 13.05 (s, 1H), 8.0-7.94 (m, 2H), 7.30 (t, J = 8.8 Hz, 2H). ^{13}C NMR (DMSO-d_6, 101 MHz) δ 166.43, 164.96 (d, J = 251.5 Hz), 132.15 (d, J = 9.5 Hz), 127.39 (d, J = 2.8 Hz), 115.66 (d, J = 22.0 Hz).

Calibration curve of 2b:

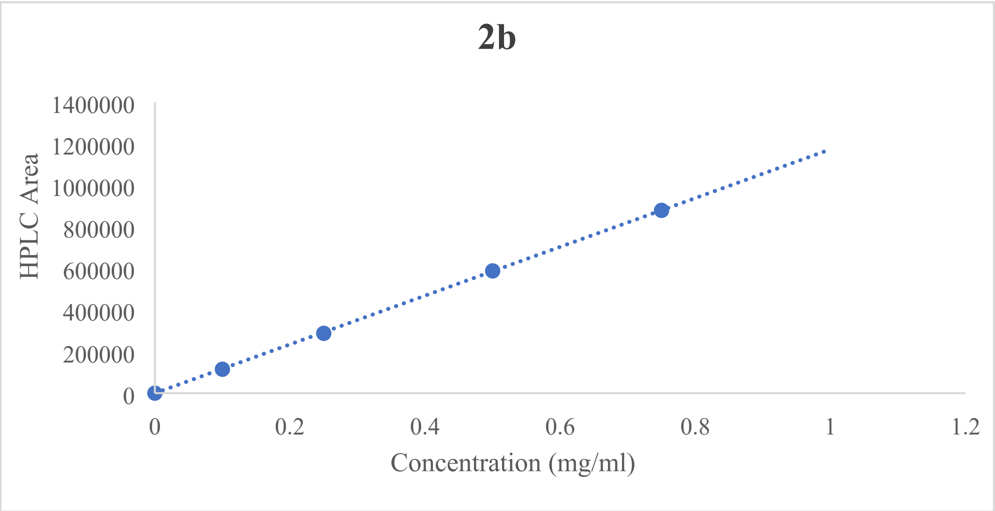

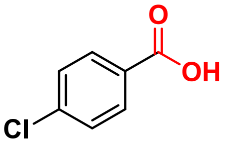

2c

^{1}H NMR (DMSO-d_6, 400 MHz) δ 13.18 (s, 1H), 7.98-7.88 (m, 2H), 7.61-7.50 (m, 2H). ^{13}C NMR (DMSO-d_6, 101 MHz) δ 166.50, 137.84, 131.18, 129.66, 128.78.

Calibration curve of 2c:

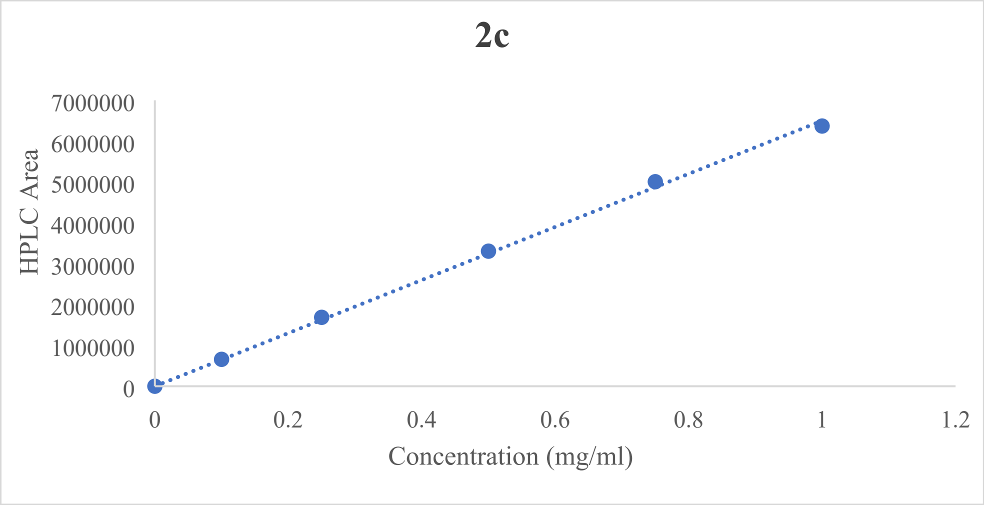

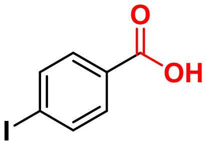

2d

^{1}H NMR (DMSO-d_6, 400 MHz) δ 13.19 (s, 1H), 7.89-7.83 (m, 2H), 7.73-7.67 (m, 2H). ^{13}C NMR (DMSO-d_6, 101 MHz) δ 166.95, 137.61, 131.10, 130.29, 101.24.

Calibration curve of 2d:

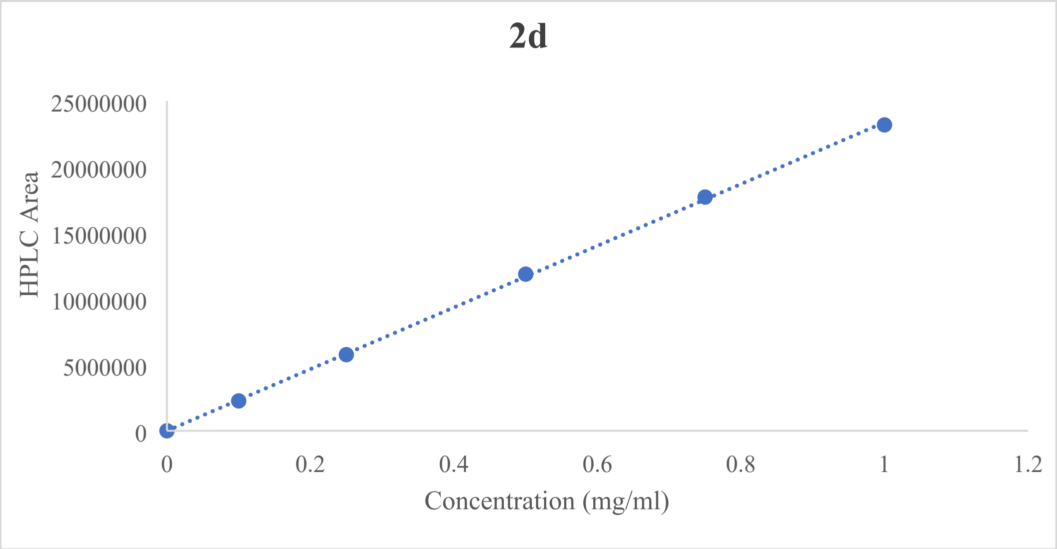

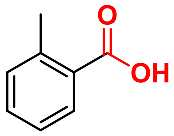

2e

^{1}H NMR (DMSO-d$_6$, 400 MHz) δ 12.81 (s, 1H), 7.81 (d, *J* = 7.6 Hz, 1H), 7.47-7.39 (m, 1H), 7.31-7.24 (m, 2H), 2.51 (s, 3H). ^{13}C NMR (DMSO-d$_6$, 101 MHz) δ 168.71, 139.03, 131.74, 131.53, 130.46, 130.21, 125.86, 21.29.

Calibration curve of 2e:

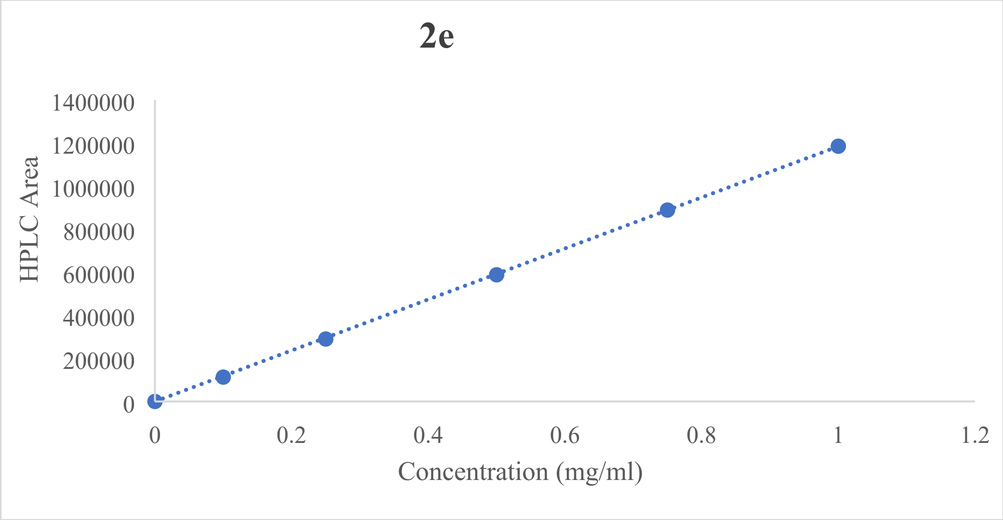

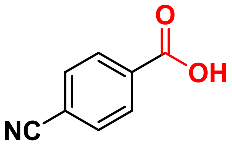

2f

^{1}H NMR (DMSO-d$_6$, 400 MHz) δ 13.57 (s, 1H), 8.10-8.04 (m, 2H), 8.00-7.94 (m, 2H). ^{13}C NMR (DMSO-d$_6$, 101 MHz) δ 166.10, 134.86, 132.72, 129.96, 118.23, 115.11.

Calibration curve of 2f:

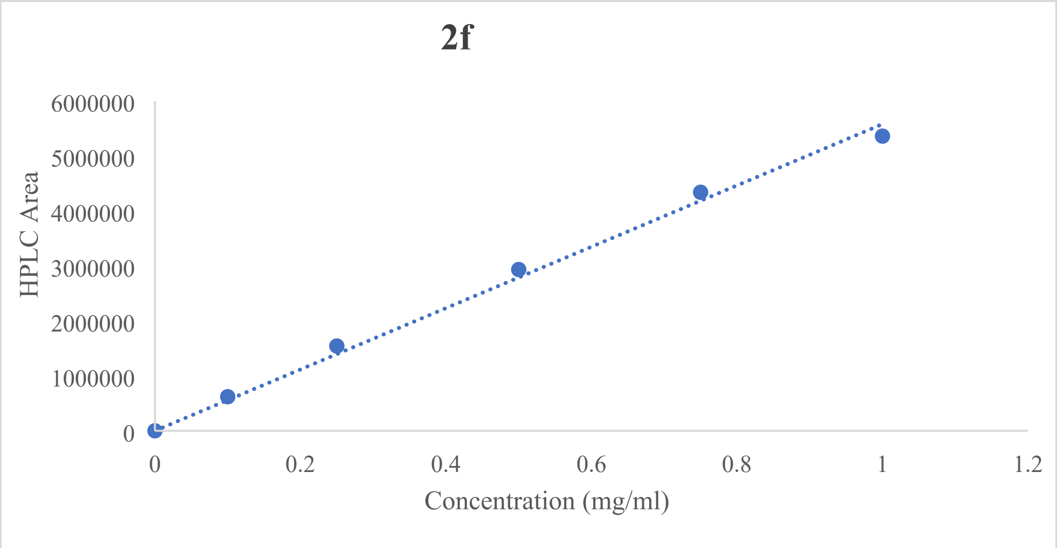

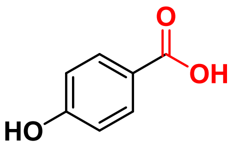

2g

^{1}H NMR (DMSO-d_6, 400 MHz) δ 12.42 (s, 1H), 10.23 (s, 1H), 7.87-7.73 (m, 2H), 6.88-6.76 (m, 2H). ^{13}C NMR (DMSO-d_6, 101 MHz) δ 167.25, 161.67, 131.61, 121.41, 115.19.

Calibration curve of 2g:

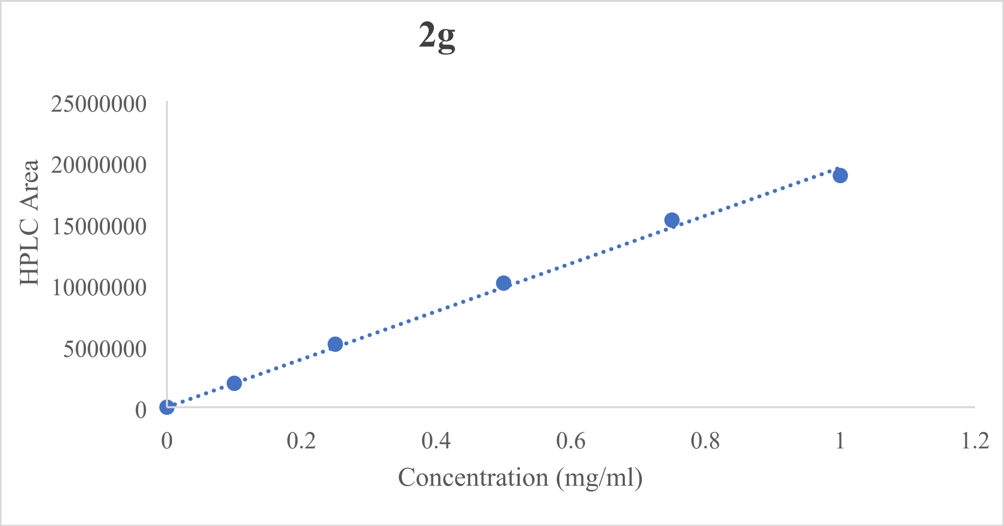

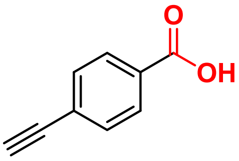

2h

^{1}H NMR (DMSO-d_6, 400 MHz) δ 13.15 (s, 1H), 7.96-7.90 (m, 2H), 7.62-7.56 (m, 2H), 4.43 (s, 1H).^{13}C NMR (DMSO-d_6, 101 MHz) δ 166.69, 131.94, 130.89, 129.53, 126.06, 83.63, 82.79.

Calibration curve of 2h:

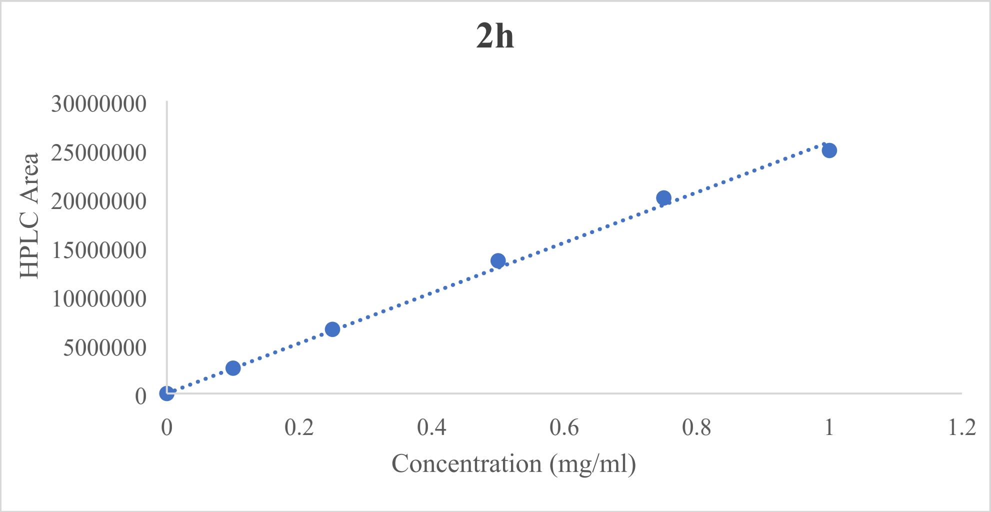

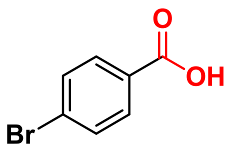

2i

^{1}H NMR (DMSO-d_6, 400 MHz) δ 13.19 (s, 1H), 7.89-7.83 (m, 2H), 7.73-7.67 (m, 2H). ^{13}C NMR (DMSO-d_6, 101 MHz) δ 166.65, 131.74, 131.33, 130.01, 126.93.

Calibration curve of 2i:

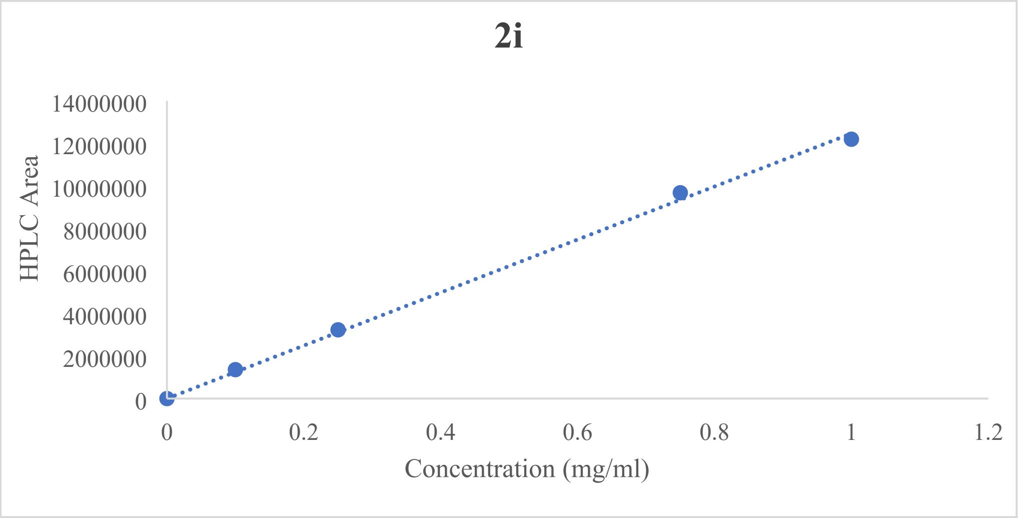

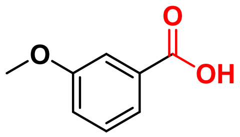

2j

^{1}H NMR (DMSO-d_6, 400 MHz) δ 13.00 (s, 1H), 7.57-7.49 (m, 1H), 7.47-7.38 (m, 2H), 7.22-7.13 (m, 1H), 3.80 (s, 3H). ^{13}C NMR (DMSO-d_6, 101 MHz) δ 167.16, 159.24, 132.20, 129.74, 121.57, 118.92, 113.90, 55.27.

Calibration curve of 2j:

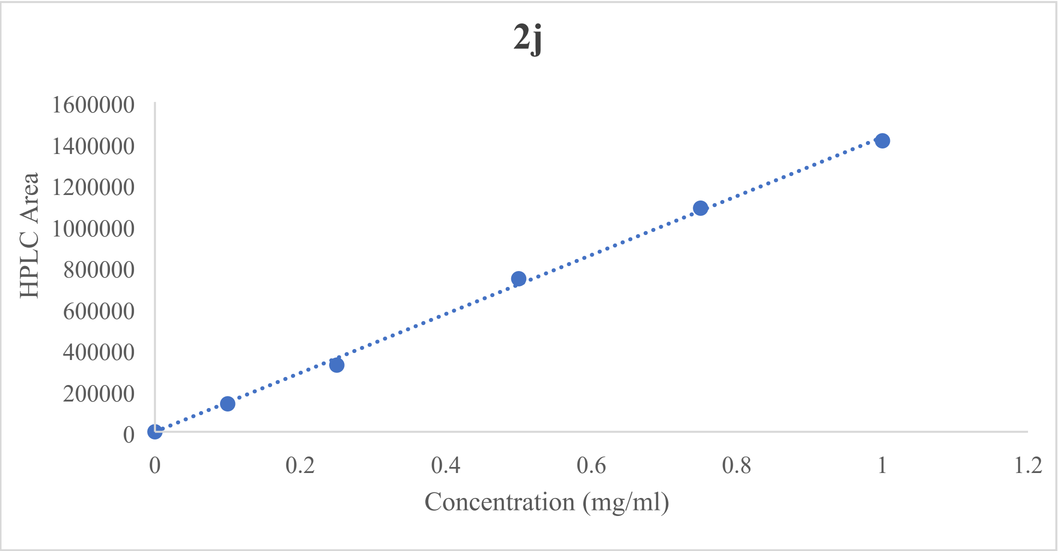

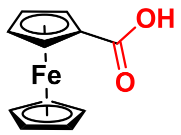

2k

^{1}H NMR (DMSO-d$_6$, 400 MHz) δ 12.13 (s, 1H), 4.79 (d, *J* = 23.6 Hz, 2H), 4.55 (d, *J* = 36.8 Hz, 2H), 4.26 (s, 5H). ^{13}C NMR (DMSO-d$_6$, 101 MHz) δ 171.98, 70.86, 69.71, 69.44, 69.31.

Calibration curve of 2k:

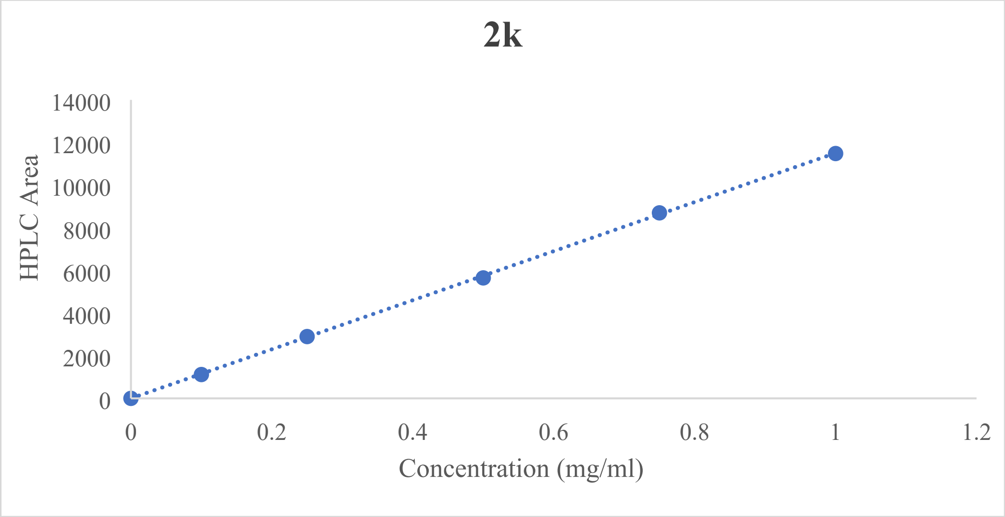

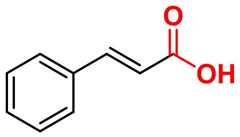

2l

^{1}H NMR (DMSO-d$_6$, 400 MHz) δ 12.42 (s, 1H), 7.72-7.64 (m, 2H), 7.59 (d, *J* = 16.0 Hz, 1H), 7.48-7.34 (m, 3H), 6.53 (d, *J* = 16.0 Hz, 1H). ^{13}C NMR (101 MHz, DMSO-d$_6$) δ 167.60, 143.95, 134.24, 130.24, 128.92, 128.22, 119.24.

Calibration curve of 2l:

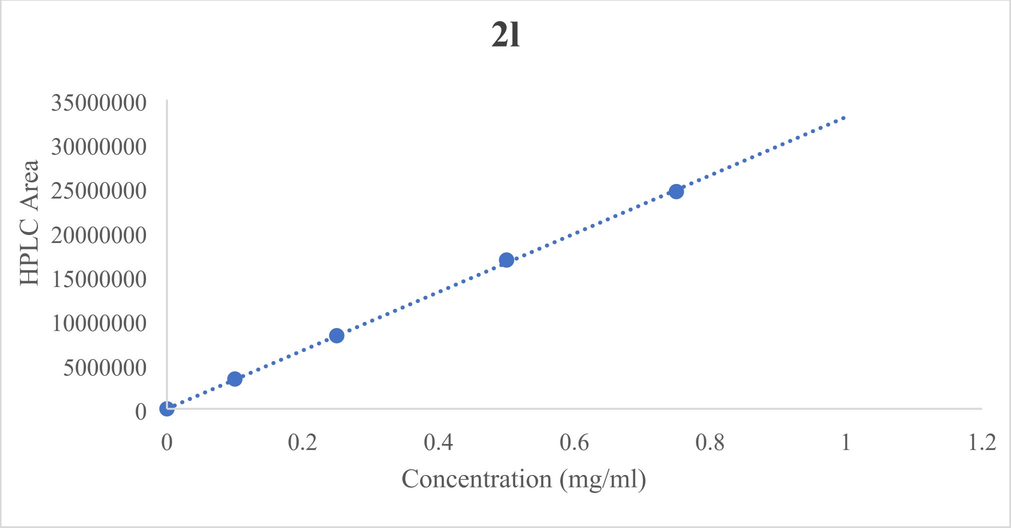

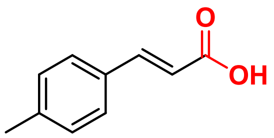

2m

^{1}H NMR (DMSO-d$_6$, 400 MHz) δ 12.33 (s, 1H), 7.64-7.48 (m, 3H), 7.22 (d, *J* = 8.0 Hz, 2H), 6.46 (d, *J* = 16.0 Hz, 1H), 2.32 (s, 3H). ^{13}C NMR (DMSO-d$_6$, 101 MHz) δ 167.71, 143.95, 140.16, 131.51, 129.53, 128.21, 118.10, 21.03.

Calibration curve of 2m:

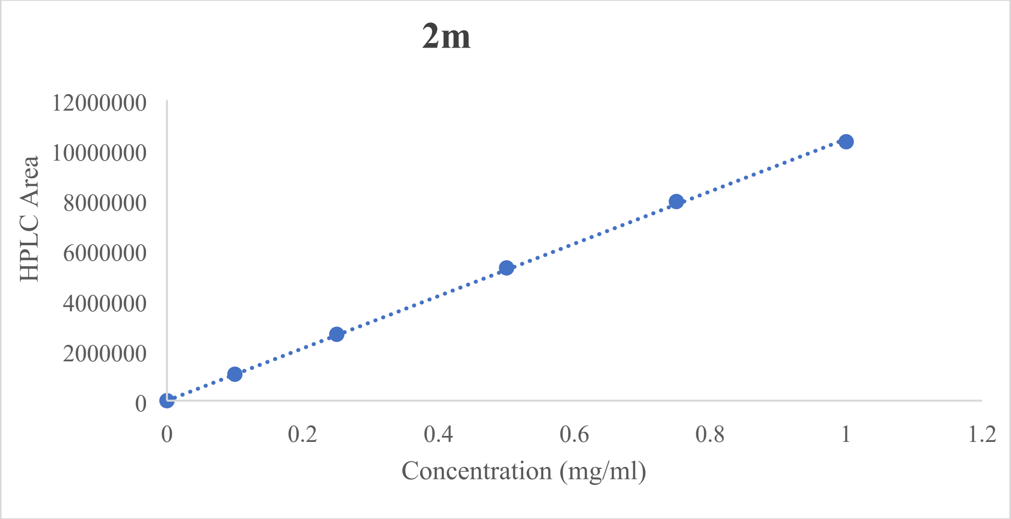

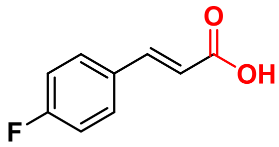

2n

^{1}H NMR (DMSO-d$_6$, 400 MHz) δ 12.41 (s, 1H), 7.80-7.72 (m, 2H), 7.59 (d, *J* = 16.0 Hz, 1H), 7.31-7.19 (m, 2H), 6.49 (d, *J* = 16.0 Hz, 1H). ^{13}C NMR (DMSO-d$_6$, 101 MHz) δ 167.57, 163.18 (d, *J* = 249.2 Hz), 142.75, 130.93 (d, *J* = 3.1 Hz), 130.55 (d, *J* = 8.6 Hz), 119.14 (d, *J* = 2.2 Hz), 115.92 (d, *J* = 21.8 Hz).

Calibration curve of 2n:

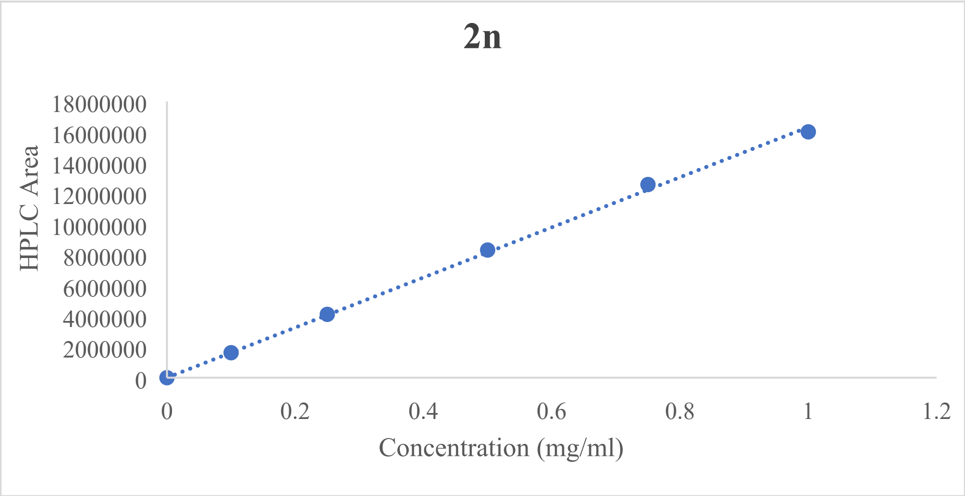

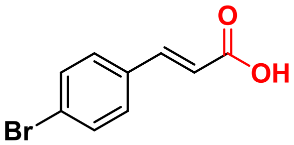

2o

^{1}H NMR (DMSO-d_6, 400 MHz) δ 12.49 (s, 1H), 7.67-7.53 (m, 5H), 6.56 (d, J = 16.0 Hz, 1H). ^{13}C NMR (DMSO-d_6, 101 MHz) δ 167.45, 142.64, 133.56, 131.88, 130.19, 123.56, 120.15.

Calibration curve of 2o:

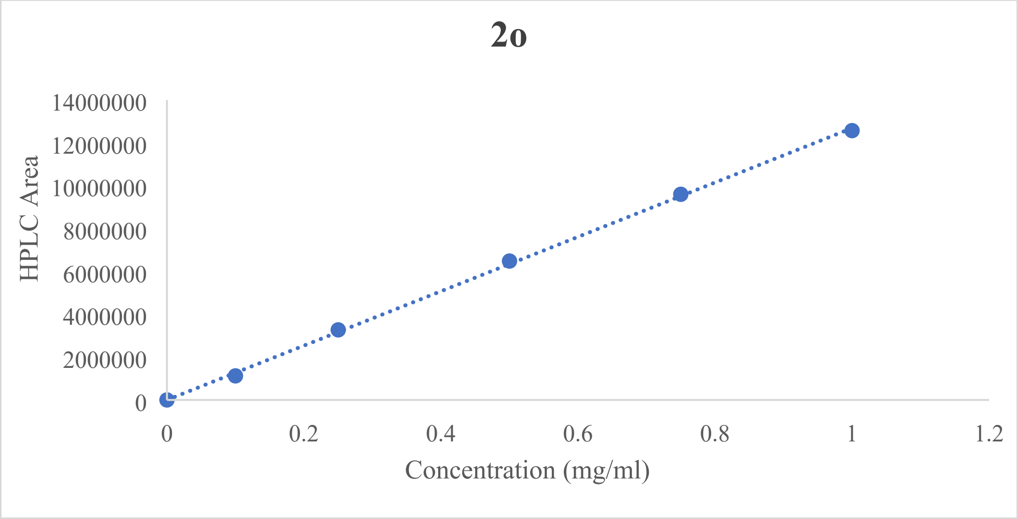

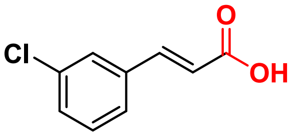

2p

^{1}H NMR (DMSO-d_6, 400 MHz) δ 12.52 (s, 1H), 7.80 (s, 1H), 7.70-7.62(m, 1H), 7.57 (d, J = 16.0 Hz, 1H), 7.50-7.37 (m, 2H), 6.60 (d, J = 16.0 Hz, 1H). ^{13}C NMR (DMSO-d_6, 101 MHz) δ 167.39, 142.35, 136.54, 133.75, 130.70, 129.85, 127.85, 126.82, 121.00.

Calibration curve of 2p:

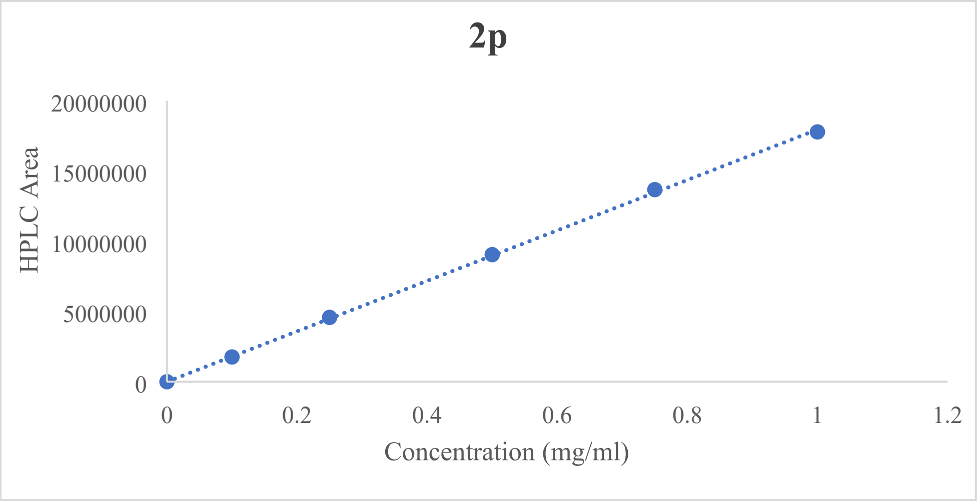

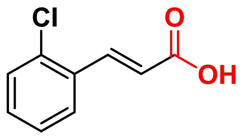

2q

^{1}H NMR (DMSO-d_6, 400 MHz) δ 12.67 (s, 1H), 7.95-7.90 (m, 1H), 7.87 (d, J = 16.0 Hz, 1H), 7.58-7.50 (m, 1H), 7.46-7.36 (m, 2H), 6.60 (d, J = 16 Hz, 1H).. ^{13}C NMR (DMSO-d_6, 101 MHz) δ 167.21, 138.73, 133.60, 131.80 , 129.98, 128.27, 127.81, 122.33.

Calibration curve of 2q:

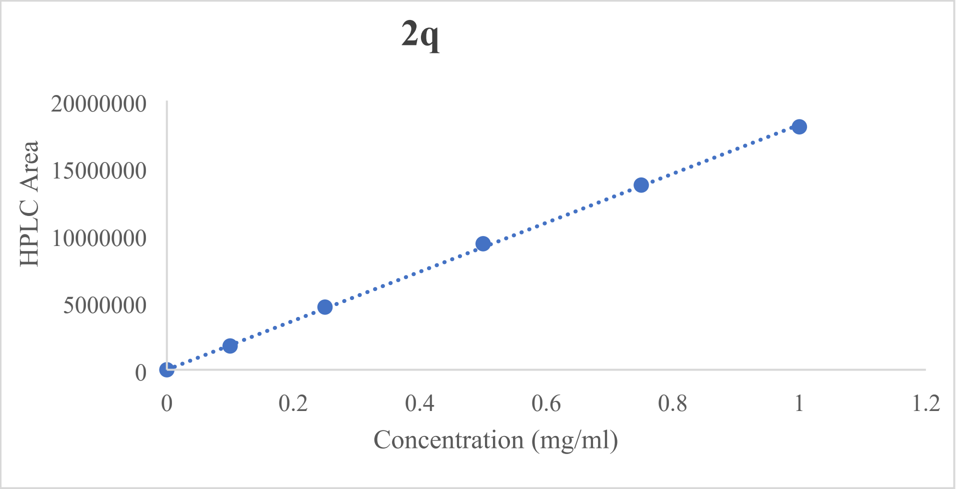

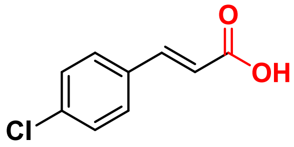

2r

^{1}H NMR (DMSO-d_6, 400 MHz) δ 12.48 (s, 1H), 7.72 (d, J = 8.8 Hz, 2H), 7.58 (d, J = 16.0 Hz, 1H), 7.47 (d, J = 8.4 Hz, 2H), 6.55 (d, J = 16.0 Hz, 1H). ^{13}C NMR (DMSO-d_6, 101 MHz) δ 167.46, 142.55, 134.73, 133.23, 129.97, 128.95, 120.09.

Calibration curve of 2r:

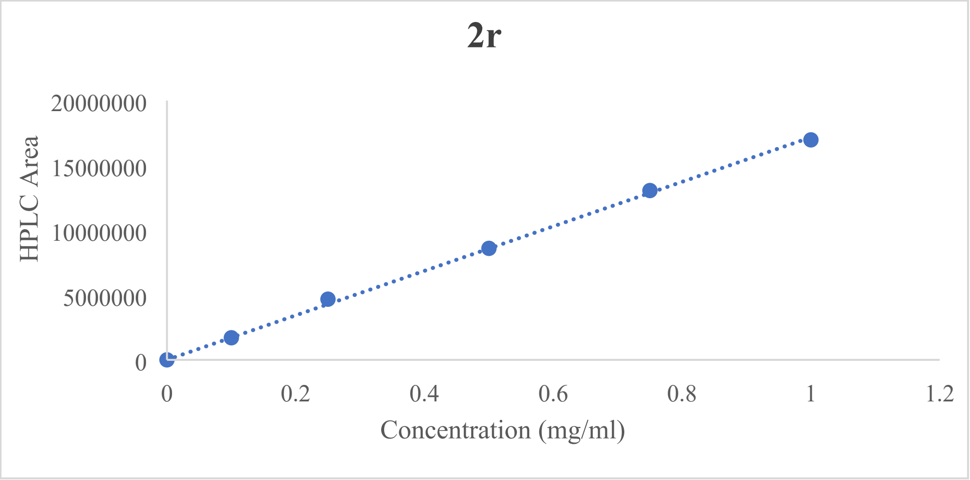

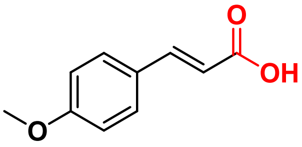

2s

^{1}H NMR (DMSO-d_6, 400 MHz) δ 12.23 (s, 1H), 7.67-7.59 (m, 2H), 7.54 (d, *J* = 16.0 Hz, 1H), 7.00-6.92 (m, 2H), 6.37 (d, *J* = 16.0 Hz, 1H), 3.79 (s, 3H). ^{13}C NMR (DMSO-d_6, 101 MHz) δ 167.88, 160.97, 143.79, 129.98, 126.85, 116.52, 114.38, 55.34.

Calibration curve of 2s:

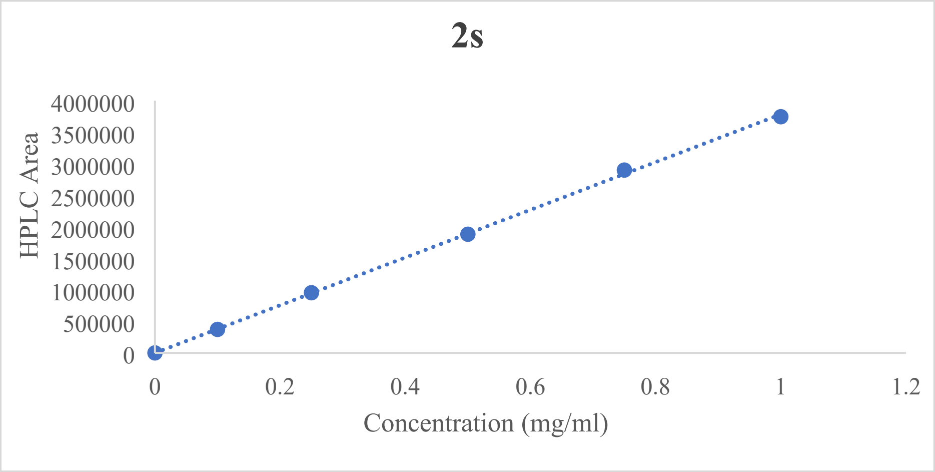

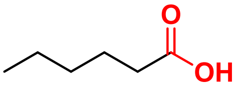

2t

^{1}H NMR ($CDCl_3$, 400 MHz) δ 2.35 (t, *J* = 7.6 Hz, 2H), 1.70-1.58 (m, 2H), 1.39-1.27 (m, 4H), 0.90 (t, *J* = 6.8 Hz, 3H). ^{13}C NMR ($CDCl_3$, 101 MHz) δ 180.58, 34.21, 31.34, 24.50, 22.44, 14.01.

Calibration curve of 2t:

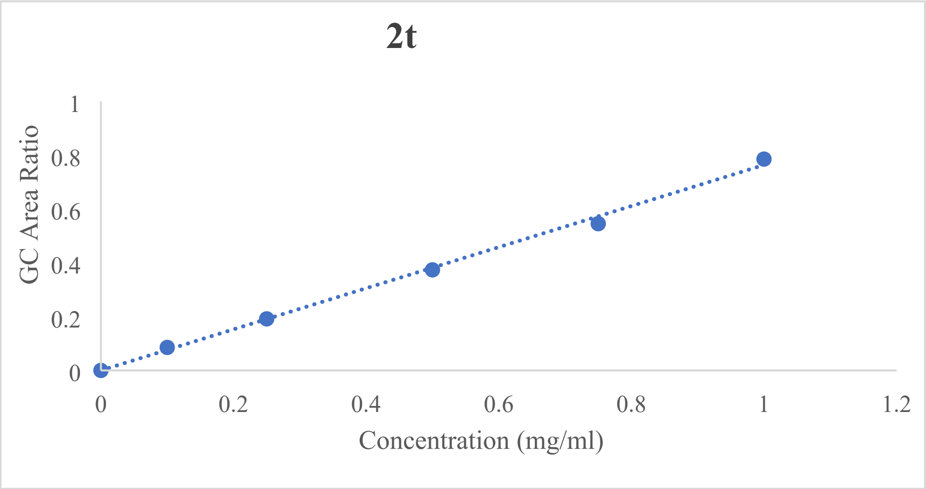

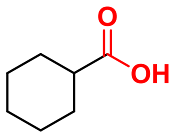

2u

^{1}H NMR ($CDCl_3$, 400 MHz) δ 10.44 (s, 1H), 2.40-2.26 (m, 1H), 1.99-1.88 (m, 2H), 1.82-1.71 (m, 2H), 1.69-1.61 (m, 1H), 1.52-1.39 (m, 2H), 1.35-1.20 (m, 3H). ^{13}C NMR ($CDCl_3$, 101 MHz) δ 182.77, 43.06, 28.90, 25.82, 25.47.

Calibration curve of 2u:

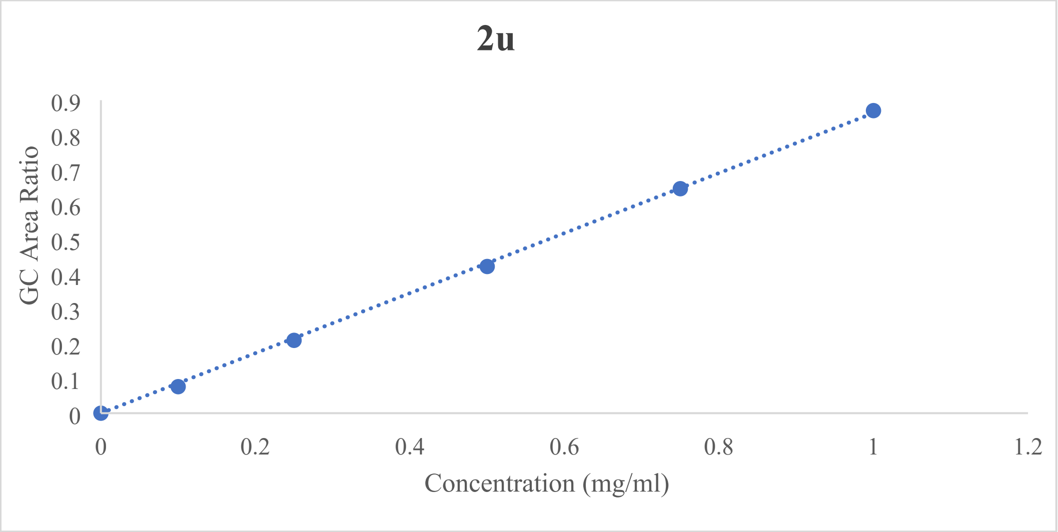

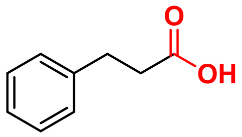

2v

^{1}H NMR (DMSO-d_6, 400 MHz) δ 12.13 (s, 1H), 7.31-7.15 (m, 5H), 2.82 (t, J = 7.6 Hz, 2H), 2.53 (t, J = 7.6 Hz, 2H). ^{13}C NMR (DMSO-d_6, 101 MHz) δ 173.78, 140.89, 128.28(d, J = 6.2 Hz), 125.98, 35.26, 30.36.

Calibration curve of 2v:

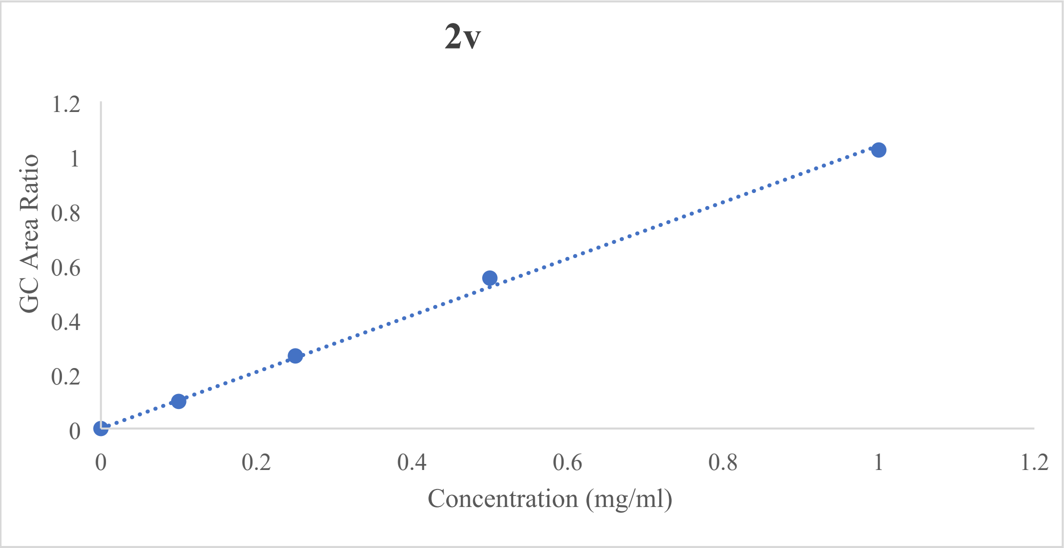

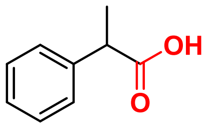

4a

^{1}H NMR ($CDCl_3$, 400 MHz) δ 10.35 (s, 1H), 7.36-7.24 (m, 5H), 3.73 (q, *J* = 7.2 Hz, 1H), 1.51 (d, *J* = 7.2 Hz, 3H). ^{13}C NMR ($CDCl_3$, 101 MHz) δ 180.73, 139.89, 128.82, 127.73, 127.53, 45.47, 18.24.

Calibration curve of 4a:

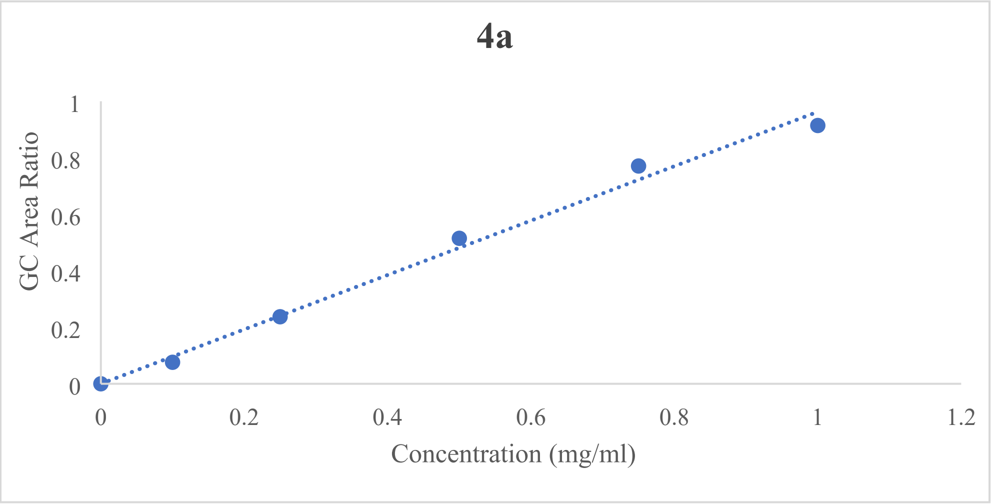

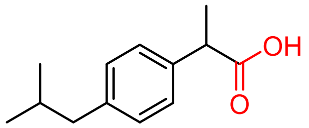

4b

^{1}H NMR (DMSO-d_6, 400 MHz) δ 12.24 (s, 1H), 7.18 (d, *J* = 8.0 Hz, 2H), 7.09 (d, *J* = 8.0 Hz, 2H), 3.62 (q, *J* = 7.2 Hz, 1H), 2.41 (d, *J* = 7.2 Hz, 2H), 1.88-1.72 (m, 1H), 1.34 (d, *J* = 6.8 Hz, 3H), 0.85 (d, *J* = 6.4 Hz, 6H). ^{13}C NMR (DMSO-d_6, 101 MHz) δ 175.49, 139.56, 138.50, 128.97, 127.12, 44.27, 29.64, 22.20, 18.56.

Calibration curve of 4b:

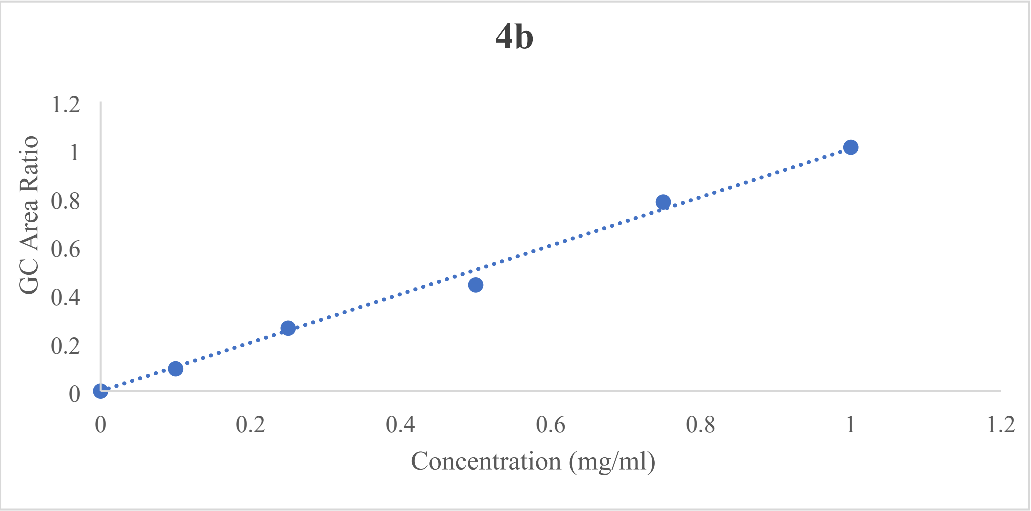

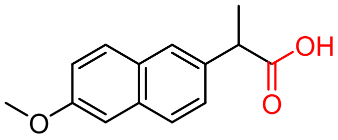

4c

^{1}H NMR (DMSO-d_6, 400 MHz) δ 12.31 (s, 1H), 7.78 (t, *J* = 9.3 Hz, 2H), 7.71 (s, 1H), 7.40 (dd, *J* = 8.4, 1.7 Hz, 1H), 7.28 (d, *J* = 2.4 Hz, 1H), 7.15 (dd, *J* = 8.9, 2.6 Hz, 1H), 3.86 (s, 3H), 3.80 (q, *J* = 7.1 Hz, 1H), 1.44 (d, *J* = 7.1 Hz, 3H). ^{13}C NMR (DMSO-d_6, 101 MHz) δ 175.48, 157.12, 136.33, 133.25, 129.12, 128.42, 126.85, 126.41, 125.57, 118.69, 105.72, 55.17, 44.60, 18.46.

Calibration curve of 4c:

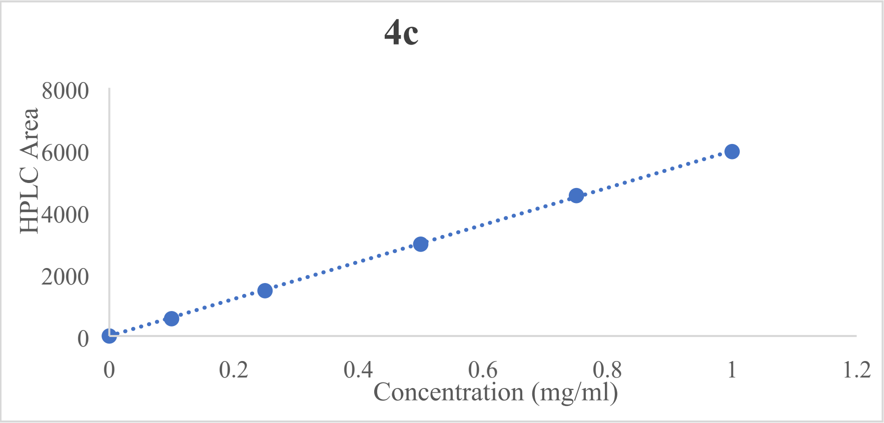

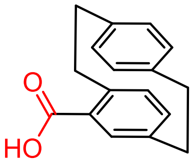

4d

^{1}H NMR (DMSO-d_6, 400 MHz) δ 12.52 (s, 1H), 7.07 (d, *J* = 2 Hz, 1H), 6.69 (dd, *J* = 8, 2 Hz, 1H), 6.56 (d, *J* = 7.6 Hz, 2H), 6.50 (d, *J* = 7.6 Hz, 1H), 6.42 (d, *J* = 8.4 Hz, 2H), 4.00 (t, *J* = 10.8 Hz, 1H), 3.15-3.05 (m, 3H), 3.03-2.90 (m, 3H), 2.86 – 2.77 (m, 1H). ^{13}C NMR (DMSO-d_6, 101 MHz) δ 167.99, 142.02, 139.59, 139.30, 136.06, 135.24, 133.16, 132.69, 132.02, 131.16, 35.46.

Calibration curve of 4d:

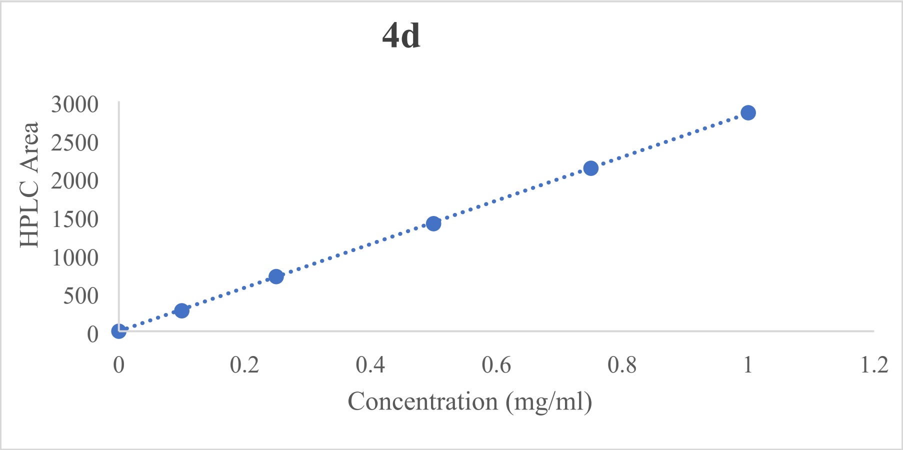

NMR Spectra

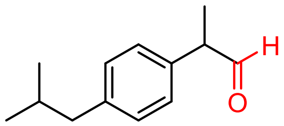

3b

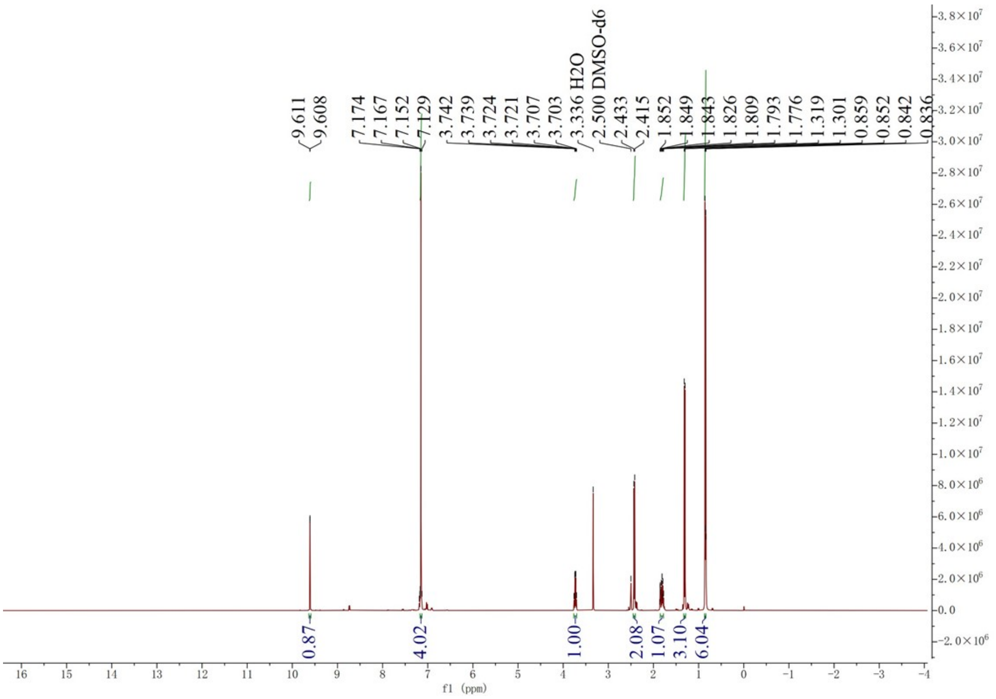

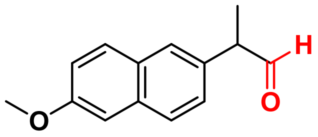

3c

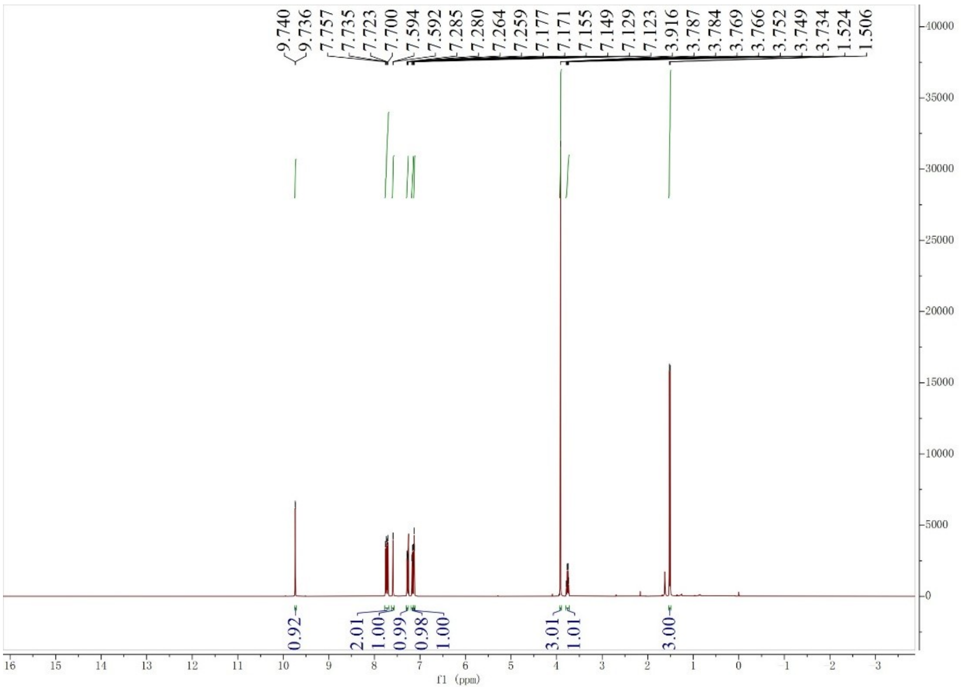

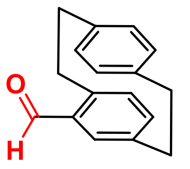
O
H

3d

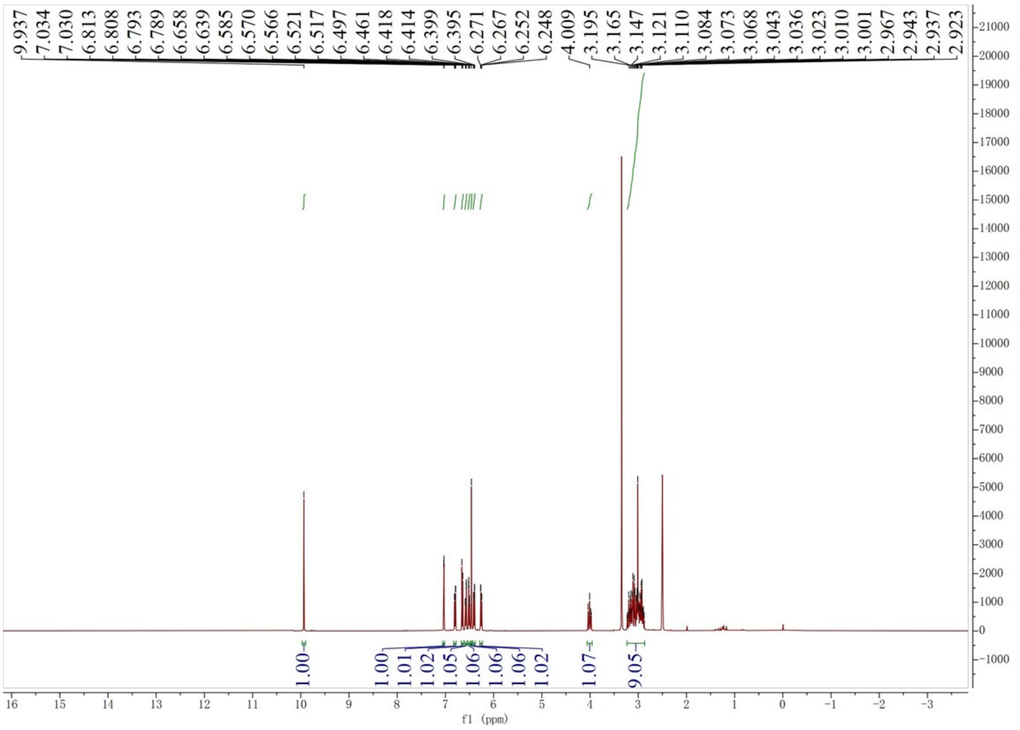
9.937
7.034
7.030
6.813
6.808
6.793
6.789
6.658
6.639
6.585
6.570
6.566
6.521
6.517
6.497
6.461
6.418
6.414
6.399
6.395
6.271
6.267
6.252
6.248
4.009
3.195
3.165
3.147
3.121
3.110
3.084
3.073
3.068
3.043
3.036
3.023
3.010
3.001
2.967
2.943
2.937
2.923
1.00
1.00
1.01
1.02
1.05
1.06
1.06
1.06
1.02
1.07
9.05
f1 (ppm)

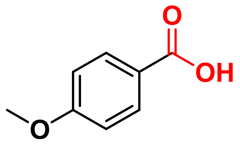
O
OH
O

2a

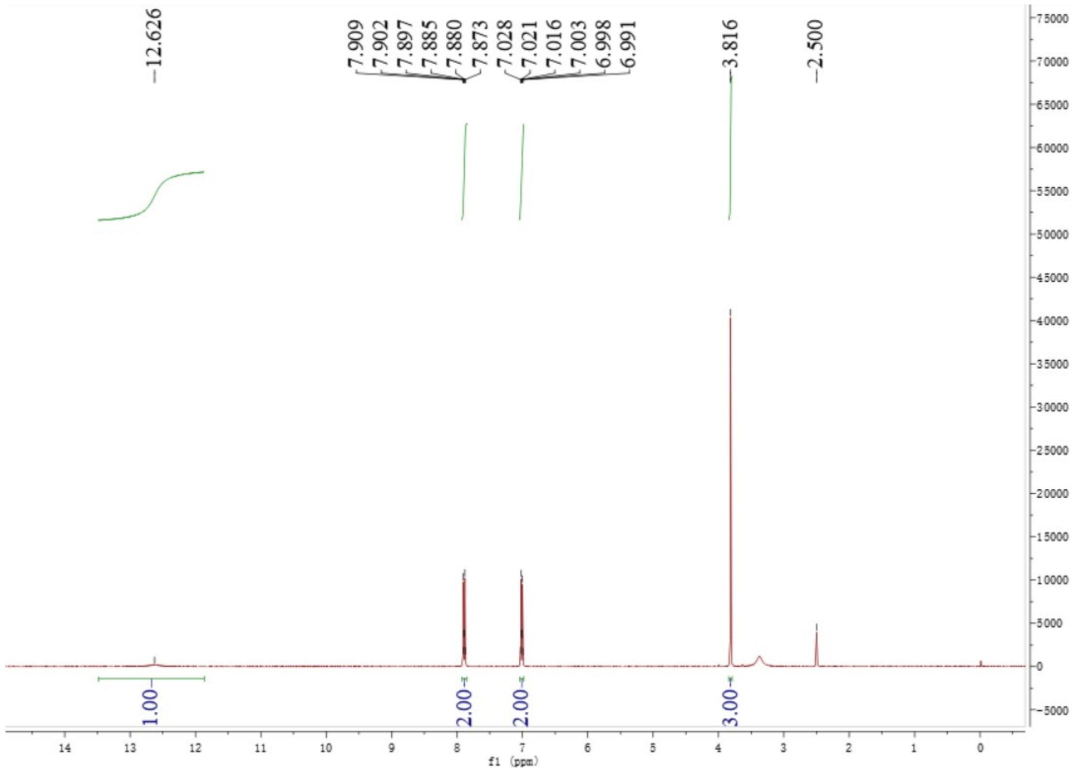
12.626
7.909
7.902
7.897
7.885
7.880
7.873
7.028
7.021
7.016
7.003
6.998
6.991
3.816
2.500
1.00
2.00
2.00
3.00
f1 (ppm)

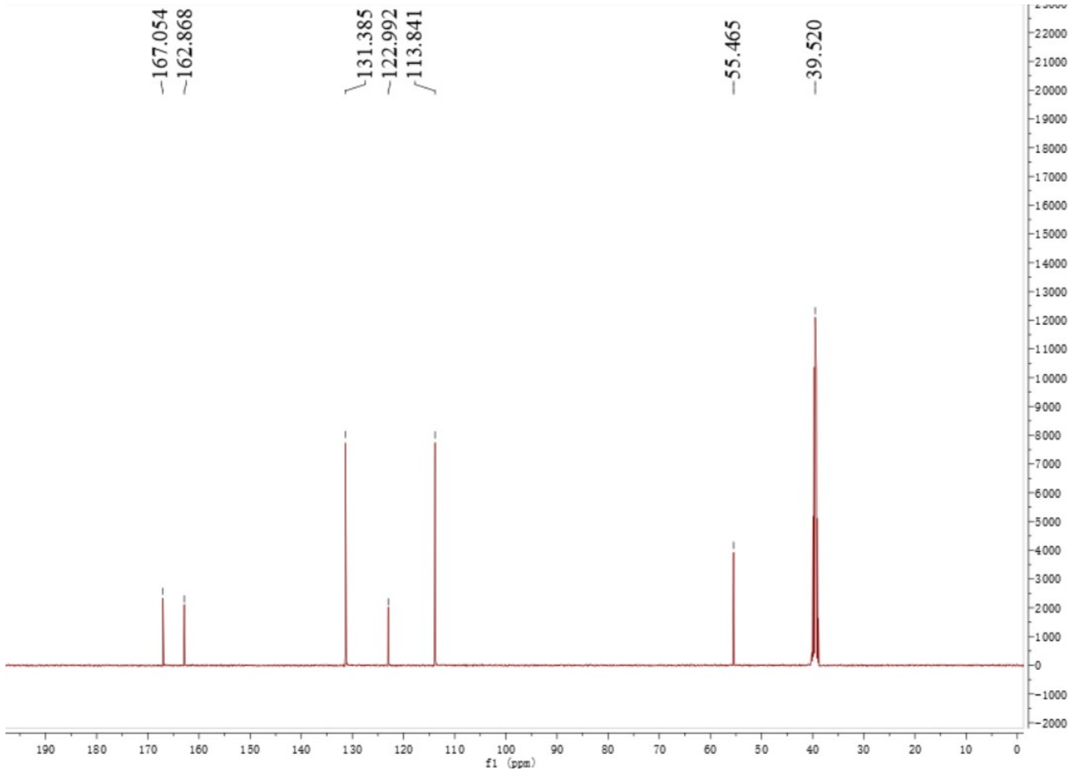
167.054
162.868
131.385
122.992
113.841
55.465
39.520
f1 (ppm)

^{1}H NMR spectrum of **2a** from the enzymatic reaction:

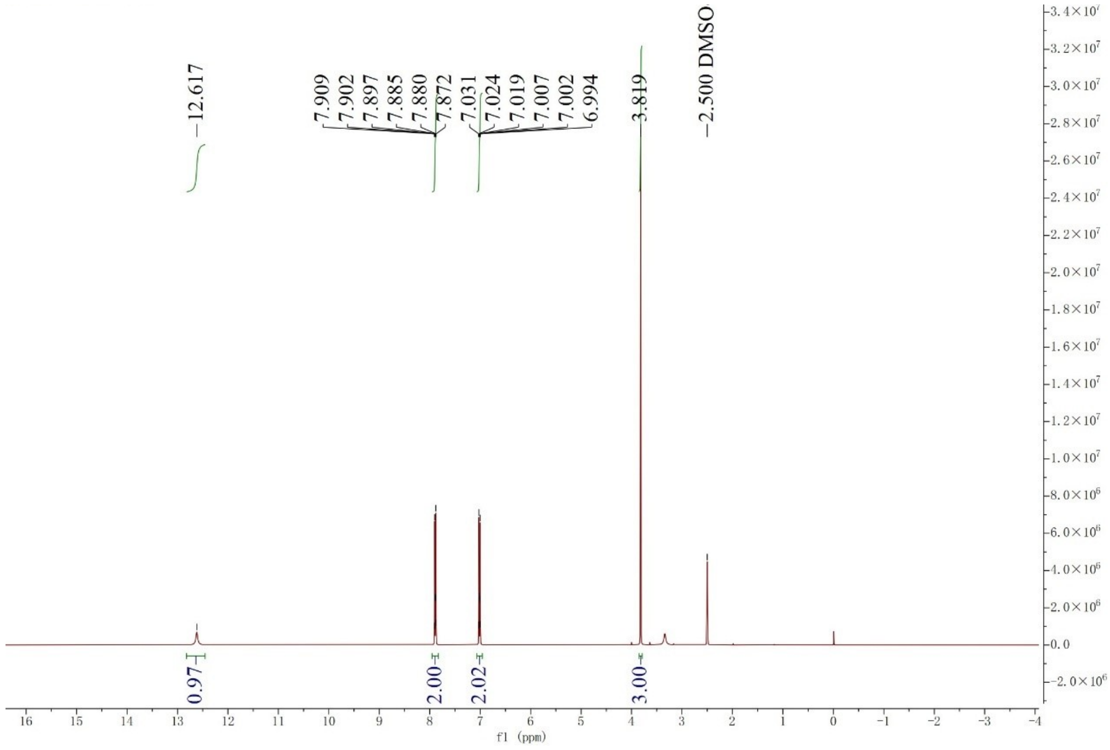

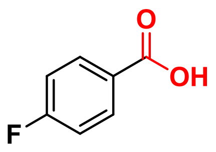
O
OH
F

2b

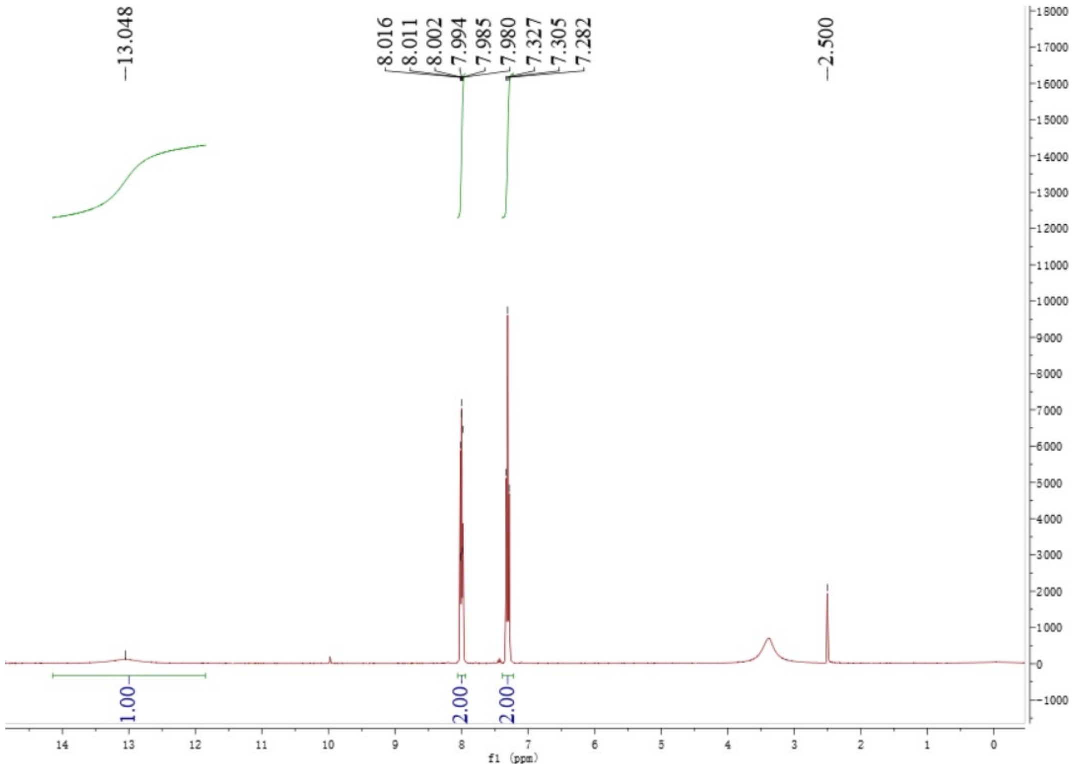
13.048
8.016
8.011
8.002
7.994
7.985
7.980
7.327
7.305
7.282
2.500
1.00
2.00
2.00
f1 (ppm)

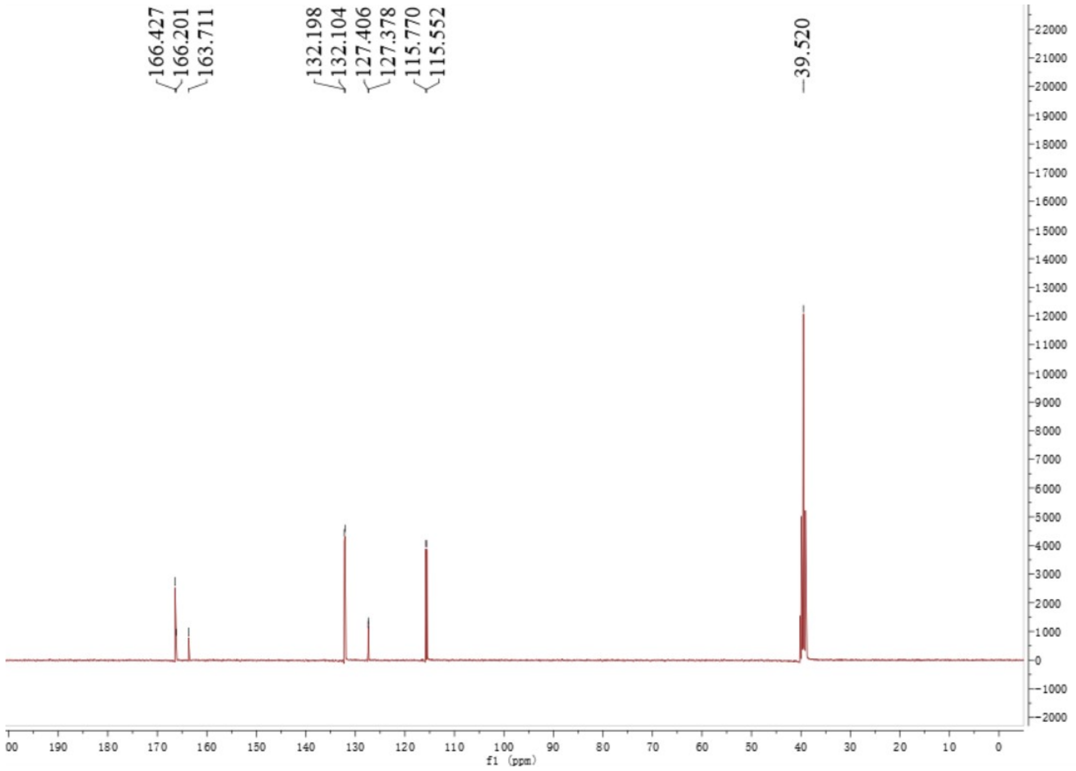
166.427
166.201
163.711
132.198
132.104
127.406
127.378
115.770
115.552
39.520
f1 (ppm)

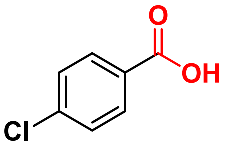

2c

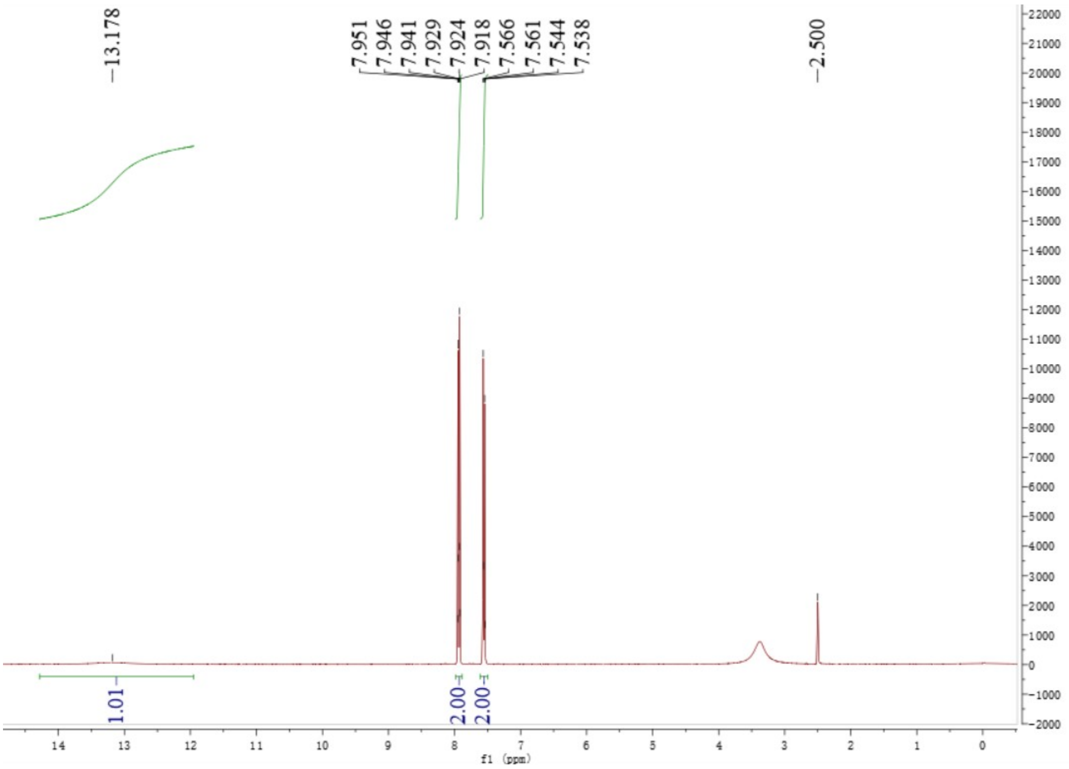
13.178
7.951
7.946
7.941
7.929
7.924
7.918
7.566
7.561
7.544
7.538
2.500
1.01
2.00
2.00
f1 (ppm)

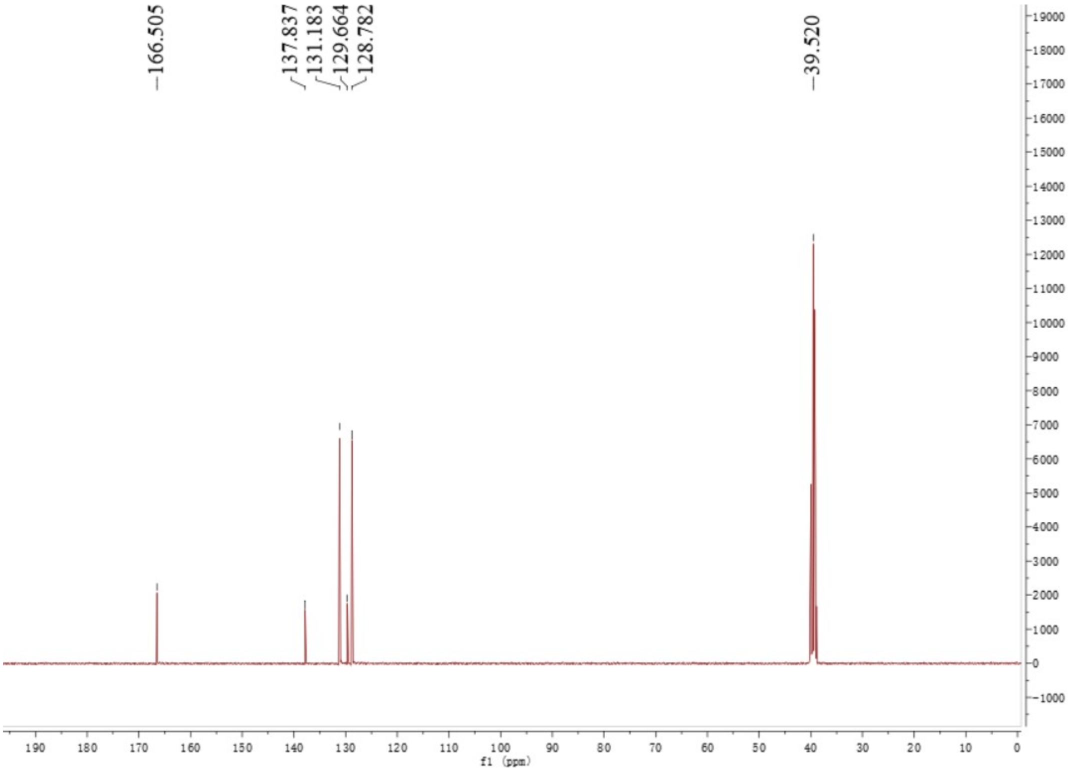
166.505
137.837
131.183
129.664
128.782
39.520
f1 (ppm)

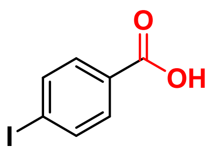
O
OH
I

2d

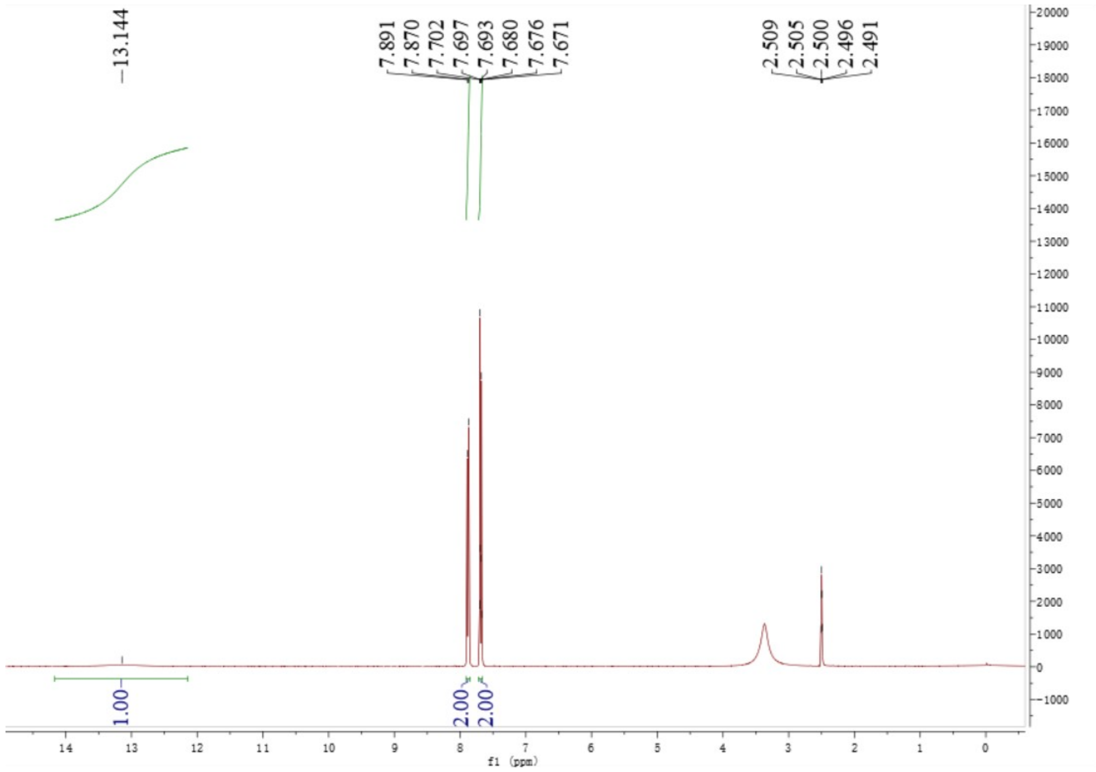
13.144
7.891
7.870
7.702
7.697
7.693
7.680
7.676
7.671
2.509
2.505
2.500
2.496
2.491
1.00
2.00
2.00
f1 (ppm)

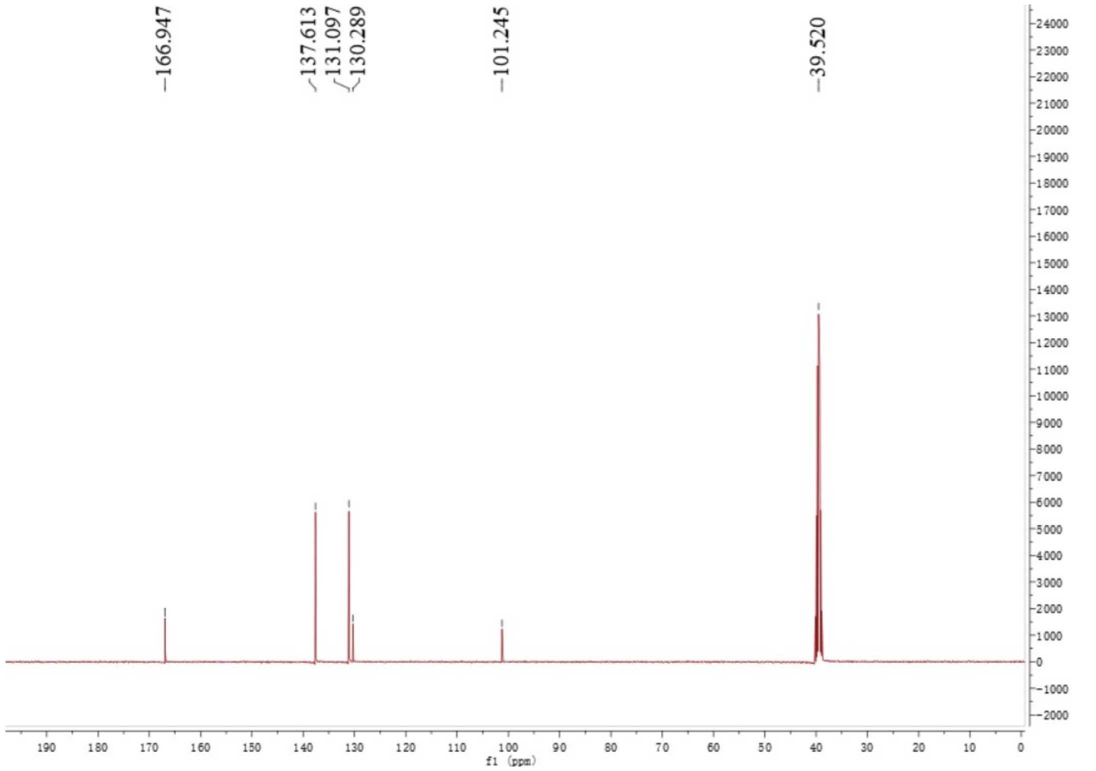
166.947
137.613
131.097
130.289
101.245
39.520
f1 (ppm)

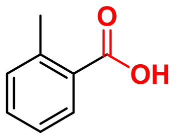
O
OH

2e

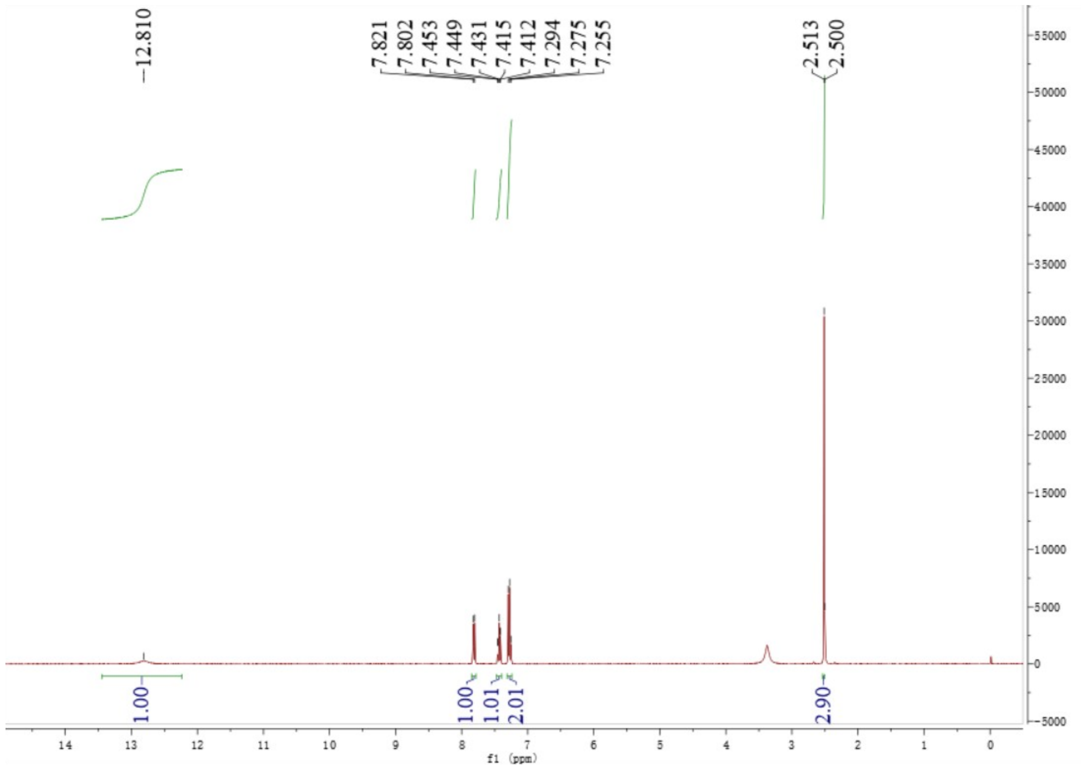
12.810
7.821
7.802
7.453
7.449
7.431
7.415
7.412
7.294
7.275
7.255
2.513
2.500
1.00
1.00
1.01
2.01
2.90
f1 (ppm)

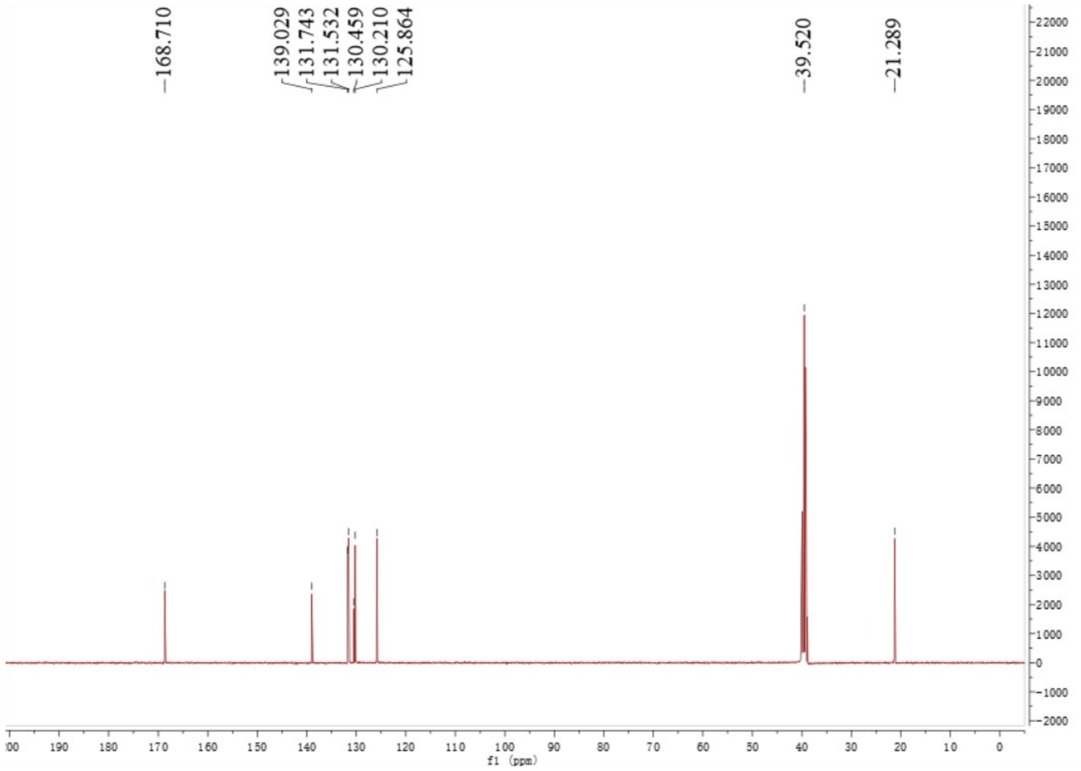
168.710
139.029
131.743
131.532
130.459
130.210
125.864
39.520
21.289
f1 (ppm)

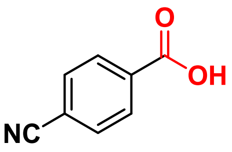
O
OH
NC

2f

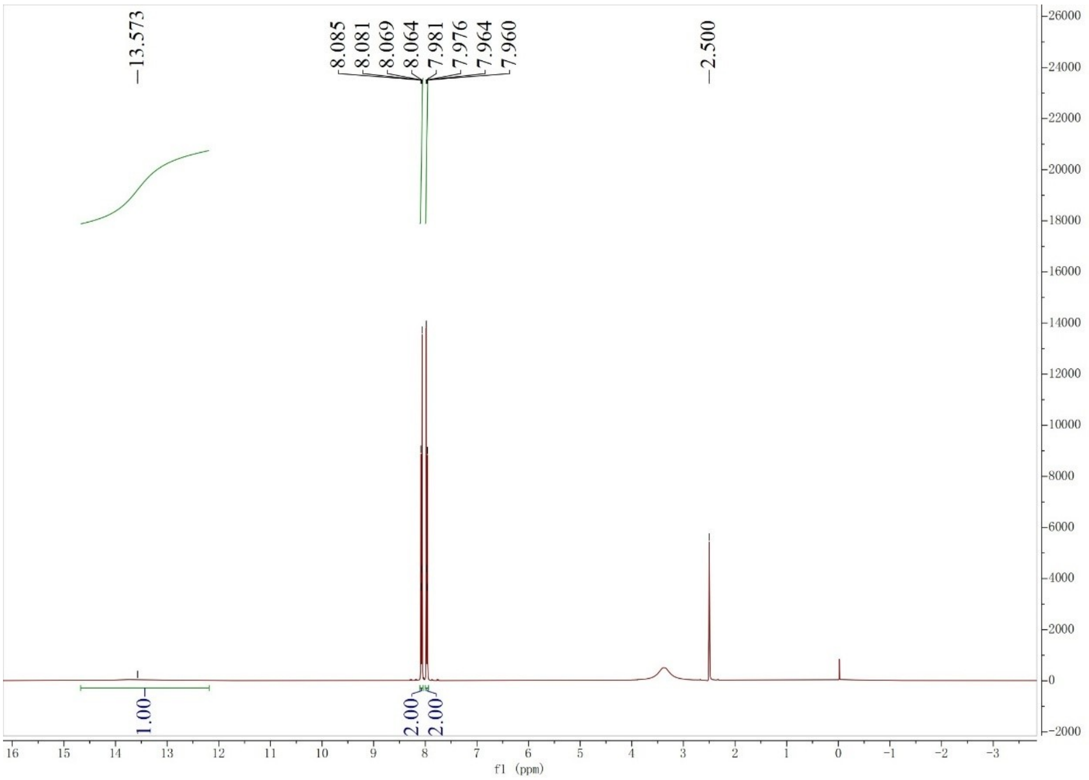
13.573
8.085
8.081
8.069
8.064
7.981
7.976
7.964
7.960
2.500
1.00
2.00
2.00
f1 (ppm)

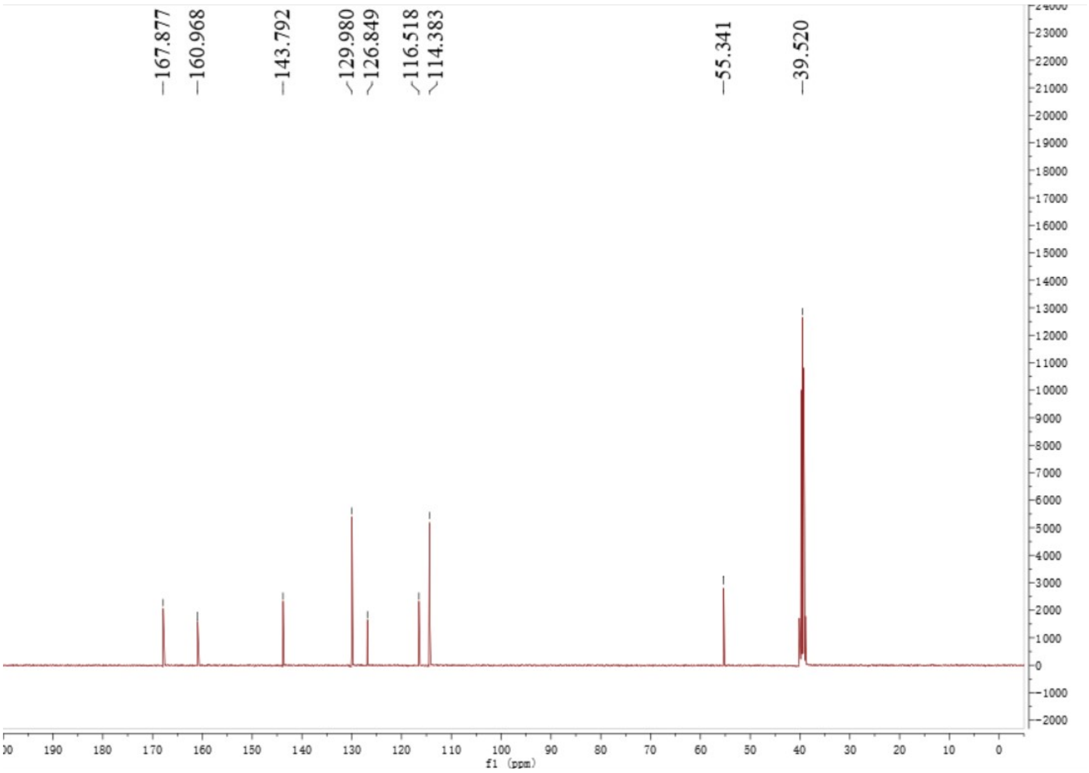
167.877
160.968
143.792
129.980
126.849
116.518
114.383
55.341
39.520
f1 (ppm)

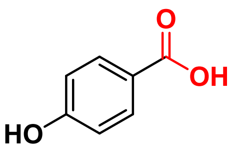

2g

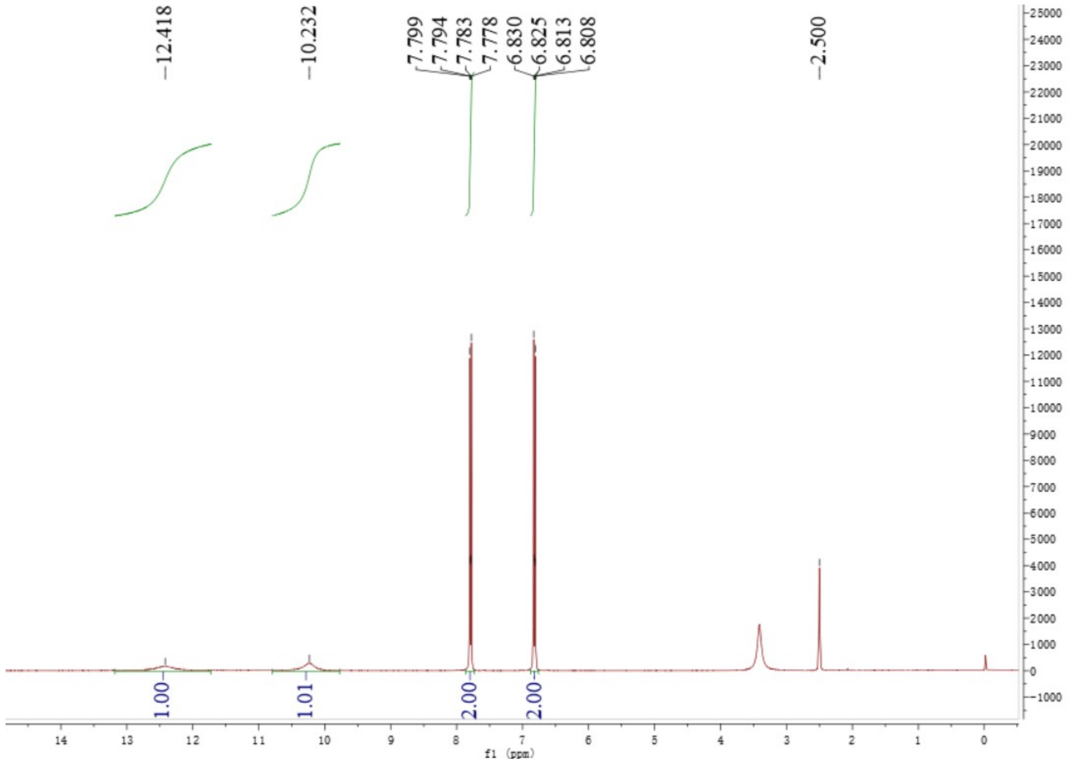
12.418
10.232
7.799
7.794
7.783
7.778
6.830
6.825
6.813
6.808
2.500
1.00
1.01
2.00
2.00
f1 (ppm)

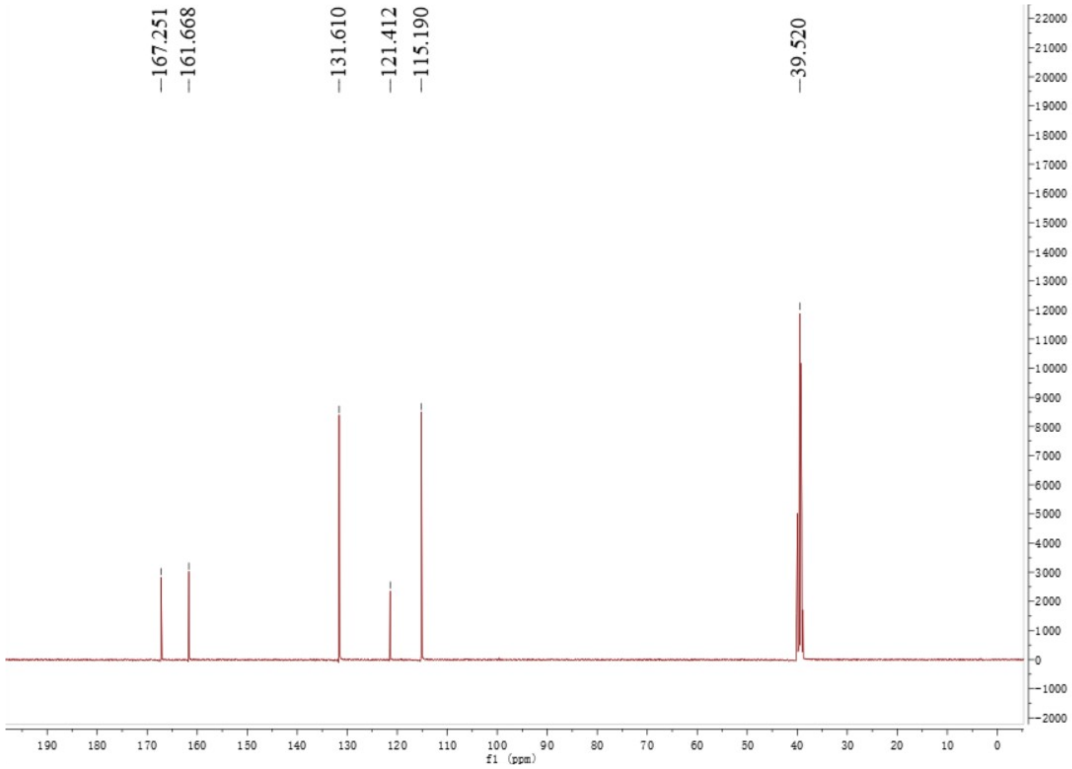
167.251
161.668
131.610
121.412
115.190
39.520
f1 (ppm)

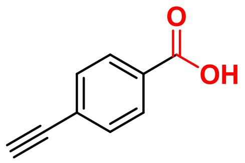
O
OH

2h

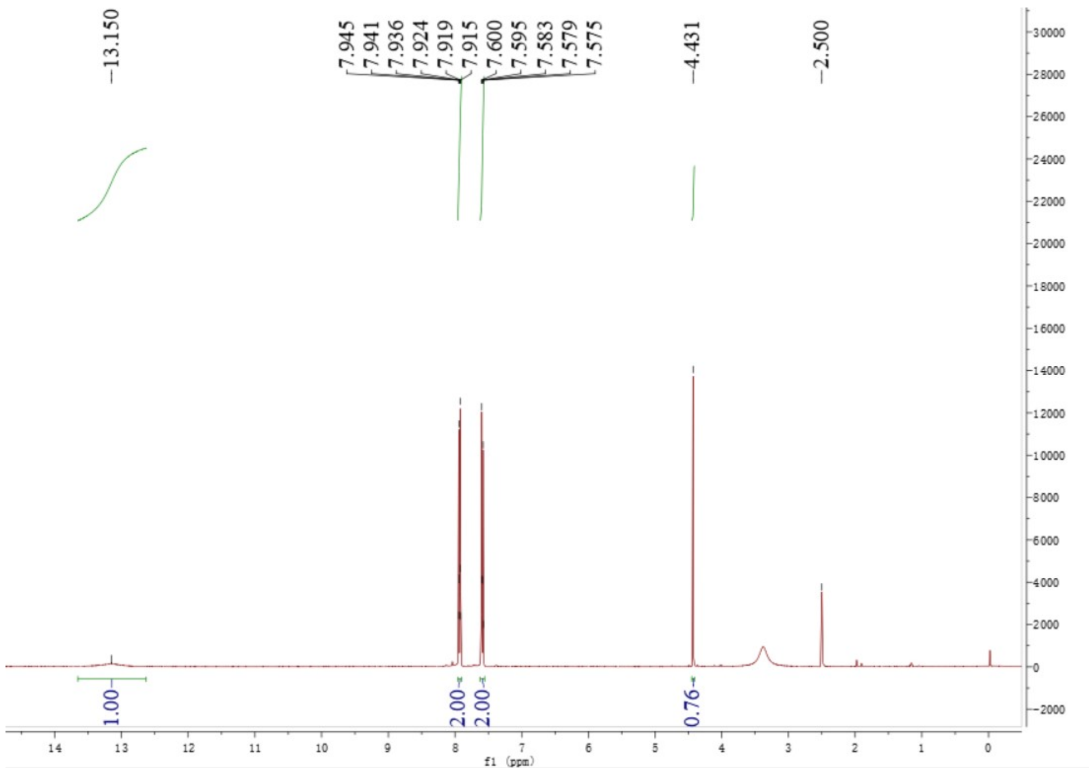
13.150
7.945
7.941
7.936
7.924
7.919
7.915
7.600
7.595
7.583
7.579
7.575
4.431
2.500
1.00
2.00
2.00
0.76
f1 (ppm)

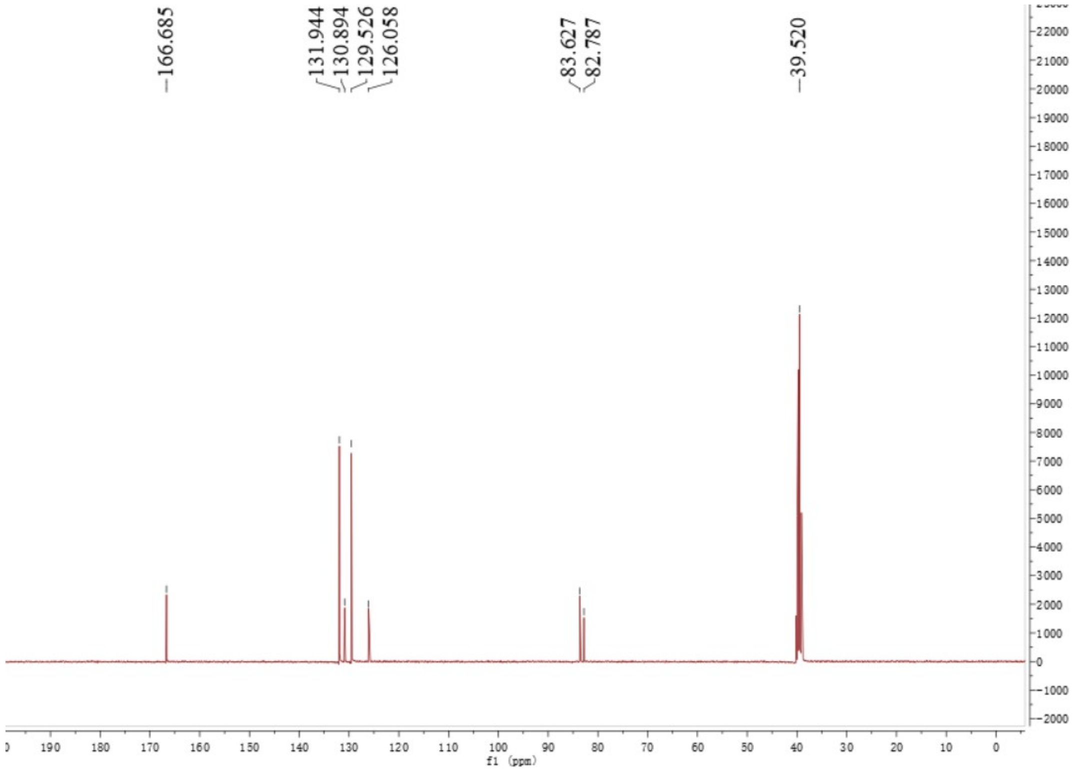
166.685
131.944
130.894
129.526
126.058
83.627
82.787
39.520
f1 (ppm)

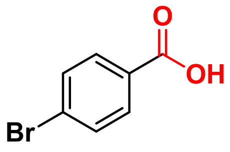
O
OH
Br

2i

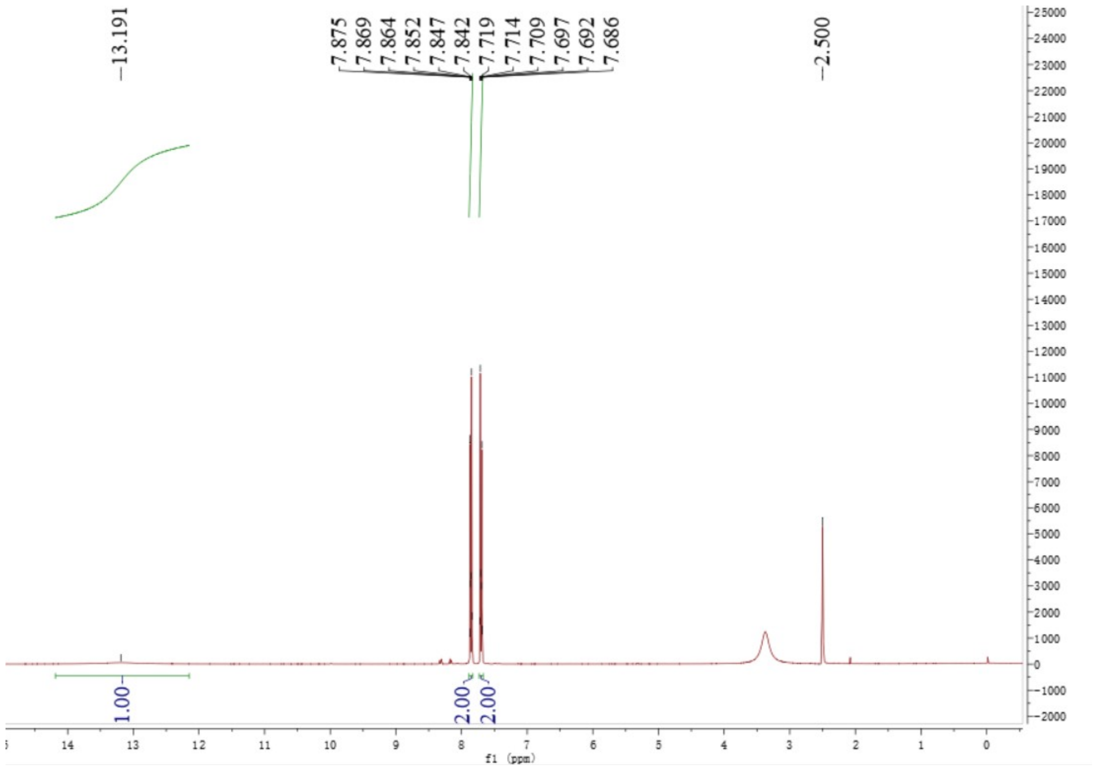
13.191
7.875
7.869
7.864
7.852
7.847
7.842
7.719
7.714
7.709
7.697
7.692
7.686
2.500
1.00
2.00
2.00
f1 (ppm)

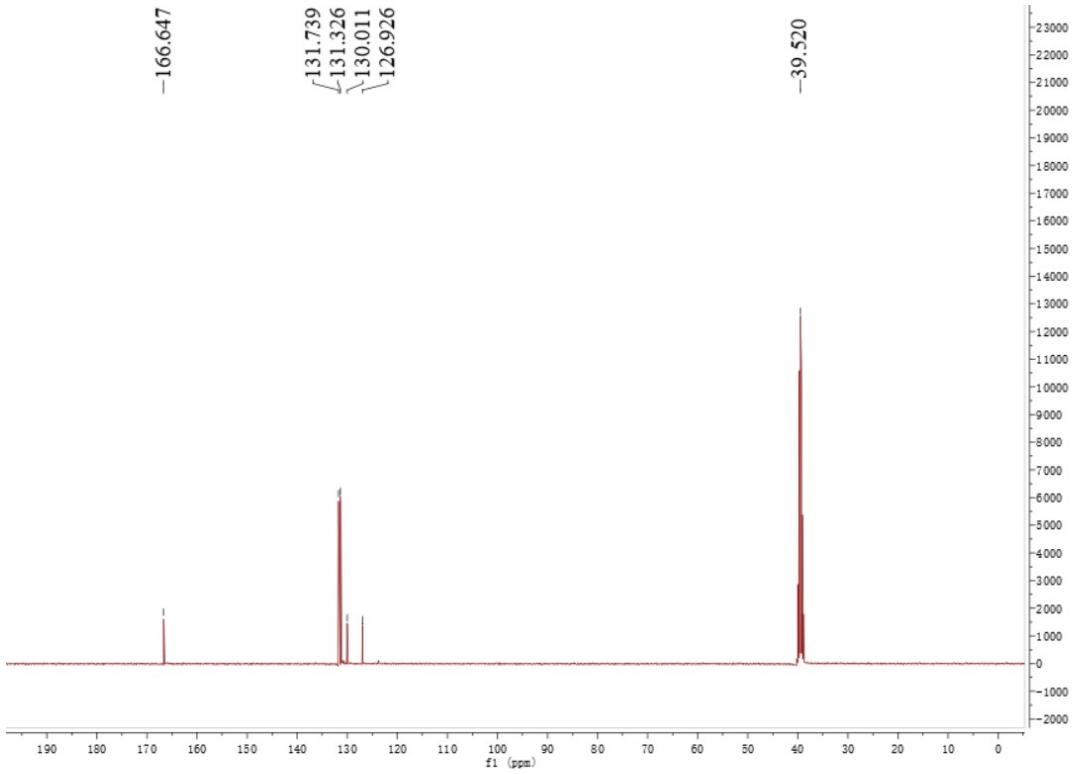
166.647
131.739
131.326
130.011
126.926
39.520
f1 (ppm)

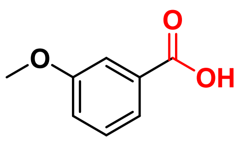

2j

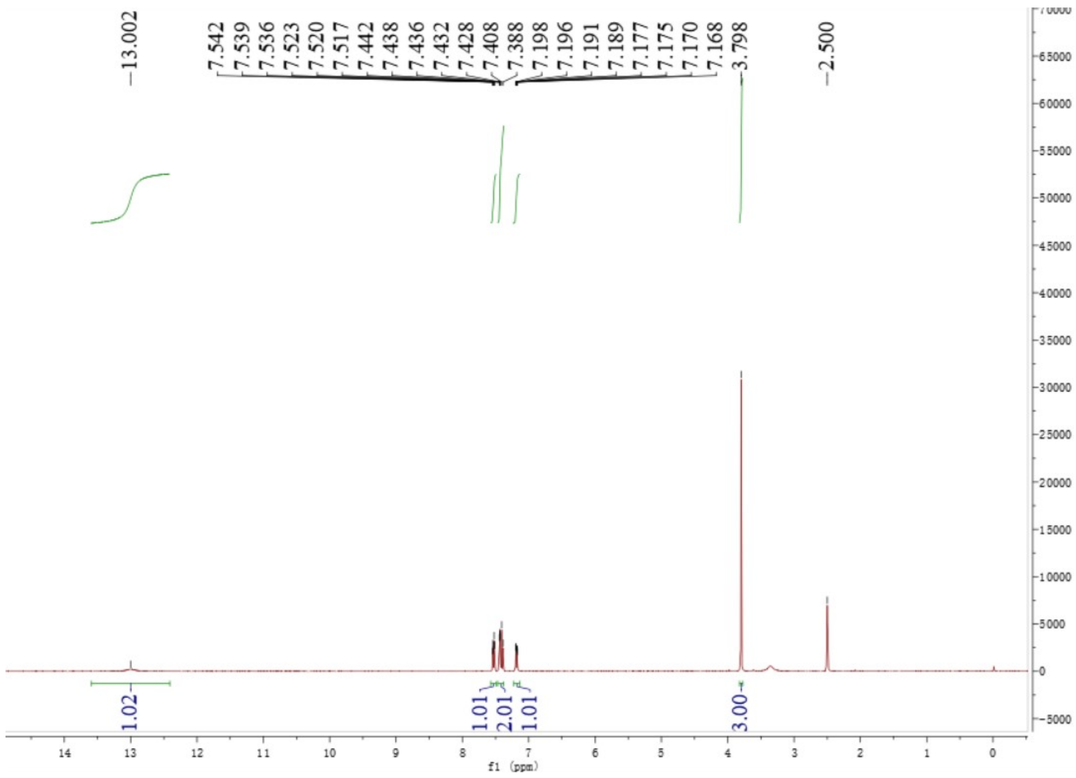
13.002
7.542
7.539
7.536
7.523
7.520
7.517
7.442
7.438
7.436
7.432
7.428
7.408
7.388
7.198
7.196
7.191
7.189
7.177
7.175
7.170
7.168
3.798
2.500
1.02
1.01
2.01
1.01
3.00
f1 (ppm)

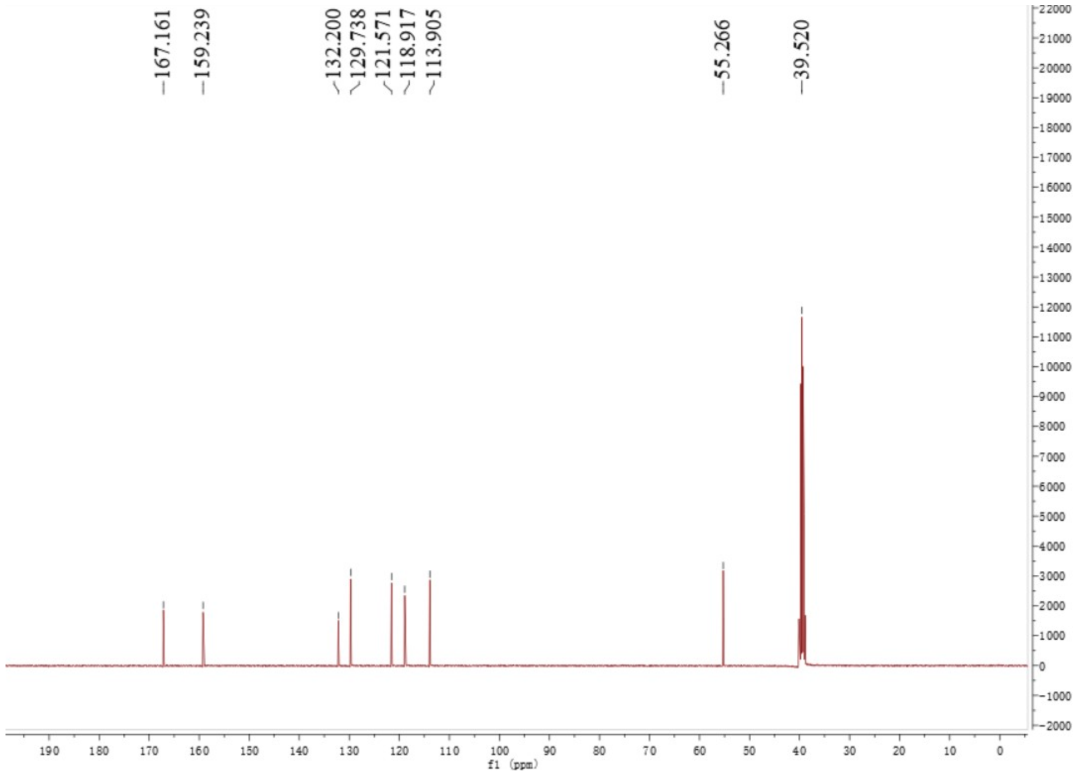
167.161
159.239
132.200
129.738
121.571
118.917
113.905
55.266
39.520
f1 (ppm)

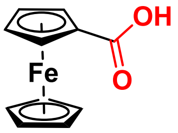
OH
Fe
O

2k

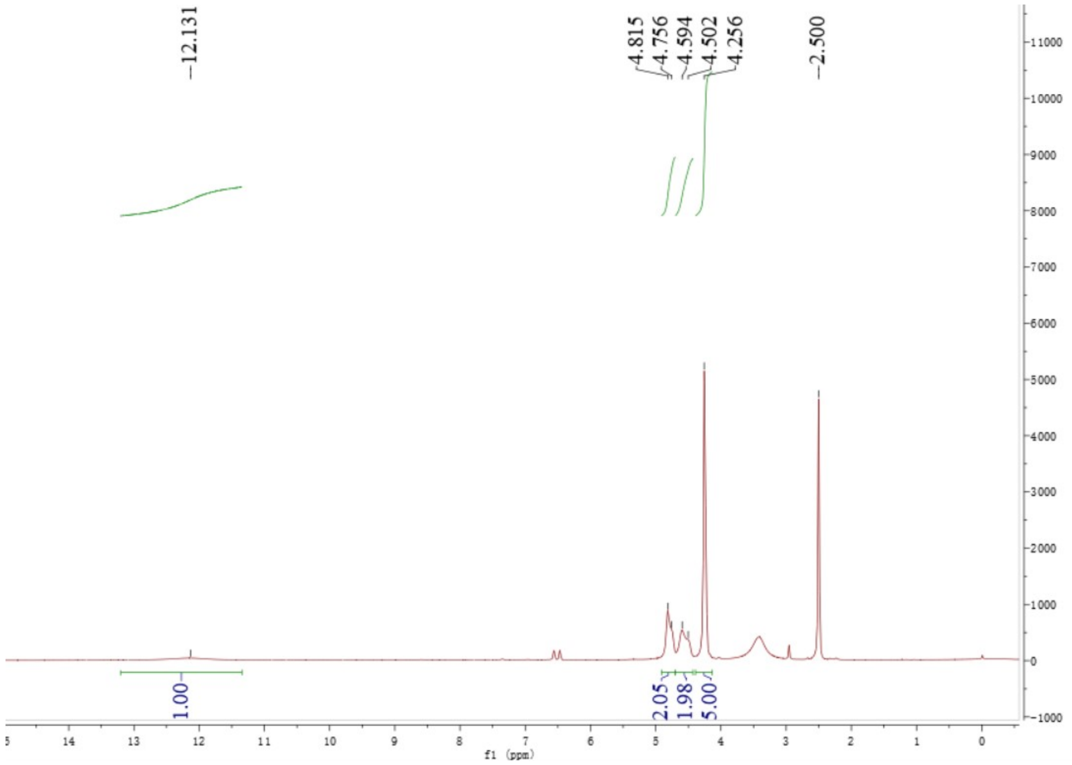
12.131
4.815
4.756
4.594
4.502
4.256
2.500
1.00
2.05
1.98
5.00
f1 (ppm)

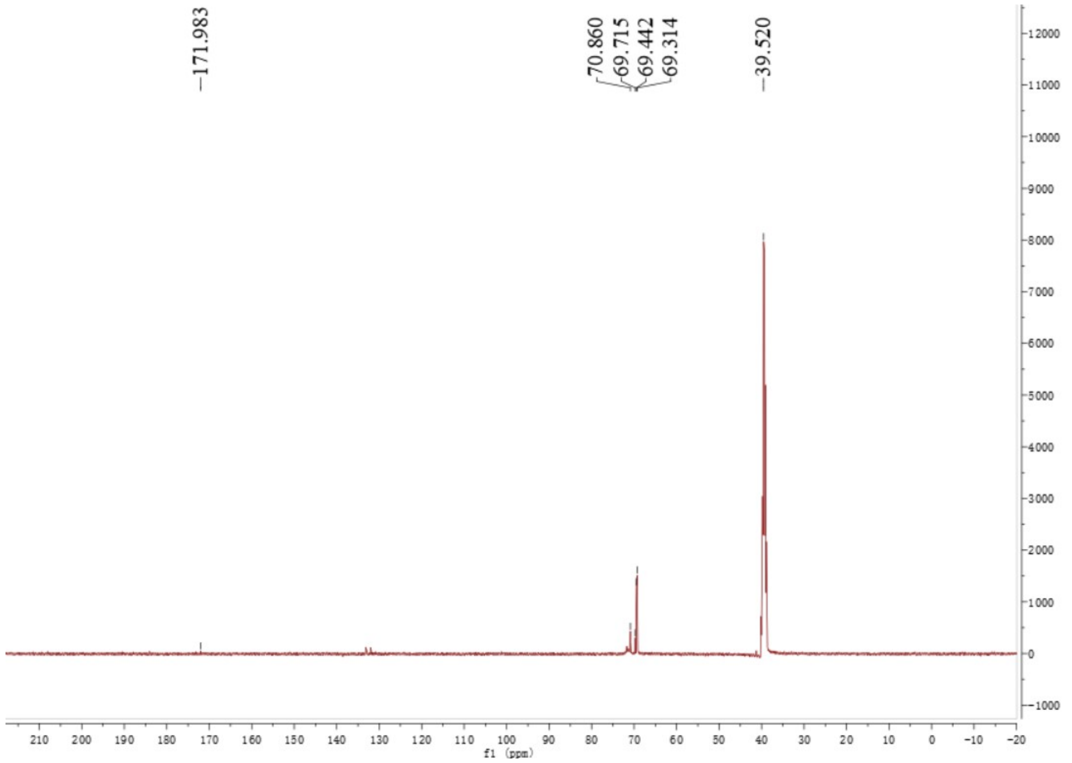
171.983
70.860
69.715
69.442
69.314
39.520
f1 (ppm)

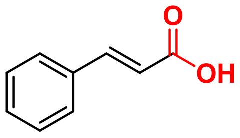
O
OH

2l

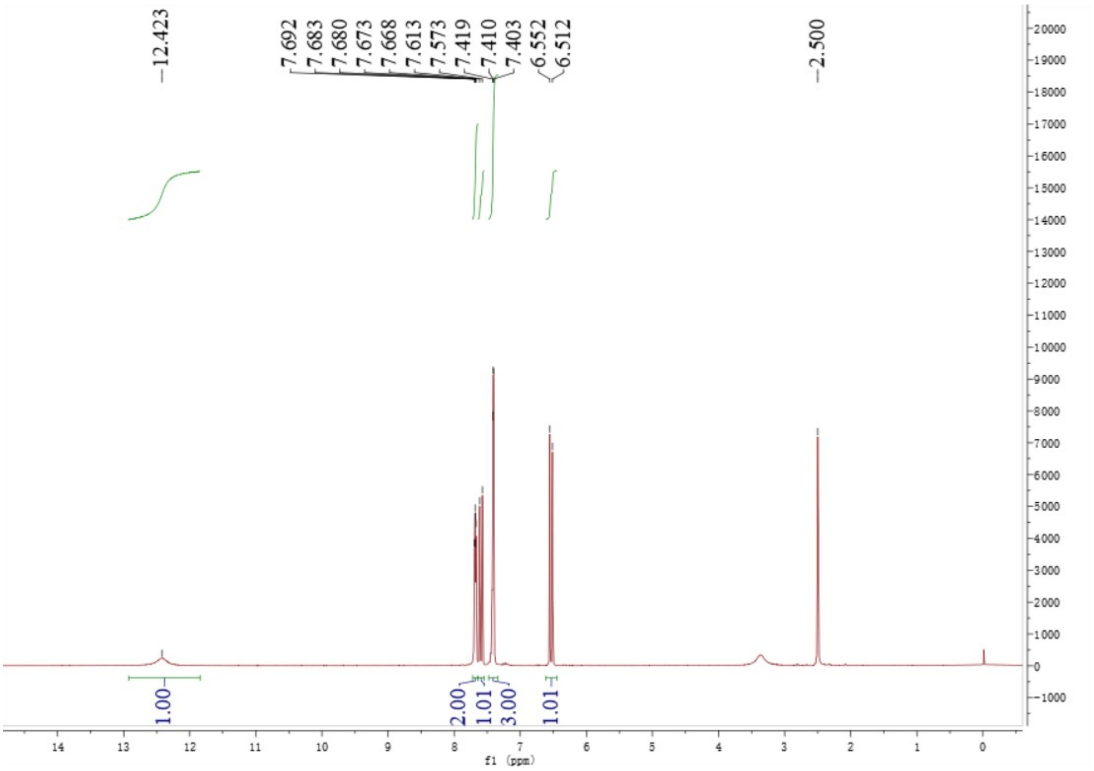
12.423
7.692
7.683
7.680
7.673
7.668
7.613
7.573
7.419
7.410
7.403
6.552
6.512
2.500
1.00
2.00
1.01
3.00
1.01
f1 (ppm)

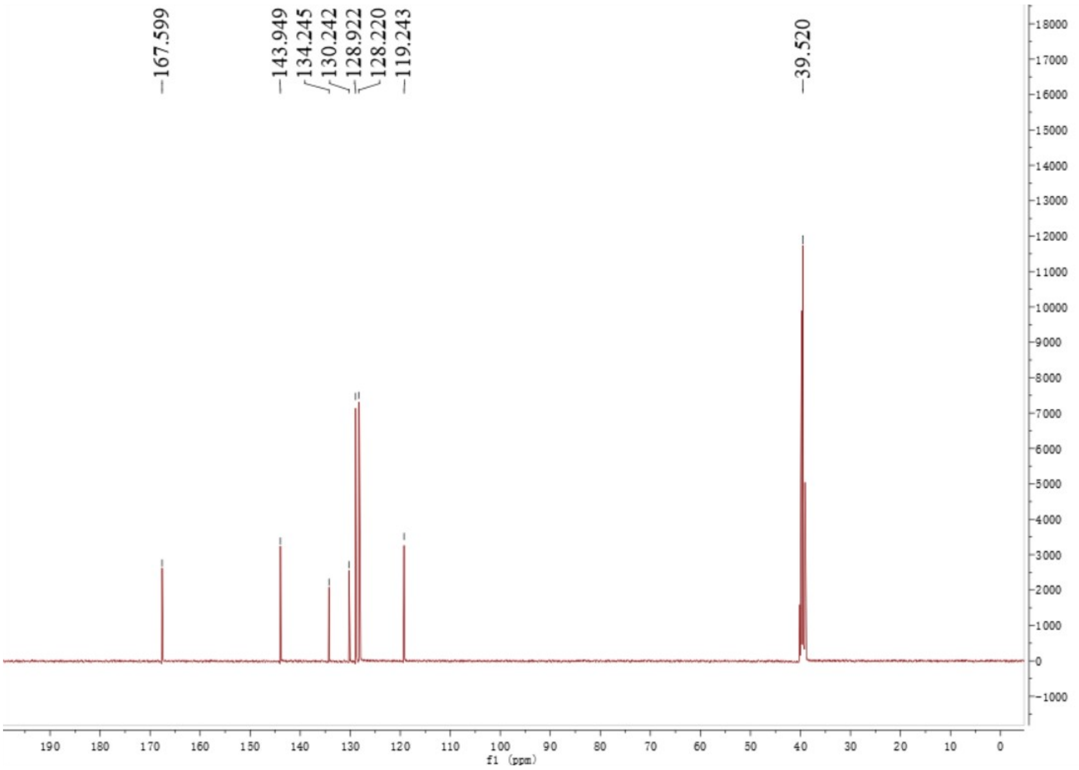
167.599
143.949
134.245
130.242
128.922
128.220
119.243
39.520
f1 (ppm)

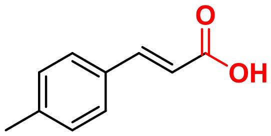
O
OH

2m

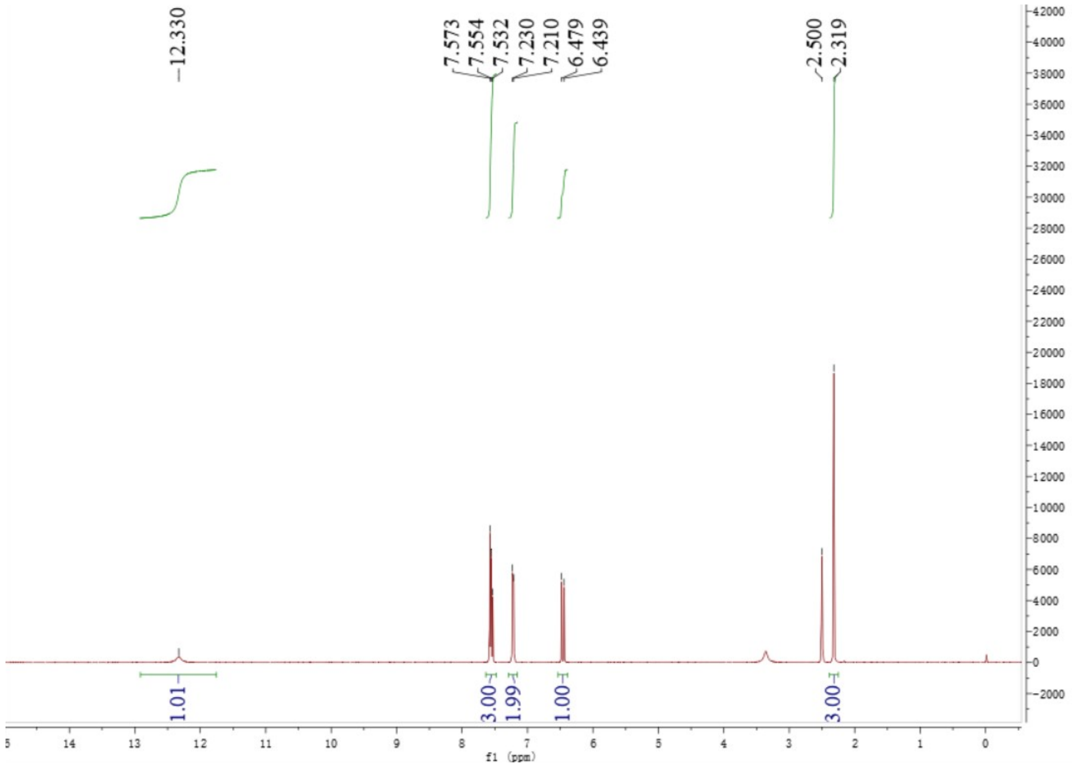
12.330
7.573
7.554
7.532
7.230
7.210
6.479
6.439
2.500
2.319
1.01
3.00
1.99
1.00
3.00
f1 (ppm)

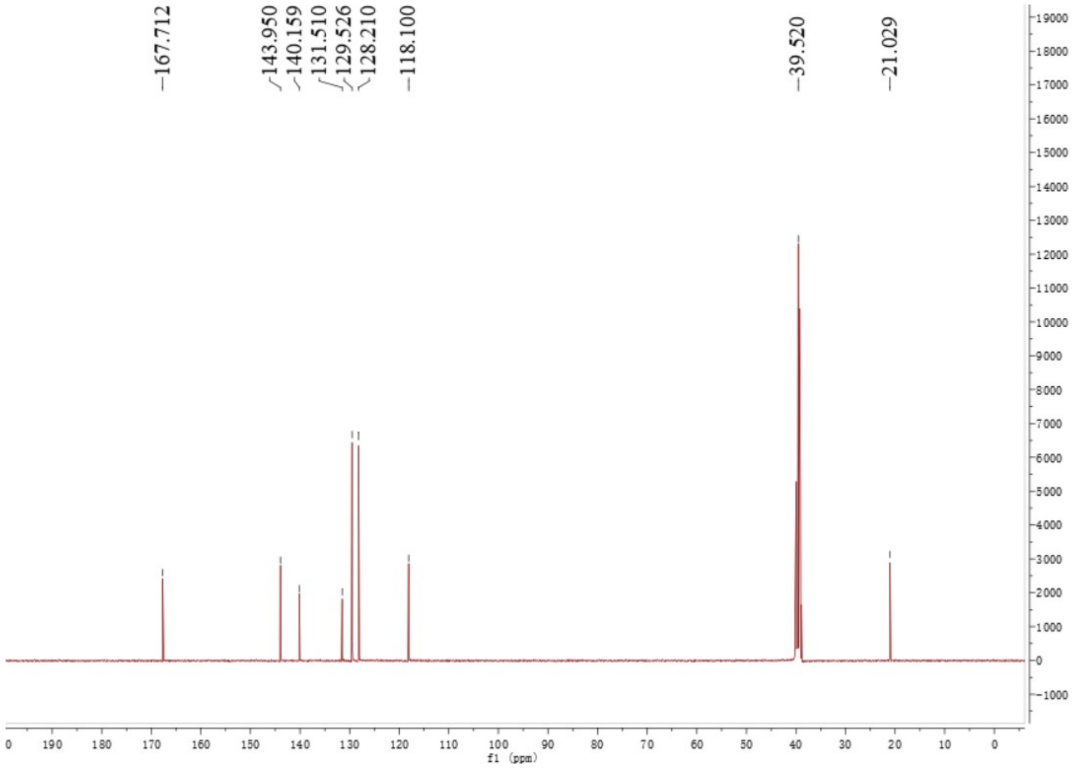
167.712
143.950
140.159
131.510
129.526
128.210
118.100
39.520
21.029
f1 (ppm)

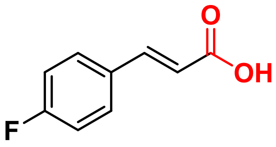
O
OH
F

2n

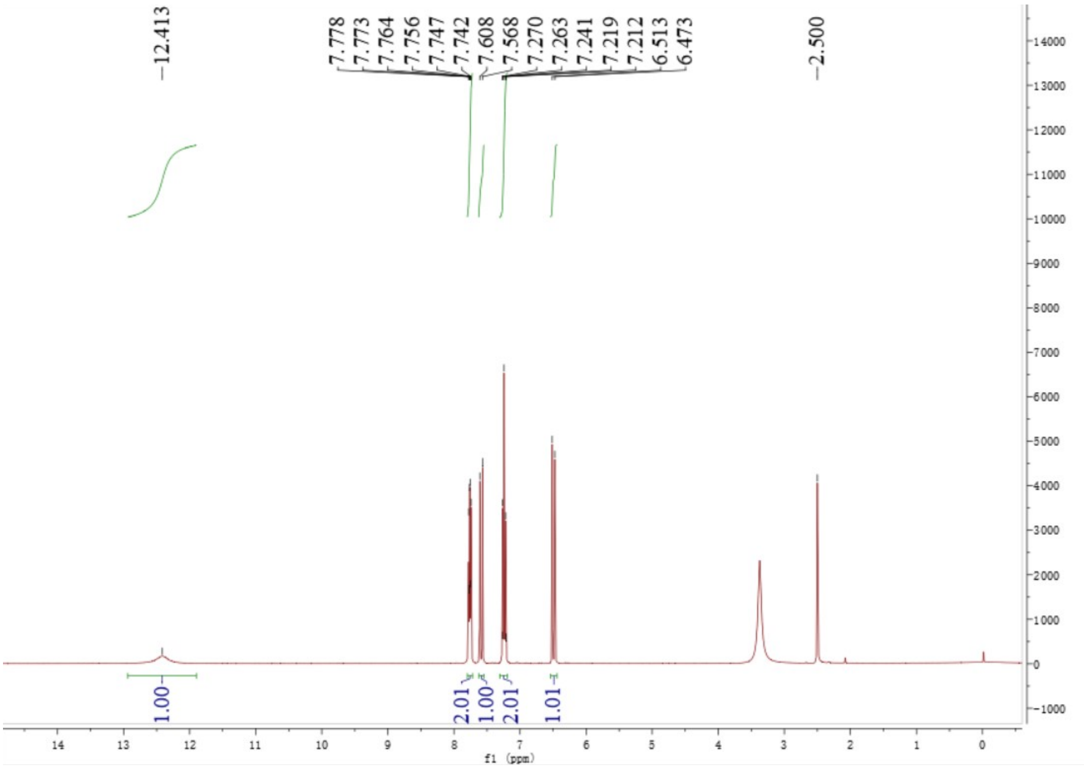
12.413
7.778
7.773
7.764
7.756
7.747
7.742
7.608
7.568
7.270
7.263
7.241
7.219
7.212
6.513
6.473
2.500
1.00
2.01
1.00
2.01
1.01
f1 (ppm)

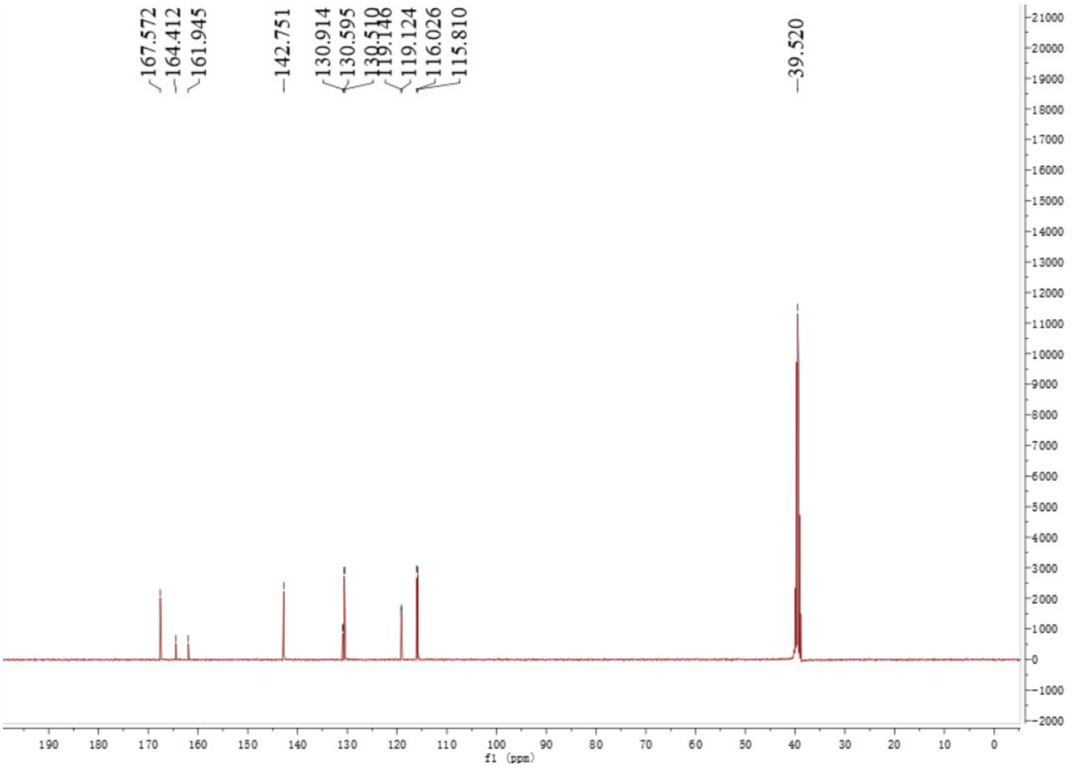
167.572
164.412
161.945
142.751
130.914
130.595
130.510
119.146
119.124
116.026
115.810
39.520
f1 (ppm)

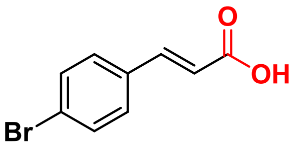

O
OH
Br

2o

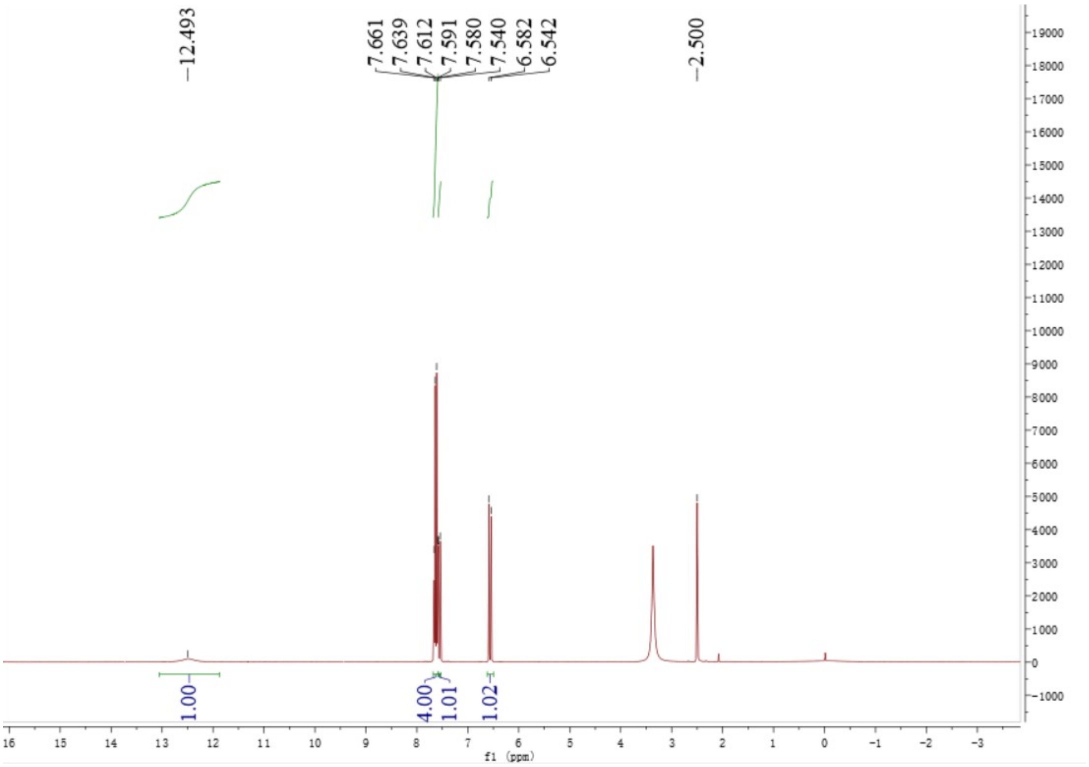

12.493
7.661
7.639
7.612
7.591
7.580
7.540
6.582
6.542
2.500
1.00
4.00
1.01
1.02
f1 (ppm)

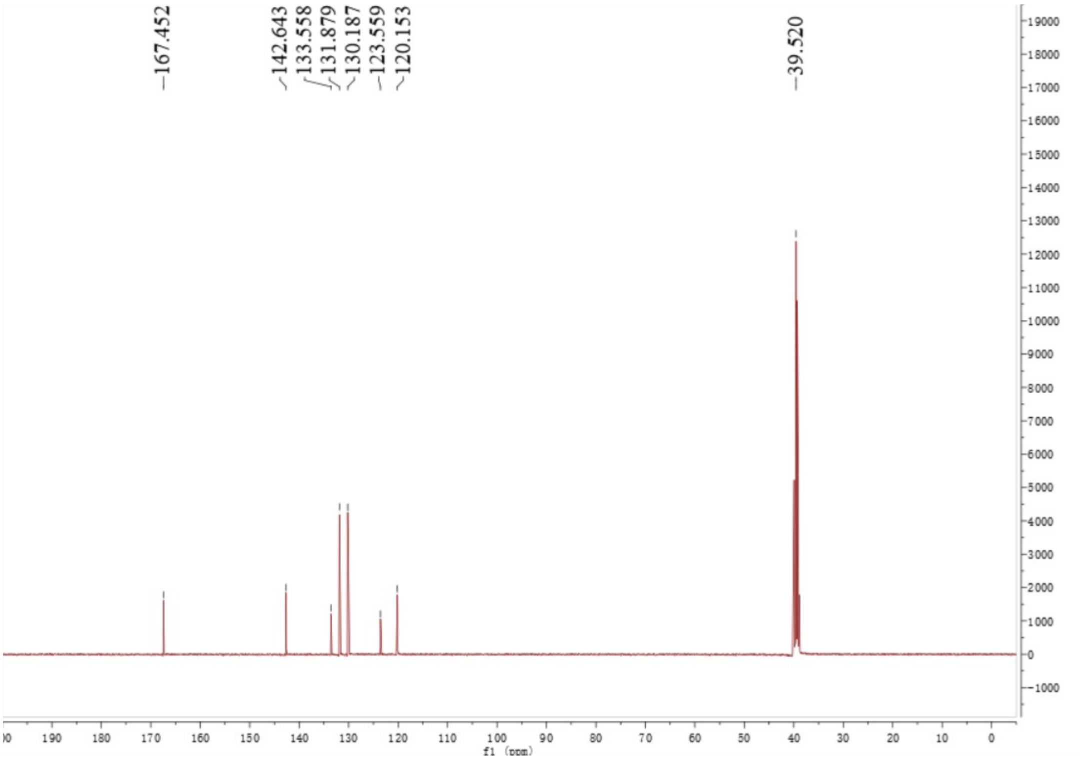

167.452
142.643
133.558
131.879
130.187
123.559
120.153
39.520
f1 (ppm)

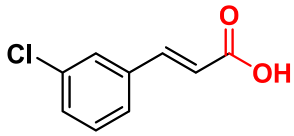

2p

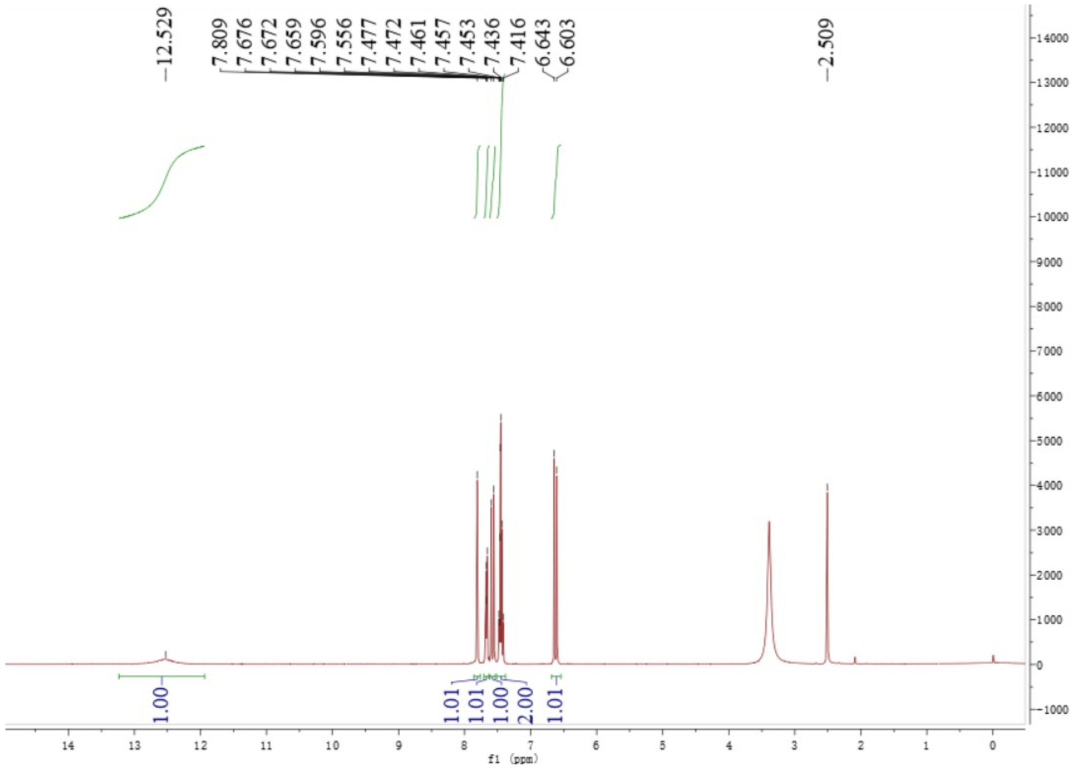

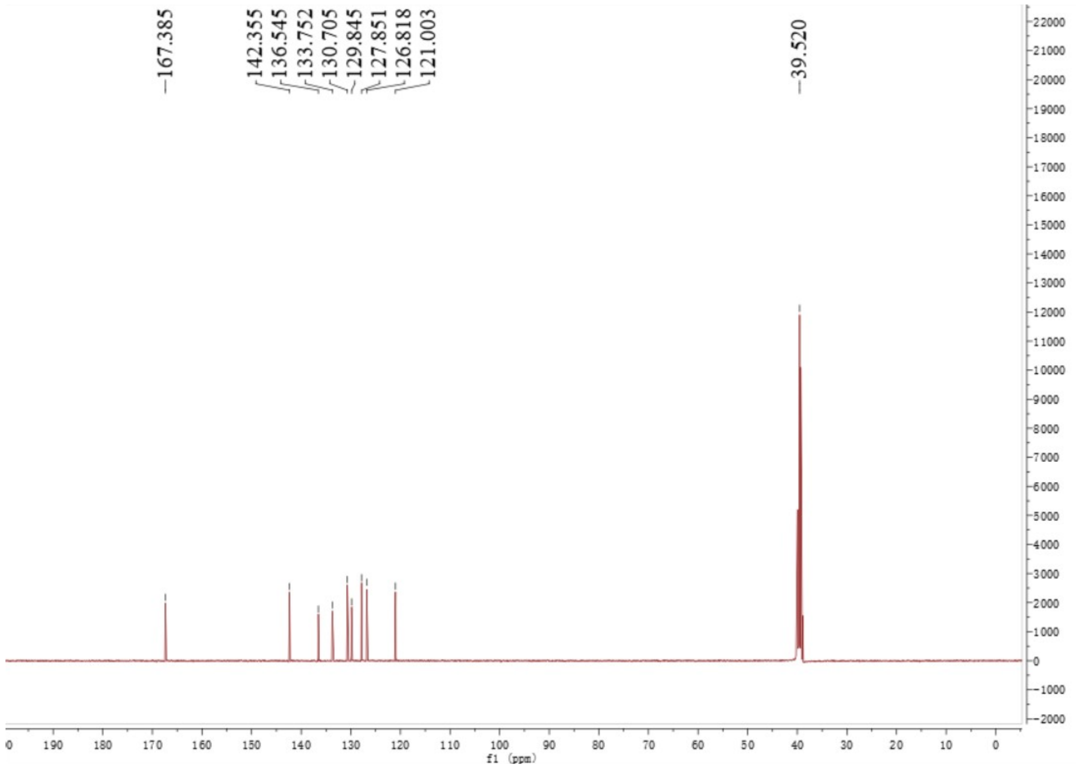

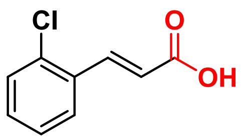
Cl
O
OH

2q

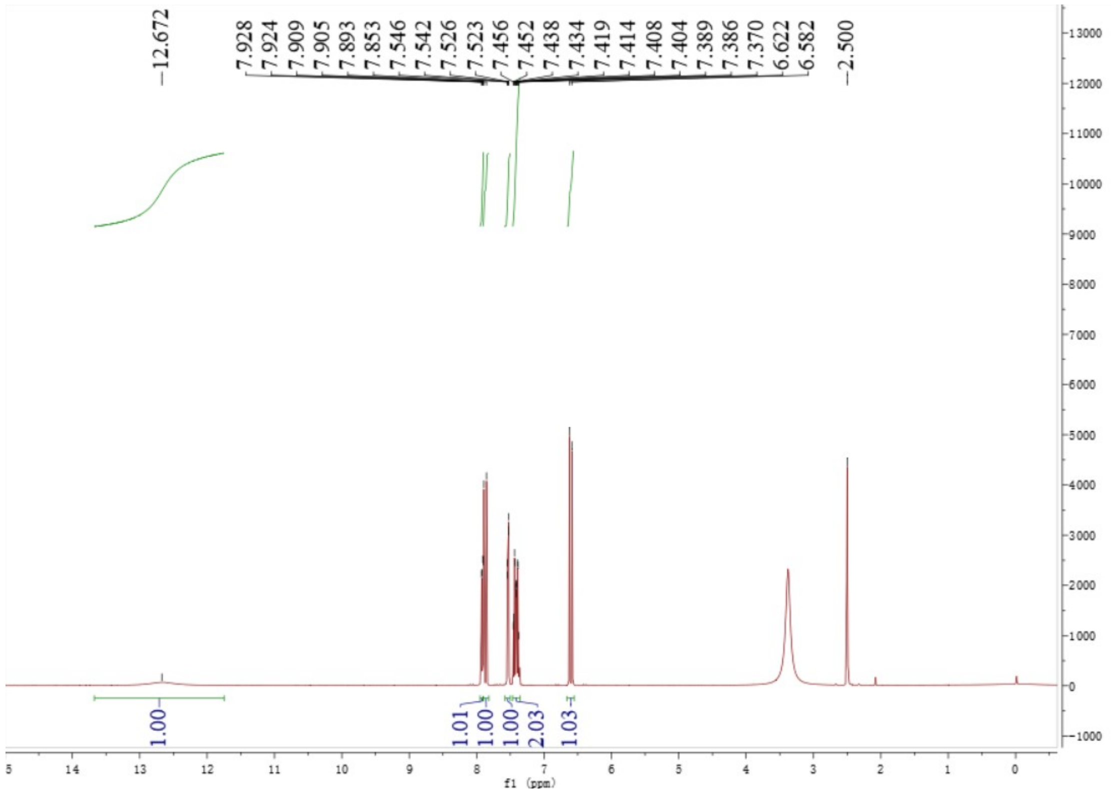
12.672
7.928
7.924
7.909
7.905
7.893
7.853
7.546
7.542
7.526
7.523
7.456
7.452
7.438
7.434
7.419
7.414
7.408
7.404
7.389
7.386
7.370
6.622
6.582
2.500
1.00
1.01
1.00
1.00
2.03
1.03
f1 (ppm)

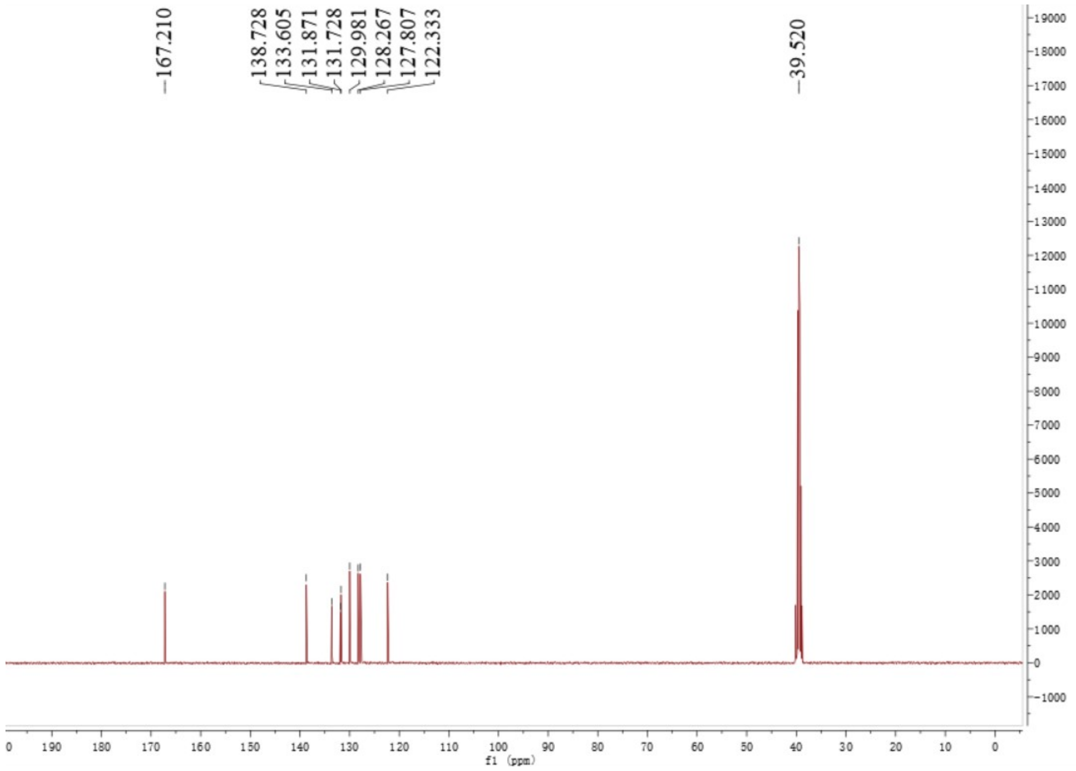
167.210
138.728
133.605
131.871
131.728
129.981
128.267
127.807
122.333
39.520
f1 (ppm)

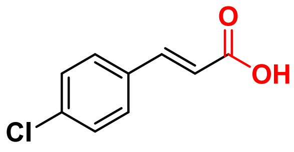
O
OH
Cl

2r

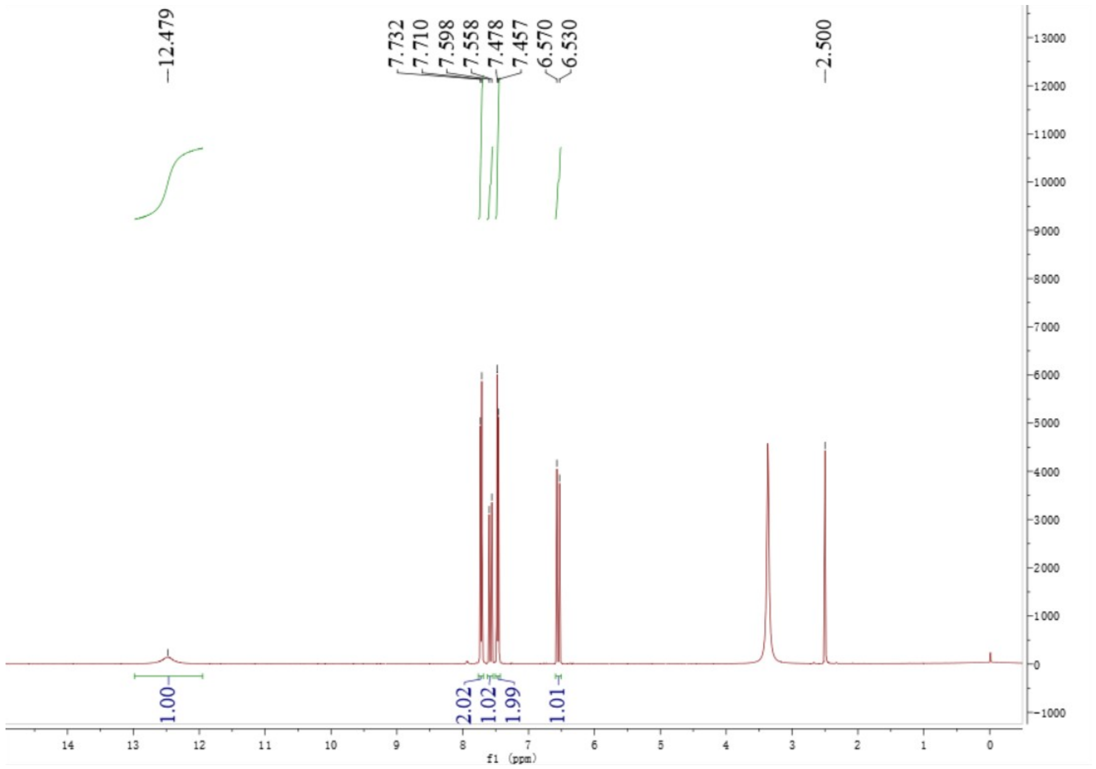
12.479
7.732
7.710
7.598
7.558
7.478
7.457
6.570
6.530
2.500
1.00
2.02
1.02
1.99
1.01
f1 (ppm)

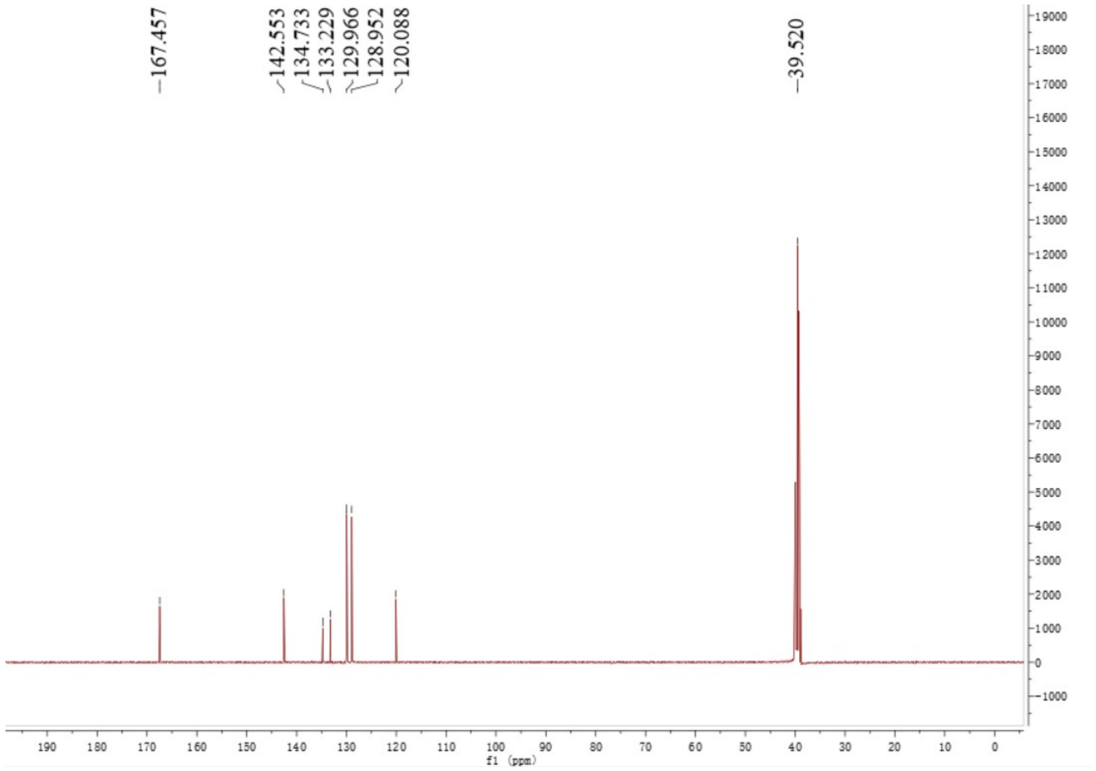
167.457
142.553
134.733
133.229
129.966
128.952
120.088
39.520
f1 (ppm)

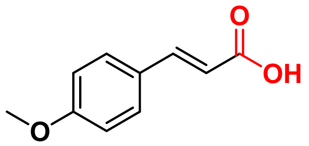

2s

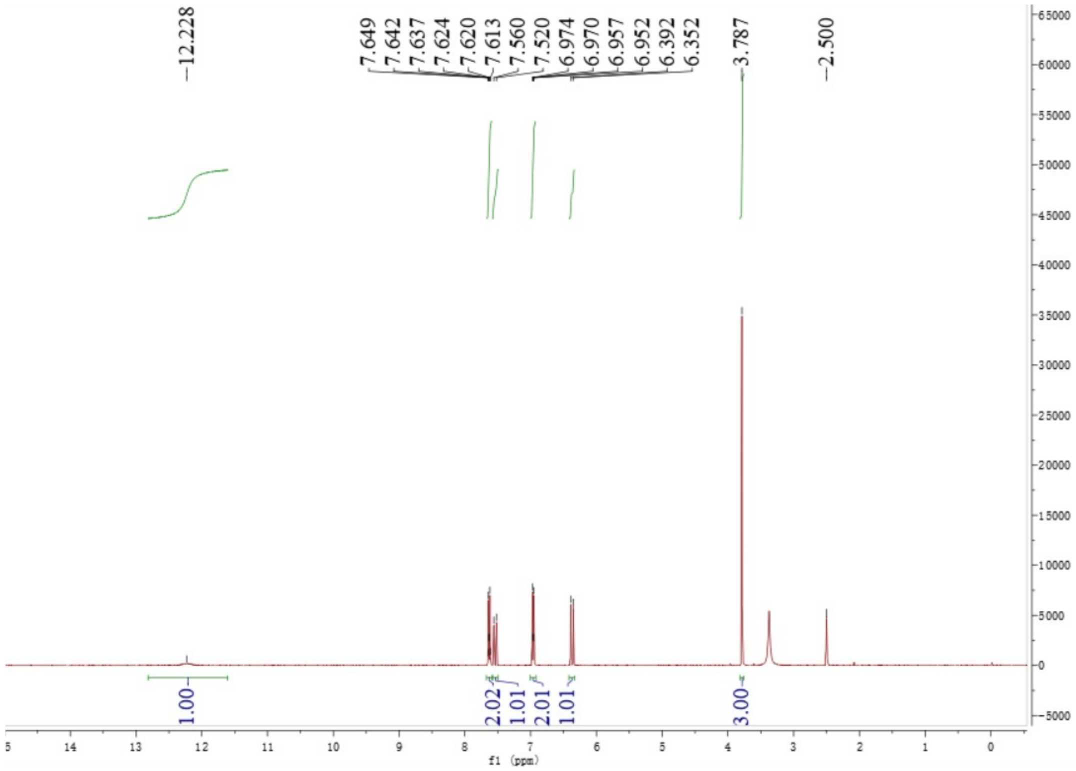

12.228
7.649
7.642
7.637
7.624
7.620
7.613
7.560
7.520
6.974
6.970
6.957
6.952
6.392
6.352
3.787
2.500
1.00
2.02
1.01
2.01
1.01
3.00
f1 (ppm)

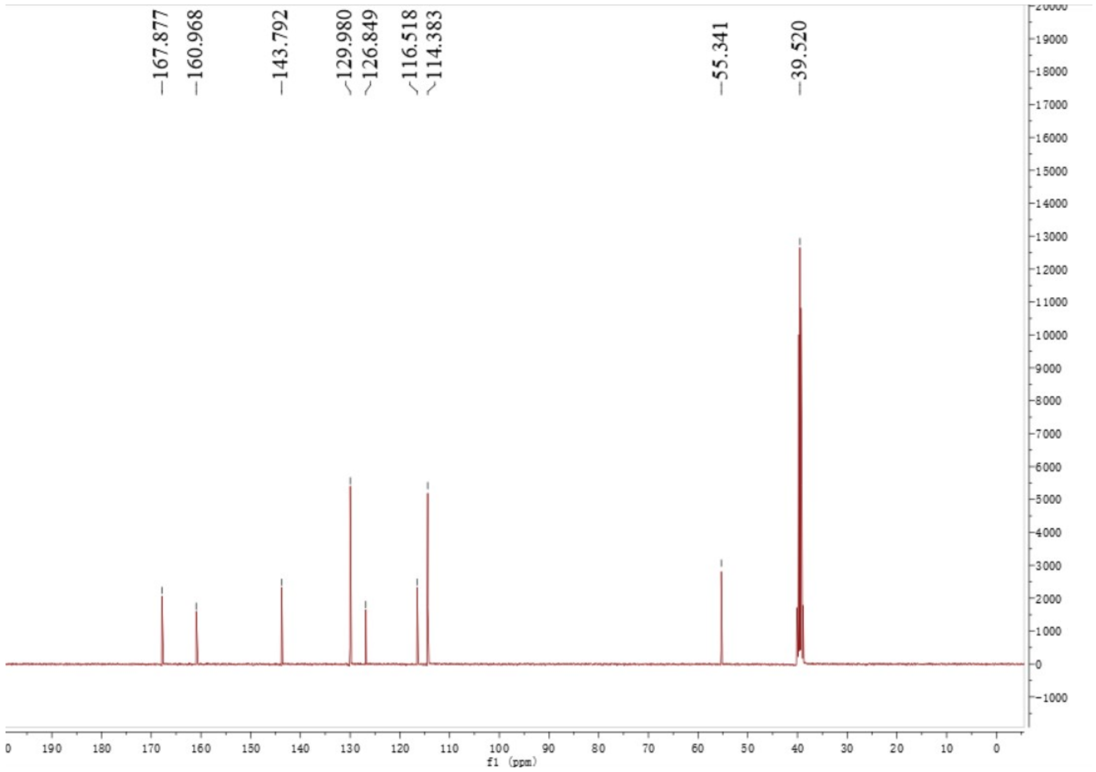

167.877
160.968
143.792
129.980
126.849
116.518
114.383
55.341
39.520
f1 (ppm)

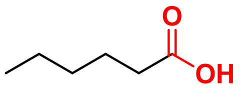
O
OH

2t

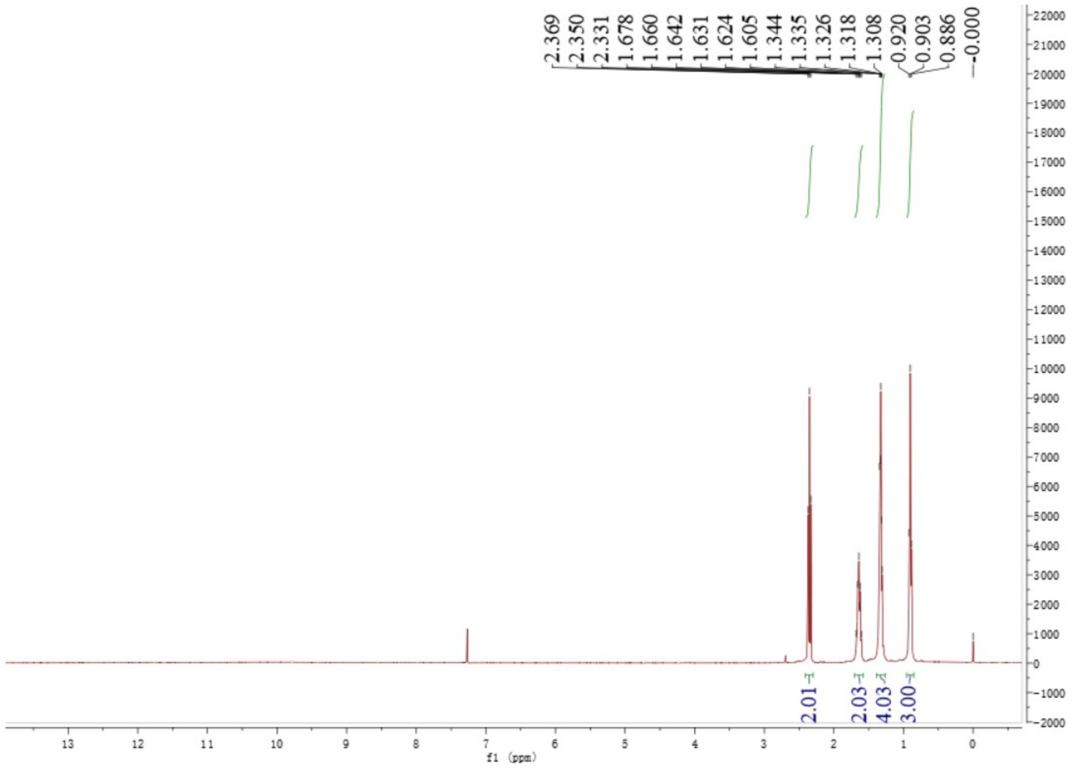
2.369
2.350
2.331
1.678
1.660
1.642
1.631
1.624
1.605
1.344
1.335
1.326
1.318
1.308
0.920
0.903
0.886
-0.000
2.01
2.03
4.03
3.00
f1 (ppm)

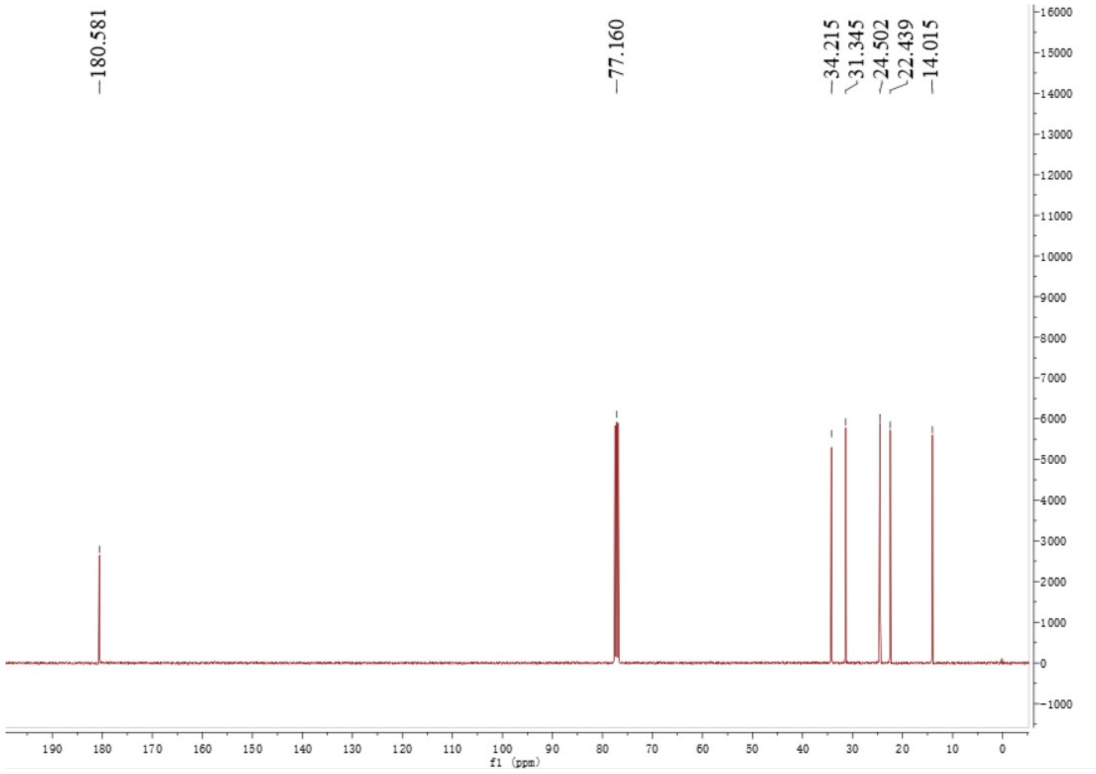
180.581
77.160
34.215
31.345
24.502
22.439
14.015
f1 (ppm)

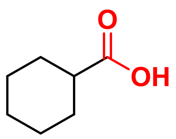

2u

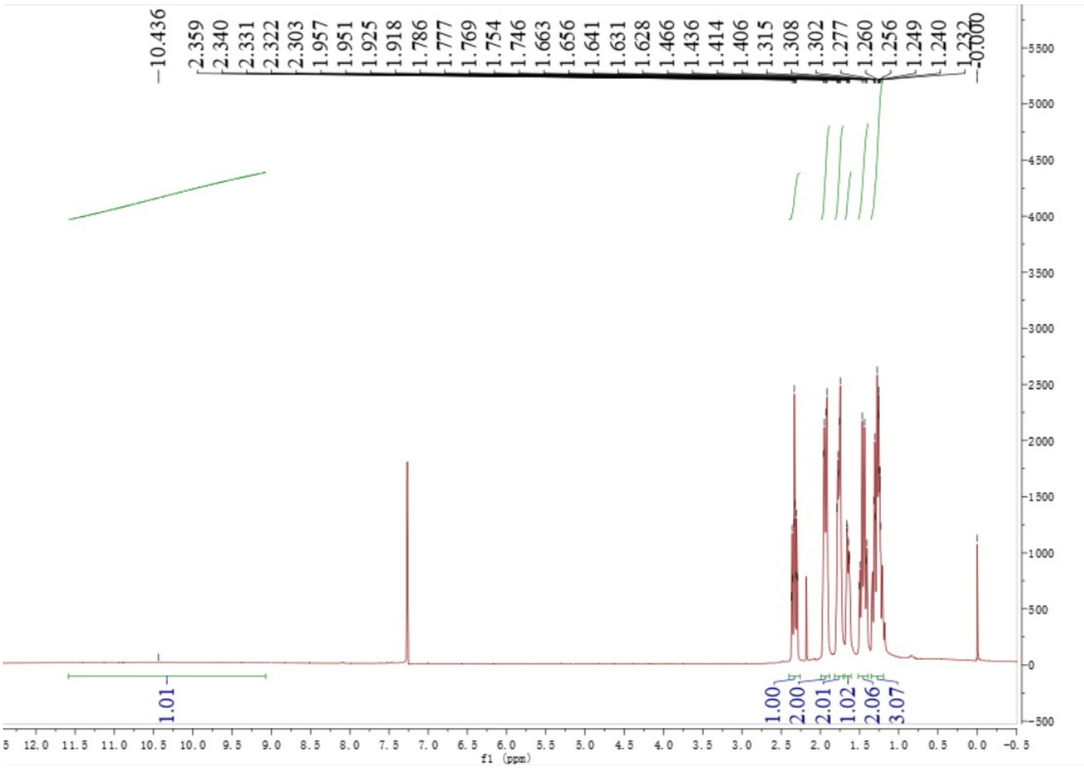

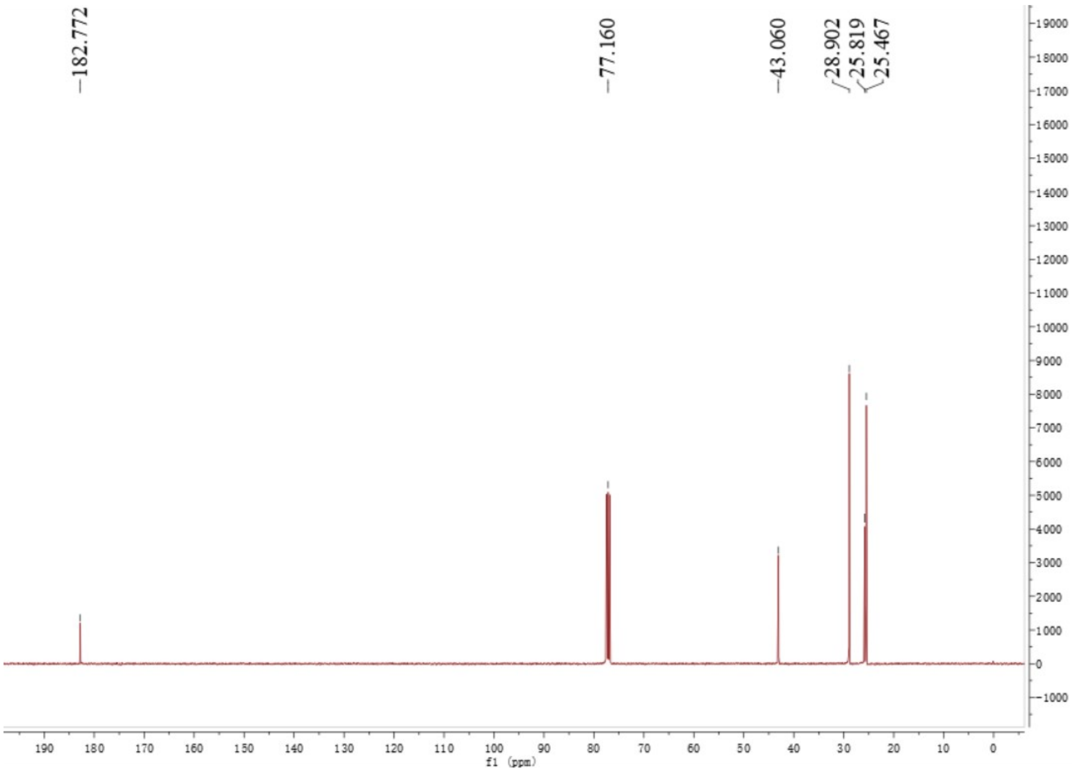

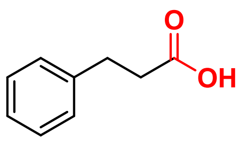
O
OH

2v

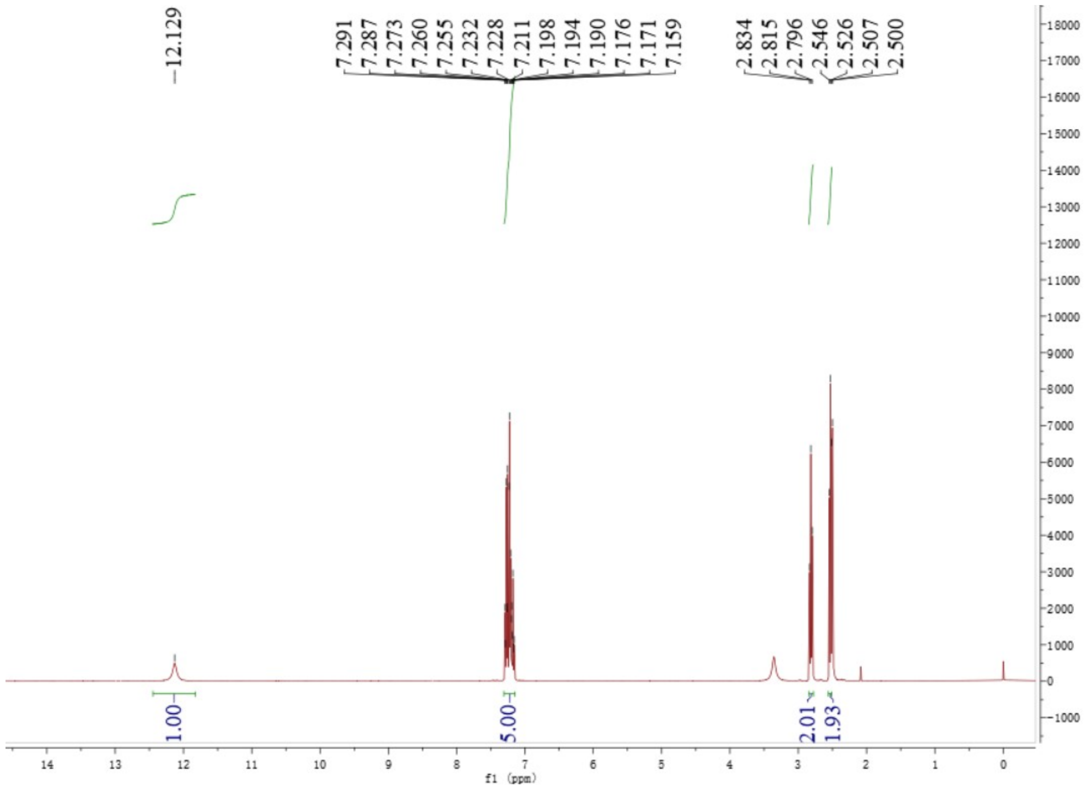
12.129
7.291
7.287
7.273
7.260
7.255
7.232
7.228
7.211
7.198
7.194
7.190
7.176
7.171
7.159
2.834
2.815
2.796
2.546
2.526
2.507
2.500
1.00
5.00
2.01
1.93
f1 (ppm)

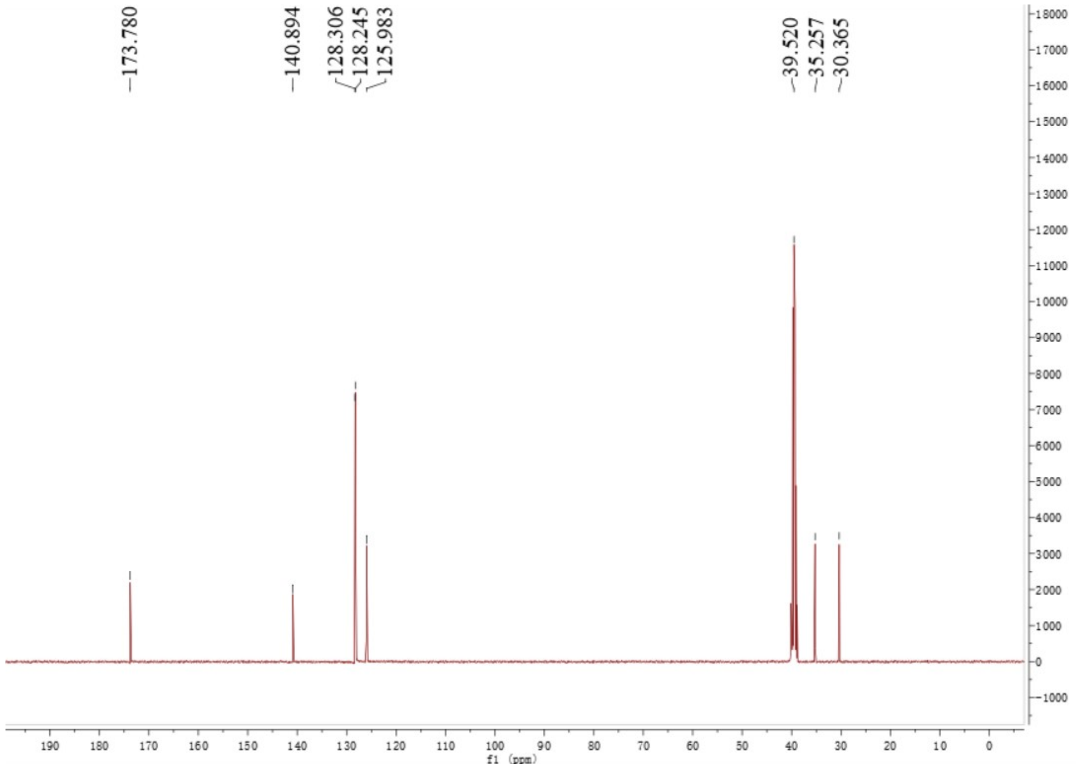
173.780
140.894
128.306
128.245
125.983
39.520
35.257
30.365
f1 (ppm)

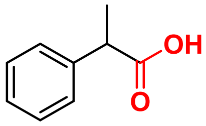
OH
O

4a

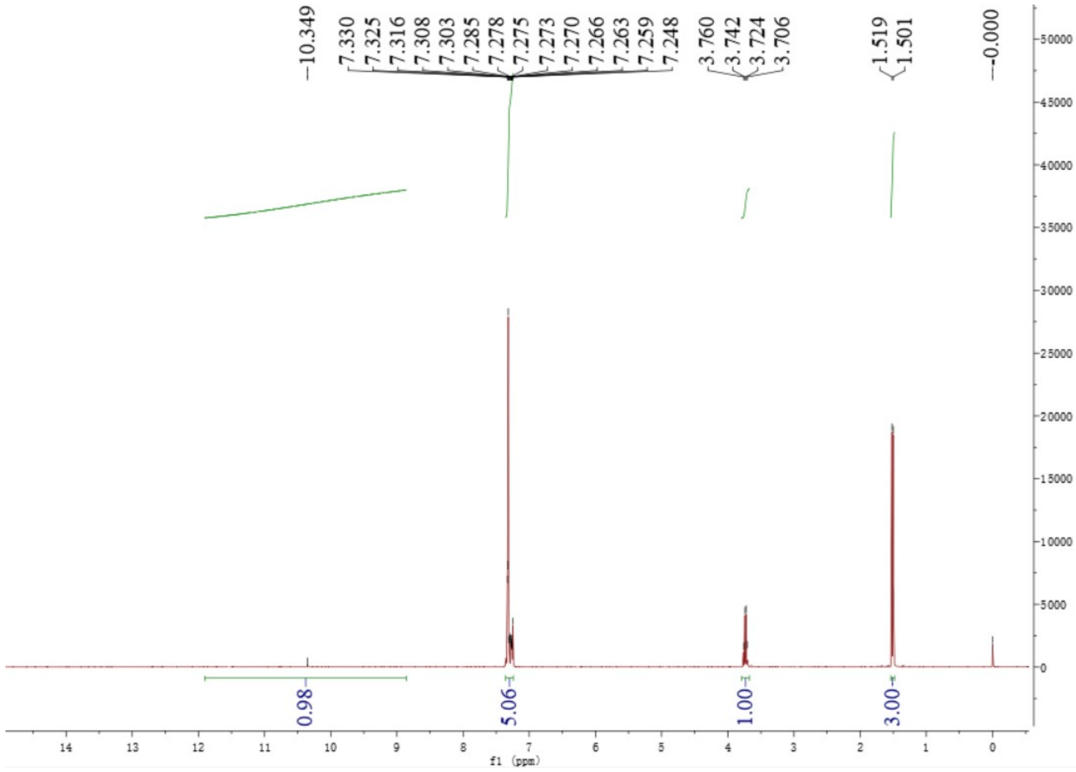
10.349
7.330
7.325
7.316
7.308
7.303
7.285
7.278
7.275
7.273
7.270
7.266
7.263
7.259
7.248
3.760
3.742
3.724
3.706
1.519
1.501
-0.000
0.98
5.06
1.00
3.00
f1 (ppm)

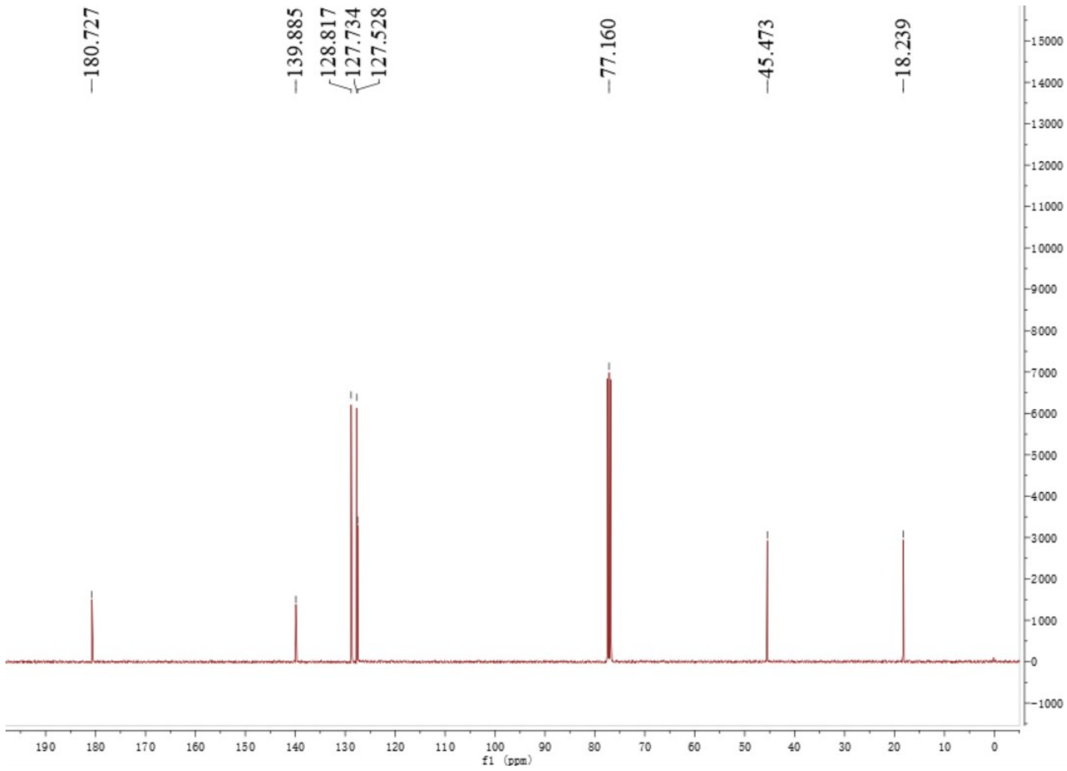
180.727
139.885
128.817
127.734
127.528
77.160
45.473
18.239
f1 (ppm)

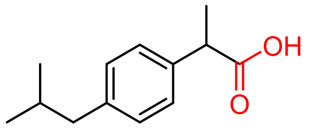
OH
O

4b

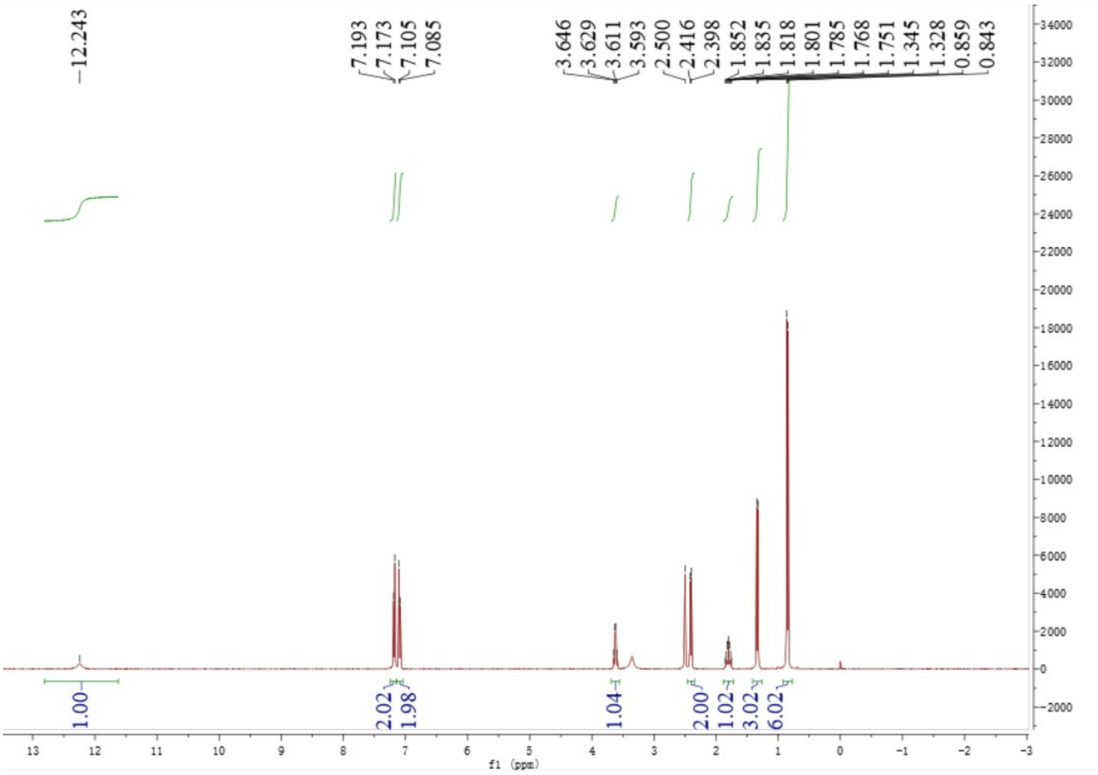
12.243
7.193
7.173
7.105
7.085
3.646
3.629
3.611
3.593
2.500
2.416
2.398
1.852
1.835
1.818
1.801
1.785
1.768
1.751
1.345
1.328
0.859
0.843
1.00
2.02
1.98
1.04
2.00
1.02
3.02
6.02
f1 (ppm)

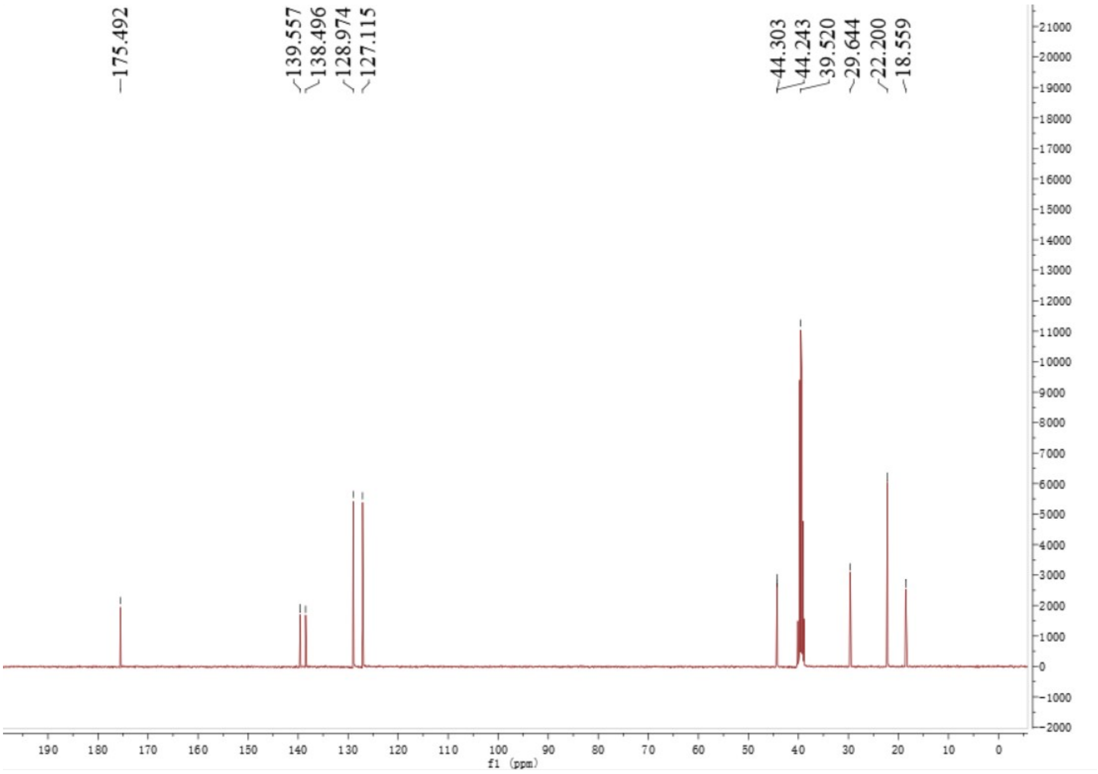
175.492
139.557
138.496
128.974
127.115
44.303
44.243
39.520
29.644
22.200
18.559
f1 (ppm)

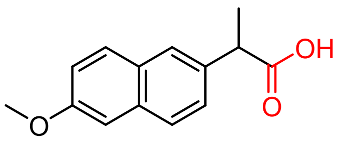
OH
O
O

4c

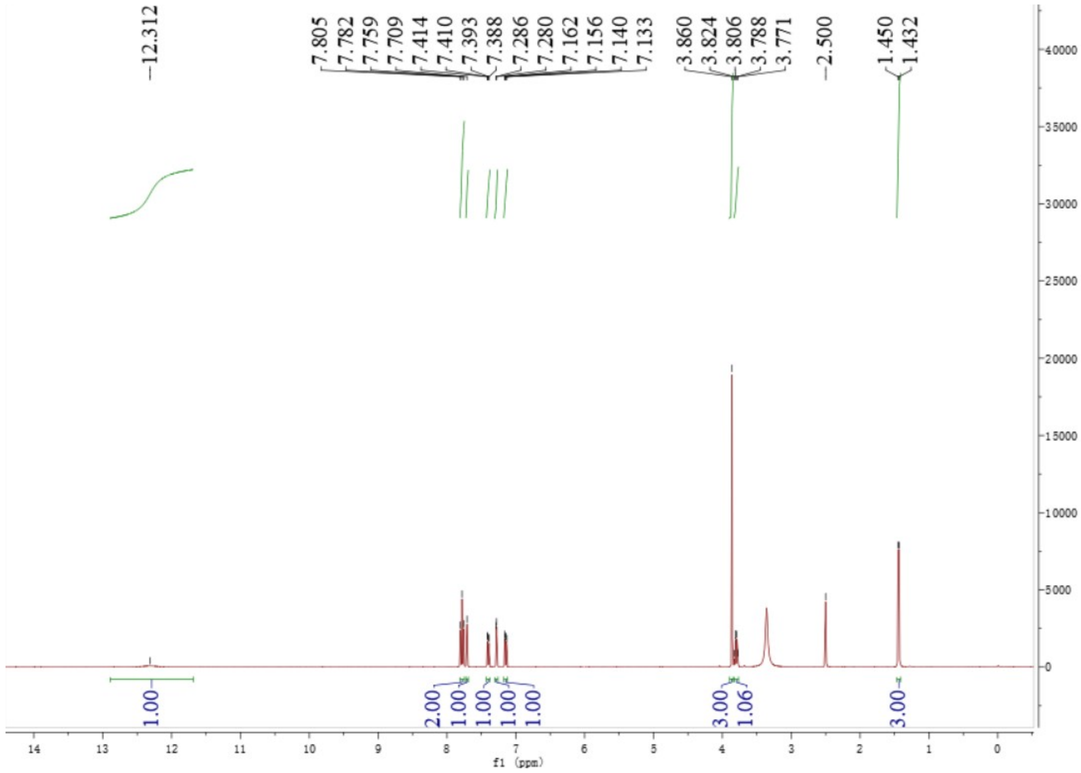
12.312
7.805
7.782
7.759
7.709
7.414
7.410
7.393
7.388
7.286
7.280
7.162
7.156
7.140
7.133
3.860
3.824
3.806
3.788
3.771
2.500
1.450
1.432
1.00
2.00
1.00
1.00
1.00
1.00
3.00
1.06
3.00
f1 (ppm)

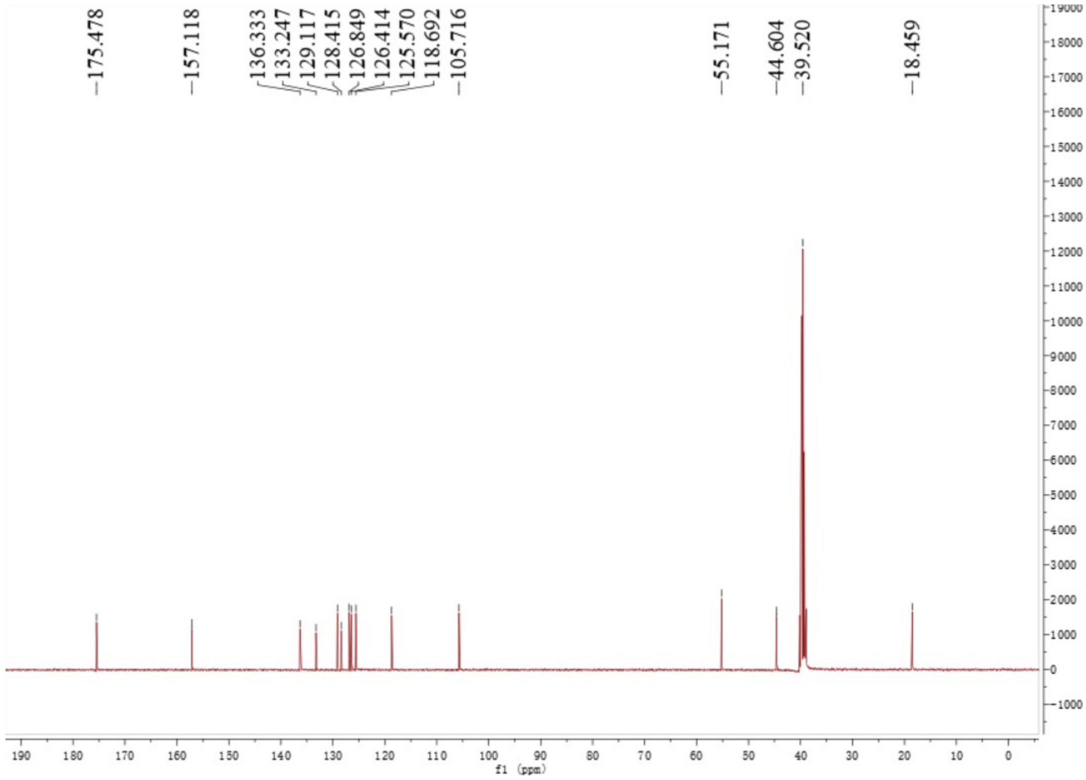
175.478
157.118
136.333
133.247
129.117
128.415
126.849
126.414
125.570
118.692
105.716
55.171
44.604
39.520
18.459
f1 (ppm)

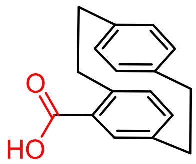
O
HO

4d

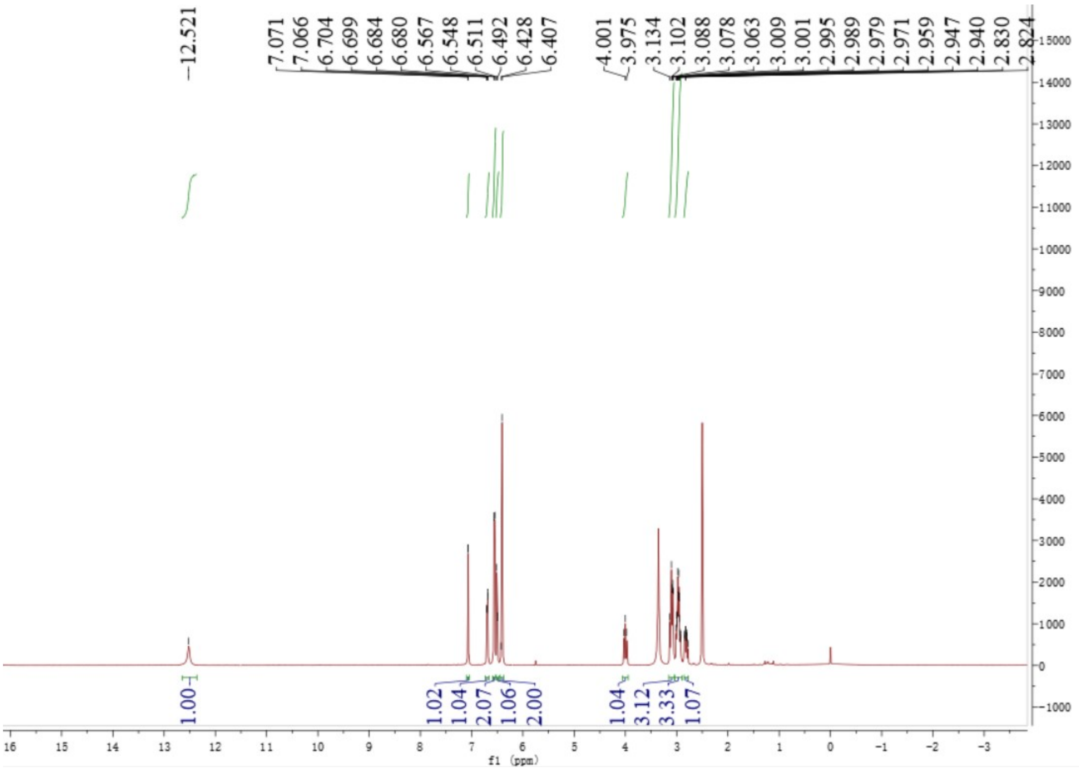
12.521
7.071
7.066
6.704
6.699
6.684
6.680
6.567
6.548
6.511
6.492
6.428
6.407
4.001
3.975
3.134
3.102
3.088
3.078
3.063
3.009
3.001
2.995
2.989
2.979
2.971
2.959
2.947
2.940
2.830
2.824
1.00
1.02
1.04
2.07
1.06
2.00
1.04
3.12
3.33
1.07
f1 (ppm)

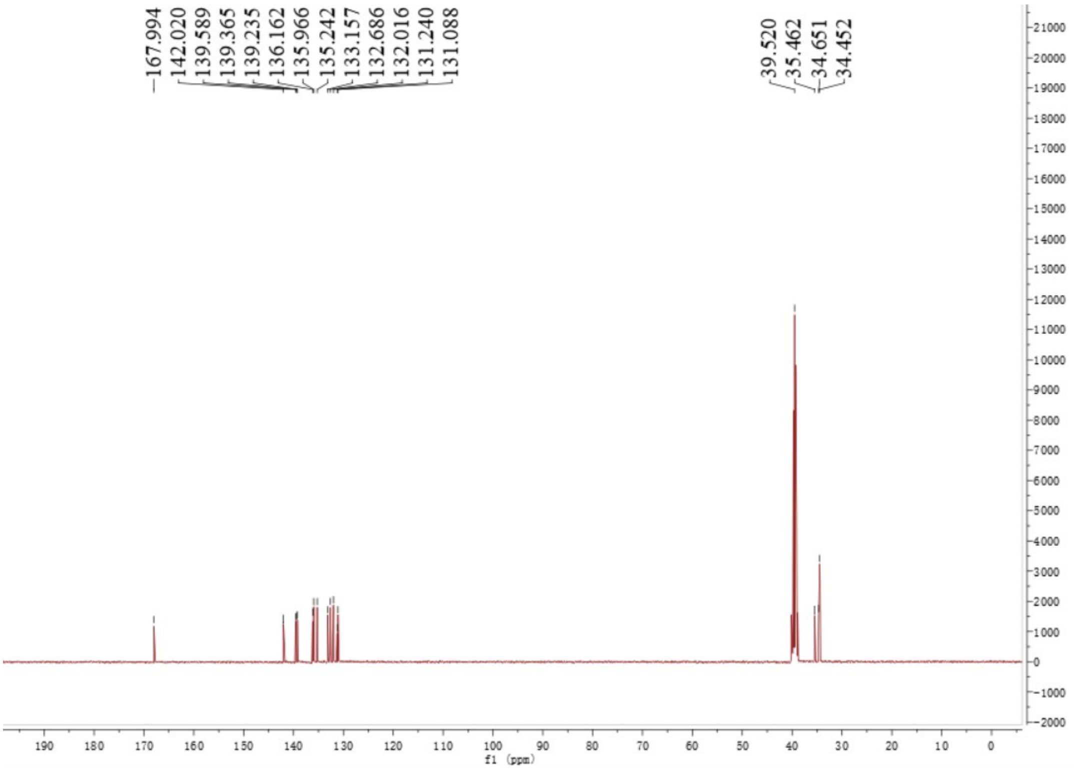
167.994
142.020
139.589
139.365
139.235
136.162
135.966
135.242
133.157
132.686
132.016
131.240
131.088
39.520
35.462
34.651
34.452
f1 (ppm)

Chromatographic data

The raw chromatographic data and yield calculations for the corresponding enzymatic reactions

Products	RT/min	Area	Yield/%
**2a**	4.387	8515683	97
**2b**	4.806	398206	97
**2c**	6.298	2521532	99
**2d**	7.725	13771930	95
**2e**	5.364	394276	98
**2f**	4.265	2034123	99
**2g**	3.125	1690243	25
**2h**	5.304	8954728	95
**2i**	6.839	5707359	91
**2j**	4.615	461375	85
**2k**	7.114	7671	58
**2l**	5.349	10169781	83
**2m**	6.905	3898236	92
**2n**	5.608	5501018	81
**2o**	8.611	6487953	90
**2p**	7.666	7797476	95
**2q**	6,922	7989115	96
**2r**	7.811	7454873	95
**2s**	5.203	1602150	95
**2t**	3.167	0.0133[a]	9
**2u**	8.29	0.1823[a]	99
**2v**	17.527	0.2523[a]	97
**4a**	18.076, 18.411	0.1809[a]	75
**4b**	38.357, 40.436	0.1829[a]	53
**4c**	10.488, 11.526	1372	20
**4d**	5.837, 7.350	1002	28

a. The area ratio of analytes to the internal standards.

4a, e.e. was established by GC analysis using a CP-7502 column; The temperature setting was programmed as follows: T_0: 120 ^oC; dT/dt: 1 ^oC/min, T_1: 180 ^oC. Retention time: t_r(minor) = 18.165 min, t_r(major) = 18.629 min.

Rac-**4a**

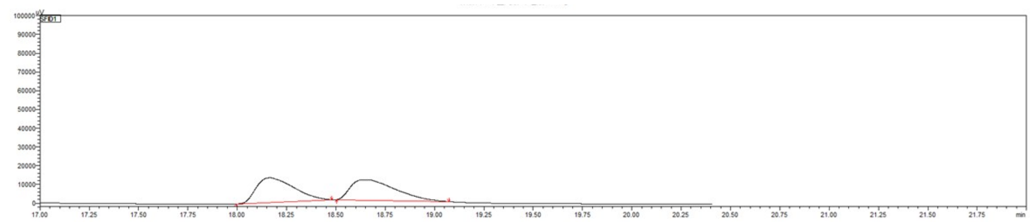

Peak	RT/min	Area	Area/%
1	18.165	173447	50.040
2	18.629	173168	49.960

R-**4a**

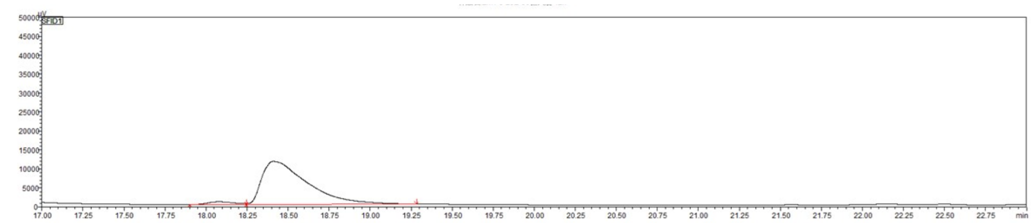

Peak	RT/min	Area	Area/%
1	18.076	8588	3.757
2	18.411	220010	96.243

4b, e.e. was established by GC analysis using a CP-7502 column; The temperature setting was programmed as follows: T_0: 120 °C; dT/dt: 1 °C/min, T_1: 180 °C. Retention time: t_r(major) = 38.357 min, t_r(minor) = 39.458 min.

Rac-**4b**

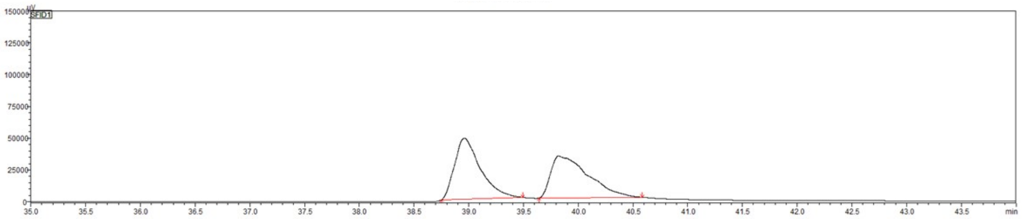

Peak	RT/min	Area	Area/%
1	38.894	882765	50.044
2	39.975	881218	49.956

R-**4b**

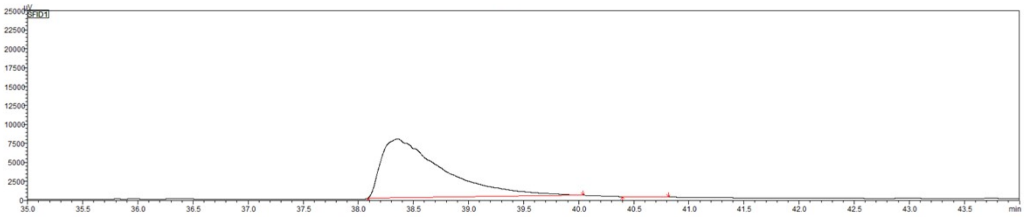

Peak	RT/min	Area	Area/%
1	38.357	303401	99.950
2	40.436	151	0.050

4c, e.e. was established by HPLC analysis using a Chiralcel AD-H column; HPLC conditions: 254 nm, n-hexane:isopropanol:trifluoroacetic acid= 90:10:0.1, flow rate 1.0 mL/min. Retention time: tr(minor) = 10.488 min, tr(major) = 11.526 min.

Rac-**4c**

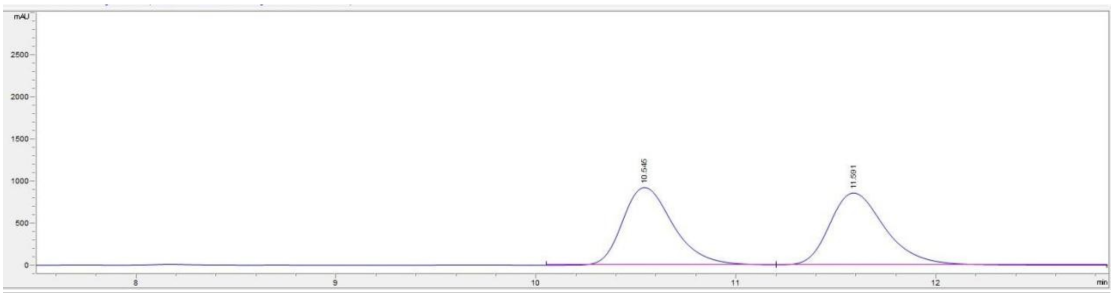

Peak	RT/min	Area	Area/%
1	10.545	16346	49.460
2	11.591	16703	50.540

R-**4c**

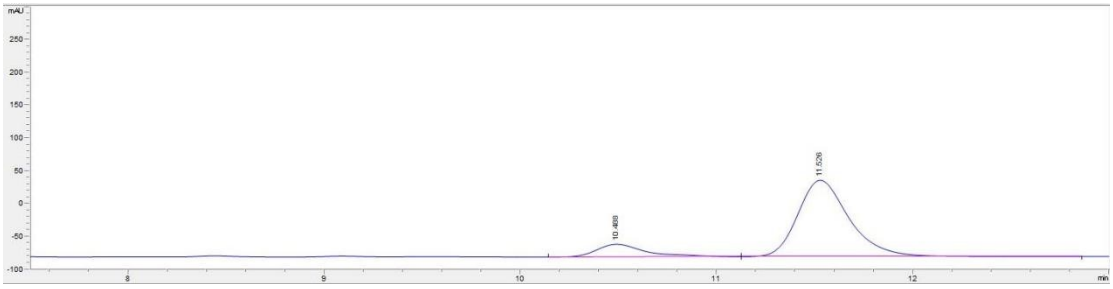

Peak	RT/min	Area	Area/%
1	10.488	342.1	13.897
2	11.526	2119.5	86.103

4d, e.e. was established by HPLC analysis using a Chiralcel AD-H column; HPLC conditions: 254 nm, n-hexane:isopropanol = 90:10, flow rate 1.0 mL/min. Retention time: tr(minor) = 5.873 min, tr(major) = 7.350 min.

Rac-**4d**

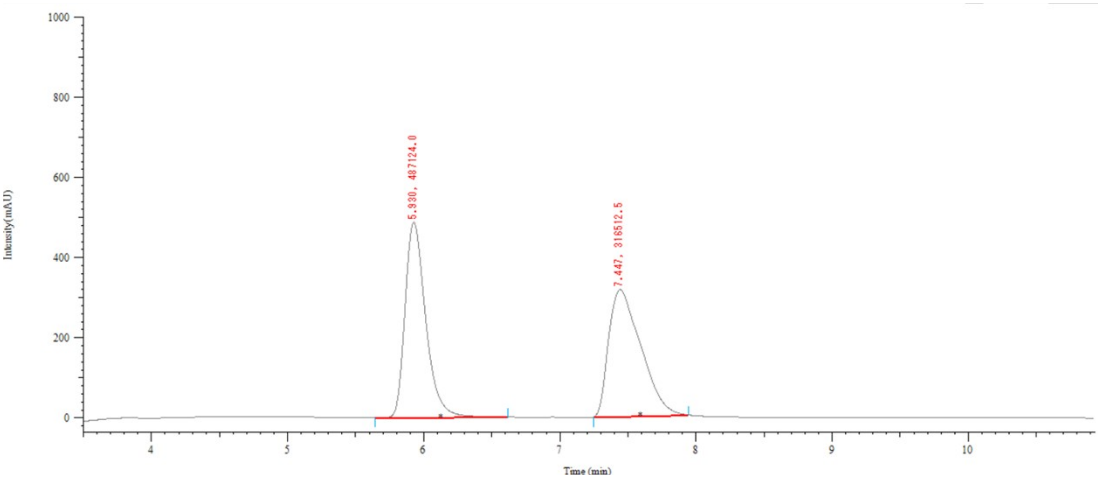

Peak	RT/min	Area	Area/%
1	5.930	5133104.5	49.806
2	7.447	5173158.2	50.194

R-**4d**

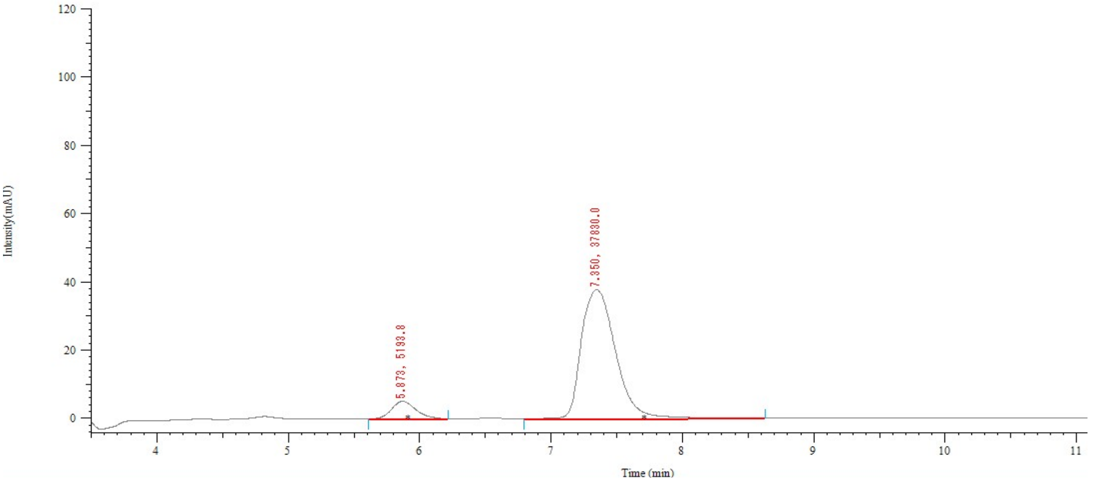

Peak	RT/min	Area	Area/%
1	5.873	67688.0	8.969
2	7.350	686985.8	91.031

References

[1] R. A. Friesner, J. L. Banks, R. B. Murphy, T. A. Halgren, J. J. Klicic, D. T. Mainz, M. P. Repasky, E. H. Knoll, M. Shelley, J. K. Perry, *Journal of Medicinal Chemistry* **2004**, *47*, 1739-1749.

[2] R. Salomon-Ferrer, D. A. Case, R. C. Walker, *Wiley Interdisciplinary Reviews: Computational Molecular Science* **2013**, *3*, 198-210.

[3] J. A. Maier, C. Martinez, K. Kasavajhala, L. Wickstrom, K. E. Hauser, C. Simmerling, Journal of Chemical Theory and Computation **2015**, *11*, 3696-3713.

[4] W. L. Jorgensen, J. Chandrasekhar, J. D. Madura, R. W. Impey, M. L. Klein, *The Journal of Chemical Physics* **1983**, *79*, 926-935.

[5] J. W. Ponder, D. A. Case, *Advances in Protein Chemistry* **2003**, *66*, 27-85.

[6] J. Wang, R. M. Wolf, J. W. Caldwell, P. A. Kollman, D. A. Case, *Journal of Computational Chemistry* **2004**, *25*, 1157-1174.

[7] M. Frisch, G. Trucks, H. Schlegel, G. Scuseria, M. Robb, J. Cheeseman, G. Scalmani, V. Barone, G. Petersson, H. Nakatsuji, *Gaussian 16, Revision B. 01.* Wallingford: Gaussian. Inc: **2016**.

[8] D. R. Roe, B. R. Brooks, *The Journal of Chemical Physics* **2020**, *153*, 054123.

[9] J. A. Izaguirre, D. P. Catarello, J. M. Wozniak, R. D. Skeel, *The Journal of Chemical Physics* **2001**, *114*, 2090-2098.

[10] R. Salomon-Ferrer, A. W. Gotz, D. Poole, S. Le Grand, R. C. Walker, *Journal of Chemical Theory and Computation* **2013**, *9*, 3878-3888.

[11] A. W. Gotz, M. J. Williamson, D. Xu, D. Poole, S. Le Grand, R. C. Walker, *Journal of Chemical Theory and Computation* **2012**, *8*, 1542-1555.

[12] J. P. Ryckaert, G. Ciccotti, H. J. Berendsen, *Journal of Computational Physics* **1977**, *23*, 327-341.

[13] D. R. Roe, T. E. Cheatham III, *Journal of Chemical Theory and Computation* **2013**, *9*, 3084-3095.
